# Structure‐Enabled Discovery of a Stapled Peptide Inhibitor to Target the Oncogenic Transcriptional Repressor TLE1

**DOI:** 10.1002/chem.201700747

**Published:** 2017-06-27

**Authors:** Sally McGrath, Marcello Tortorici, Ludovic Drouin, Savade Solanki, Lewis Vidler, Isaac Westwood, Peter Gimeson, Rob Van Montfort, Swen Hoelder

**Affiliations:** ^1^ The Institute of Cancer Research Division of Cancer Therapeutics Unit Cancer Research UK Cancer Therapeutics Unit 15 Cotswold Road, Sutton Surrey SM2 5NG UK; ^2^ Cancer Research (UK) Cancer Therapeutics Unit Division of Cancer Therapeutics Division of Structural Biology The Institute of Cancer Research 15 Cotswold Road, Sutton Surrey SM2 5NG UK; ^3^ Malvern Instruments Nordic AB Vallongatan 1 Uppsala 752 28 Sweden

**Keywords:** cancer, constrained peptide, inhibitors, protein–protein interactions, structure-based design

## Abstract

TLE1 is an oncogenic transcriptional co‐repressor that exerts its repressive effects through binding of transcription factors. Inhibition of this protein–protein interaction represents a putative cancer target, but no small‐molecule inhibitors have been published for this challenging interface. Herein, the structure‐enabled design and synthesis of a constrained peptide inhibitor of TLE1 is reported. The design features the introduction of a four‐carbon‐atom linker into the peptide epitope found in many TLE1 binding partners. A concise synthetic route to a proof‐of‐concept peptide, cycFWRPW, has been developed. Biophysical testing by isothermal titration calorimetry and thermal shift assays showed that, although the constrained peptide bound potently, it had an approximately five‐fold higher *K*_d_ than that of the unconstrained peptide. The co‐crystal structure suggested that the reduced affinity was likely to be due to a small shift of one side chain, relative to the otherwise well‐conserved conformation of the acyclic peptide. This work describes a constrained peptide inhibitor that may serve as the basis for improved inhibitors.

## Introduction

Transducin‐like enhancer (TLE) proteins are transcriptional co‐repressors that modulate key pathways for developmental and oncogenic signalling, such as the Notch and Wnt pathways. The TLE proteins do not bind directly to DNA to exert their repressive effect on gene transcription; instead, they utilise their WDR domains to bind to DNA‐bound transcription factors.^1^ Given their role in pathways known to be deregulated in many cancers, it is not surprising that members of the TLE family, particularly TLE1, have been implicated in the development and maintenance of malignancies. Elevated levels of TLE1 have been observed in a growing list of tumours, including cervical, lung and colon carcinomas, and TLE1 has been recognised as a putative oncogene.^2^ Given that TLE1 does not bind to DNA directly, and that its repressive and potentially oncogenic role relies on the ability of the WDR domain to bind to transcription factors, blocking of this interaction has been suggested as a possible treatment for cancers with elevated TLE1 activity.^3^ However, to date, no TLE inhibitors have been described in the literature.

The crystal structures of the WDR domain of TLE1 in complexes with peptides derived from two different transcription factor binding partners have been solved; thus characterising the binding interface in detail.^3^ One of these peptides (SMWRPW) shows relatively potent (*K*_d_=1 μm) binding to TLE1. As discussed in more detail below, the bioactive conformation of this peptide is characterised by a compactly folded core formed by the central three amino acids. This compact core engages in extensive interactions with the WDR1 domain and positions key amino acid side chains such that they can form additional polar and non‐polar interactions (see below).^3^ Given that this peptide binds with micromolar activity and that detailed knowledge of its binding mode and bioactive conformation are available, it represents an attractive starting point for the discovery of TLE inhibitors. Herein, we report a peptidomimetic approach based on the hypothesis that the compact conformation of this peptide can be stabilised by a hydrocarbon linker.

Hydrocarbon‐stapled macrocyclic peptides are increasingly being explored as drug candidates and chemical probes, particularly for challenging targets, such as protein–protein interactions.^4^ Introducing conformational constraint through macrocyclisation has a number of benefits. It particularly reduces the entropic penalty upon binding to the target and has been shown to have the potential to improve cell penetration and metabolic stability.^5^

Designing and synthesising constrained macrocyclic peptides still remains a formidable challenge.^4^ Nevertheless, successful examples have been reported, particularly for constraining and stabilising α helices, β sheets and β turns.^4^

However, in the case of TLE1, the bound SMWRPW peptide adopts neither a typical α‐helical nor a β‐sheet conformation, and constraining the peptide thus required a different strategy. As described in more detail below, we hypothesised that connecting two amino acids, the side chain of the first tryptophan and the proline—which were a critical part of the binding epitope—through a hydrocarbon linker would stabilise the bioactive conformation. Herein, we report the design and development of a chemical route to this hydrocarbon‐linker‐constrained, proof‐of‐concept peptide. Furthermore, we tested the binding affinity of the constrained and corresponding acyclic peptides and solved the structure of the constrained peptide bound to the WDR domain of TLE1.

## Results and Discussion

### Design

**Figure 1:**
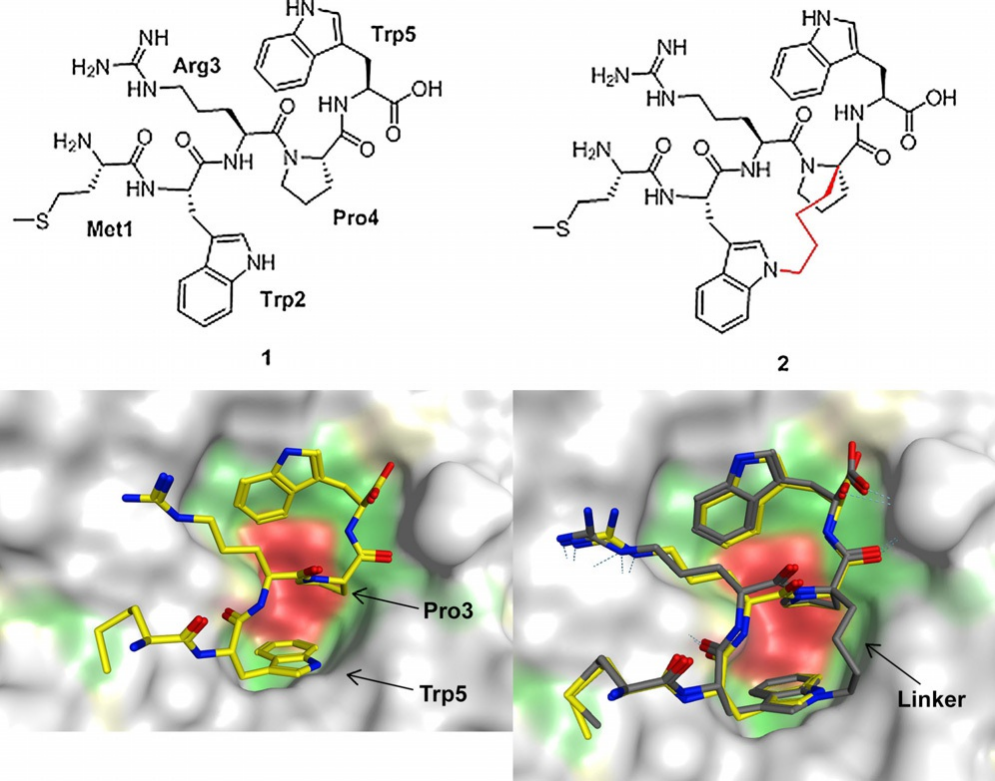
Top: Chemical structures of the SMWRPW peptide **1** and the constrained peptide **2** (the linker is drawn in red). Bottom: Co‐crystal structure of SMWRPW bound to the WDR domain of TLE1 (pdb code 2CE9) and superposition of the modelled binding pose of the constrained peptide with the crystallised pose of the unconstrained peptide **1**.

The crystal structure of the SMWRPW peptide bound to the WDR domain of TLE1 was obtained by soaking apo TLE1 crystals in a solution of the slightly extended SMWRPW peptide.^3^ The indole moiety of the N‐terminal tryptophan (Trp5) and the central proline (Pro3) of the bound peptide tightly pack against each other to form the core of the binding epitope (Figure 1). This core engages in extensive hydrophobic interactions with the protein.

The compact conformation positions side chains and backbone moieties of the peptide such that they are ideally placed to engage in additional polar and hydrophobic interactions.^3^ The N‐terminal serine residue of the SMWRPW peptide is not resolved, which suggests that it is disordered and does not make any specific interactions.

Our strategy to generate a constrained macrocyclic inhibitor is illustrated in Figure 1: we hypothesised that connecting the Cα‐atom of the proline residue and the N1 nitrogen of the N‐terminal tryptophan with a hydrocarbon linker would lock the peptide in the bioactive conformation. We modelled various linker lengths in MOE by introducing the linker in silico into the bound conformation of the peptide (PDB code 2CE9) and minimising the energy of modified peptides in the TLE binding site. The resulting poses were visually inspected for minimal movement of the peptide side chains and low‐energy conformations of the linker. These experiments, together with an analysis of synthetic accessibility (see below), suggested an ideal length of four carbon atoms and compound **2** (Figure 1) as a promising synthesis target.

### Retrosynthesis

**Scheme 1:**
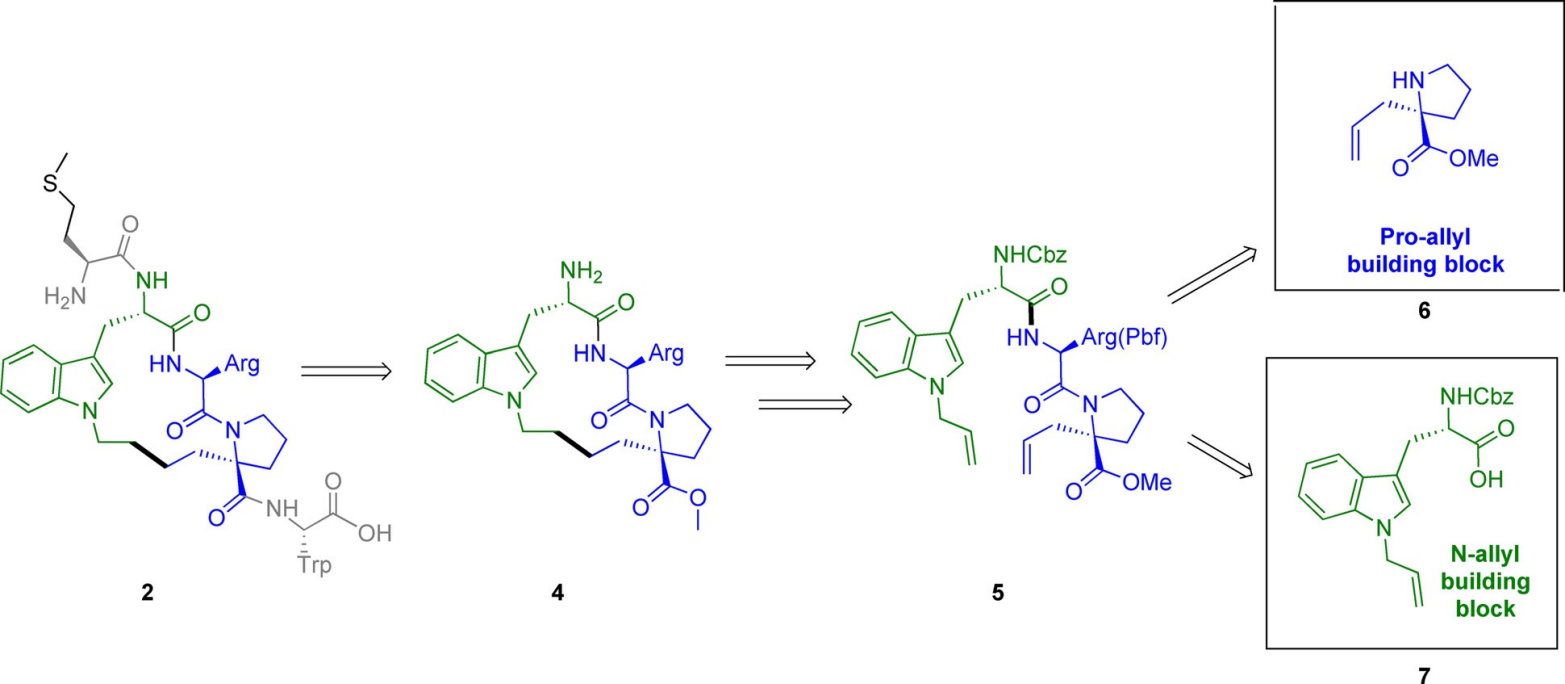
Retrosynthesis of constrained peptide **2**. Cbz=carboxybenzyl.

Our retrosynthetic analysis is depicted in Scheme 1. We envisioned synthesising the constrained hydrocarbon‐stapled peptide **2** from the macrocyclic intermediate **4** through addition of the N‐terminal methionine and C‐terminal tryptophan through peptide coupling chemistry. Furthermore, we hypothesised that intermediate **4** could be prepared from acyclic tripeptide **5** through ring‐closing metathesis (RCM), followed by concomitant saturation of the double bond and removal of the Cbz protecting group under hydrogenation conditions. To prepare the acyclic RCM precursor **5**, two unnatural amino acids were required: substituted proline **6** and allyl substituted tryptophan **7**.

This approach offered the advantage of conducting the critical RCM in solution, whilst all polar groups, particularly the basic arginine side chain, were fully protected. Furthermore, cyclic intermediate **4** offered the opportunity of late‐stage modification of the C‐ and N‐terminal amino acids.

### Synthesis and characterisation

**Scheme 2:**
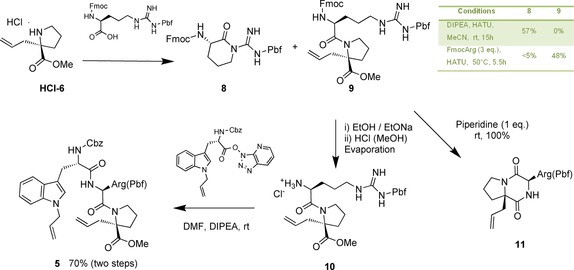
Synthesis of *N*‐allyl‐Trp **7**. DIPEA=*N*,*N*‐diisopropylethylamine, HOBT=hydroxybenzotriazole.

The synthesis of proline derivative **6** was described in the literature and we followed the protocols with minor modifications. We next turned our attention to the synthesis of Cbz‐protected 1‐allyl‐l‐tryptophan **7** (Scheme 2). We decided to use the Cbz protecting group because it was stable to basic and acidic conditions and because we anticipated that it could easily be removed during hydrogenation of the double bond arising from RCM; thus making an additional step unnecessary. At the start of this work, direct allylation of unprotected l‐tryptophan by using either a copper tetramethylethylenediamine (TMEDA) catalyst or sodium metal had been described.^6^ We tested the copper‐mediated conditions, but did not observe any conversion. More recently, a team from Sanofi published the synthesis of 1‐allyl‐l‐tryptophan protected with the *tert*‐butyloxycarbonyl (Boc) group, but this work was not in the public domain when we undertook our work.^7^ We hypothesised that selective allylation of unprotected tryptophan could be achieved after deprotonating the carboxyl group and the NH indole with two equivalents of a strong base, such as NaH, because under these conditions the deprotonated indole nitrogen represented the strongest nucleophile. Pleasingly, reacting l‐tryptophan with 2.5 equivalents of NaH and one equivalent of allyl bromide in DMF gave the desired mono‐allylated product in 40 % yield after HPLC purification. To avoid HPLC purification of the polar, unprotected amino acid, we decided to attempt allylation and subsequent Cbz protection with benzyl chloroformate in a one‐pot procedure. Gratifyingly, this procedure gave the desired, protected amino acid **7** in an acceptable yield of 28 % over two steps.

**Scheme 3:**
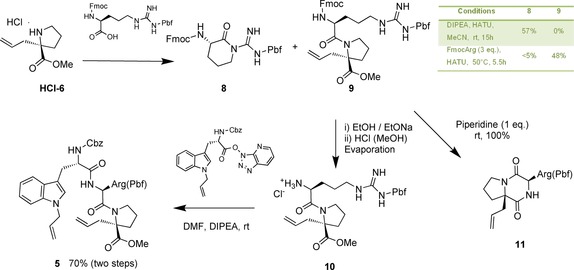
Optimised reaction conditions for the synthesis of arginine‐containing tripeptide **5**. HATU=1‐[bis(dimethylamino)methylene]‐1*H*‐1,2,3‐triazolo[4,5‐*b*]pyridinium 3‐oxid hexafluorophosphate, Fmoc=fluorenylmethyloxycarbonyl chloride.

We next prepared the tripeptide RCM precursor **5** by coupling the allylated proline with protected arginine (Scheme 3) under known conditions for this proline derivative. However, we only isolated the cyclised side product **8**. The formation of this side product is likely to be due to steric hindrance of the amine functionality. Gratifyingly, increasing the reaction temperature and concentrations of the reactants to favour biomolecular reaction led to the desired dipeptide in 48 % yield.

Next, we attempted removal of the Fmoc protecting group from dipeptide **9** (Scheme 3). However, standard conditions with piperidine as the base gave the undesired side product **11** as a single diastereomer.

Repeating the reaction with one equivalent of piperidine and at a lower temperature (0 °C) resulted in a mixture of the unprotected dipeptide and side product. Unfortunately, all attempts to isolate the unprotected dipeptide and to remove piperidine resulted in complete conversion to the diketopiperazine **11** side product. To solve this conundrum, we reasoned that protonation after Fmoc deprotection would lower the nucleophilicity of the free amino group sufficiently to prevent cyclisation; thus allowing isolation by evaporation of the solvent. Furthermore, we tested alternative bases, particularly bases that were not likely to affect the subsequent peptide coupling step. This approach indeed proved successful and compound **HCl‐10** was obtained as a single stereoisomer through clean Fmoc deprotection in EtOH by using one equivalent of NaOEt as a base. Subsequent protonation of the amine and residual traces of NaOEt through the addition of a solution of HCl in MeOH thwarted formation of the side product upon solvent evaporation (Scheme 3). Coupling of the crude product with the HATU derivative of the allyl‐substituted tryptophan **7** and DIPEA as a base yielded the metathesis precursor **5** in 70 % overall yield.

**Scheme 4:**
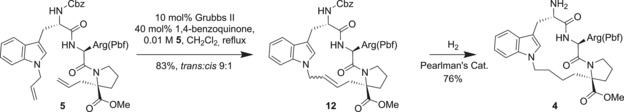
RCM of **5**, followed by removal of Cbz and double‐bond hydrogenation.

To our delight, the pivotal RCM proceeded readily by using the Grubbs second‐generation catalyst^8^ in the presence of 1,4‐benzoquinone^9^ to yield the desired product **12** in 83 % yield as a 9:1 mixture of *trans* and *cis* isomers (Scheme 4).

We next investigated concomitant reduction of the double bond and removal of the Cbz group by hydrogenation (Scheme 4). Commonly used conditions, such as 10 % palladium on carbon and hydrogen at atmospheric pressure, left the starting material intact. Elevated temperature, addition of acid or increase of catalyst loading did not significantly improve turnover. We next investigated other catalysts and found that the Pearlman catalyst both reduced the double bond and removed the Cbz protecting group. Complete conversion required one equivalent of Pd(OH)_2_/C and the addition of two equivalents of HCl, but resulted in a yield of 76 % of the reduced and deprotected intermediate being isolated.

**Scheme 5:**
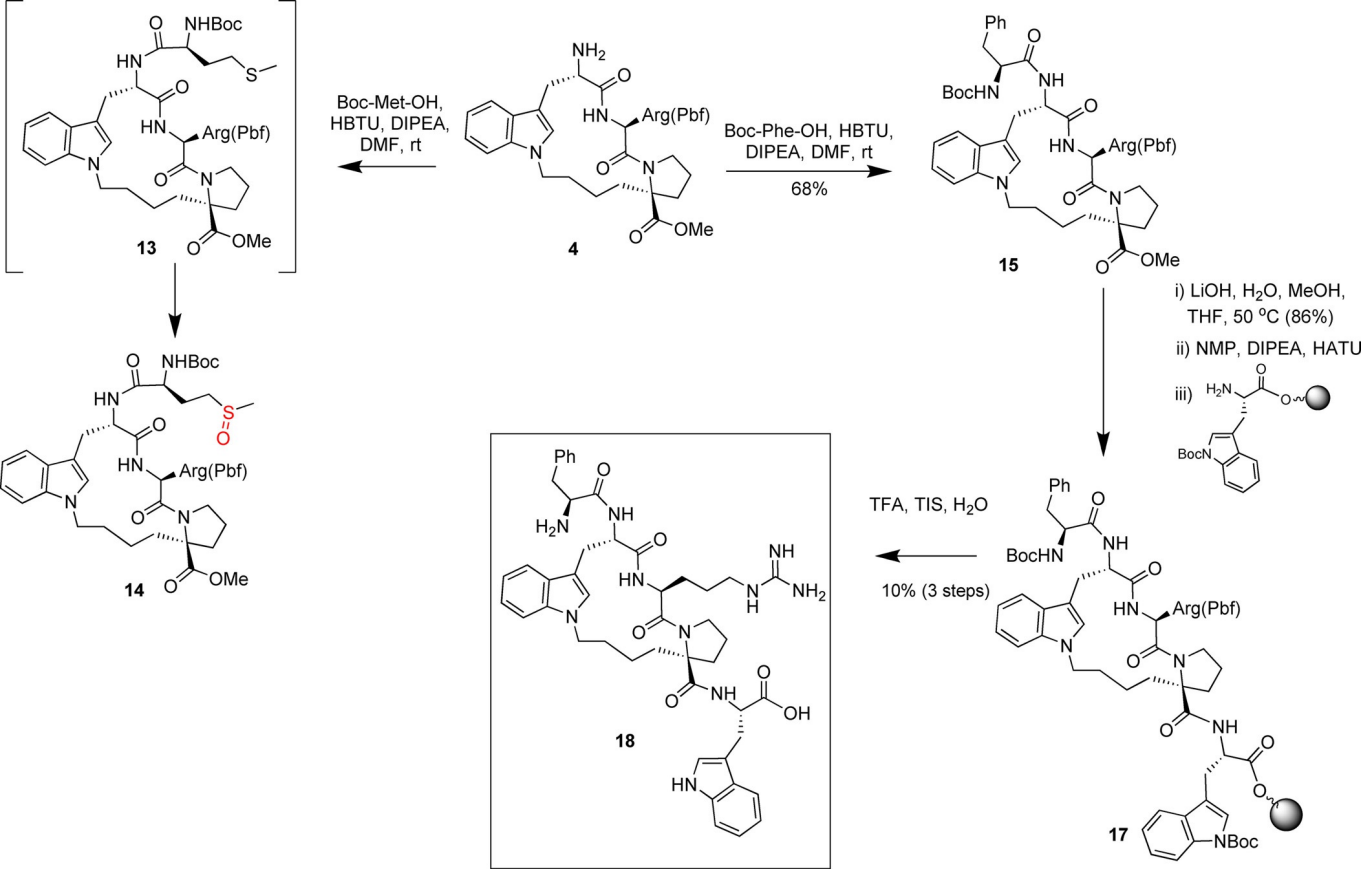
Synthesis of the final constrained peptide **18**. NMP=*N*‐methyl‐2‐pyrrolidone, TFA=trifluoroacetic acid, TIS=triisopropylsilane.

With intermediate **4** in hand, we next performed coupling to Boc‐protected methionine. Although this coupling proceeded readily, we reproducibly observed a +16 Da increase in molecular weight after isolation and purification. We attributed this increase to oxidation of methionine to the corresponding sulfoxide derivative **14** (Scheme 5). This oxidation has precedent in the literature; however, the degree and rapidness of the reaction is surprising, given that methionine is frequently incorporated into peptides.

As we discuss in more detail below, this methionine residue can be replaced in the acyclic peptide by phenylalanine without loss of activity. We thus focused our attention on the phenylalanine derivative. Coupling of **4** with Boc‐protected phenylalanine proceeded in 68 % yield after purification (Scheme 5).

To complete the synthesis, we hydrolysed the ester by using LiOH in methanol (86 % yield) and added the final amino acid by coupling this intermediate onto tryptophan bound to a commercially available solid support (Scheme 5).

Cleavage of the solid support of **17** and concomitant removal of the remaining two protecting groups provided the desired macrocyclic peptide **18** in 10 % yield over three steps (Scheme 5).

Despite initial challenges, our synthetic approach enabled us to access 14 mg of the desired, constrained peptide. Some of the optimised steps, for example, the one‐pot alkylation and protection of tryptophan, as well as the convenient and mild deprotection of the Fmoc group in solution, may be useful for the synthesis of other constrained peptides.

**Figure 2:**
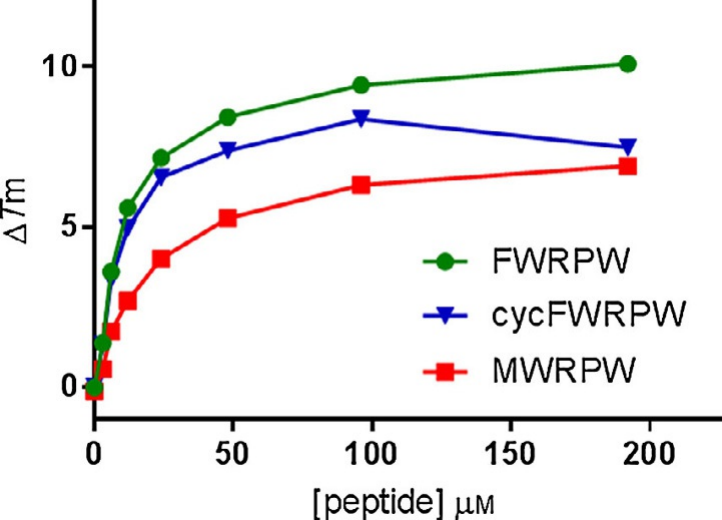
Δ*T*_m_ plot of peptide–hTLE1 443–770 interactions in thermal shift experiments. All measurements were carried out in triplicate and the points are reported as mean+standard deviation (SD). The values of Δ*T*_m_ at the top concentrations are also reported Table 1.

We next investigated the binding of this macrocycle, as well as acyclic MWRPW and FWRPW peptides, to TLE1. We used two orthogonal binding assays, the thermal shift assay^10^ and isothermal titration calorimetry (ITC),^11^ to test binding of **18** and the linear peptides to the TLE1 WD40 domain (TLE1 residues 443–770). The thermal shift data for the three peptides are shown in Figure 2 and Table 1.

**Table 1:** Thermal shifts at peptide concentrations of 100 and 200 μm.

Peptide	Δ*T*_m_ [°C]	
ligand	100 μm	200 μm
MWRPW	6.3	6.9
cycFWRPW (**18**)	8.4	7.5
FWRPW	9.4	10.1

All three peptides showed significant thermal shifts that were indicative of binding to the protein. Interestingly, the MWRPW peptide, which is derived from the sequence of TLE1 binding partners, shows the smallest thermal increase. The mutant FWRPW peptide causes a significantly larger thermal shift (9.4 versus 6.3 °C). The cyclic peptide cycFWRPW (**18**) at 100 μm shows a thermal shift comparable to that of the corresponding acyclic peptide (Table 1). However, the thermal shift decreases when the concentration is further increased from 100 to 200 μm. This decrease is likely to be due to precipitation of the peptide at higher concentrations. Our thermal shift data thus suggested that all three peptides bound to TLE1.

To confirm these findings and to explore the enthalpic and entropic contributions to binding of the linear and constrained peptides, we performed ITC experiments. Given conformational restriction, one might expect the constrained peptide to show a smaller entropic penalty upon binding. However, all three peptides showed potent binding driven by strong enthalpy contributions.

**Figure 3:**
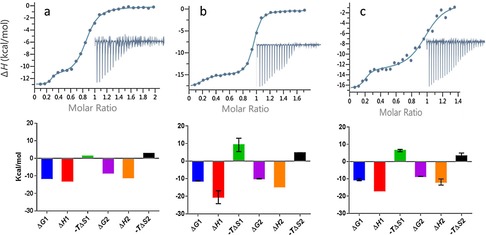
Top: ITC measurements of peptide–TLE1 binding interactions. Data fitting to a two‐site independent binding model are shown; integrated heats are shown in the inset. Bottom: Histograms showing Δ*G*, Δ*H*, and −*T*Δ*S*. The thermodynamic values are also presented in Table 1. a) MWRPW–TLE1 binding, *n*=1. Experiment performed with TLE1 (40 μm) and MWRPW peptide (420 μm). b) FWRPW–TLE1 binding, *n*=2. Experiment performed with TLE1 (30 μm), FWRPW peptide (240 μm), and then repeated with TLE1 (24 μm), FWRPW peptide (180 μm). Histograms represent averaged values; error bars denote SD. c) **18**–TLE1 binding, *n*=2. Experiment performed with TLE1 (30 μm), **18** (200 μm) and then repeated with TLE1 (30 μm), **18** (180 μm). Histograms represent averaged values; error bars denote SD.

Interestingly, for each peptide we observed a biphasic curve. This was initially more pronounced for FWRPW and **18**, but also recognisable for the MWRPW peptide (see Figure S1 in the Supporting Information). We repeated the MWRPW titration at slightly higher protein and peptide concentrations to achieve a higher enthalpy signal, and therefore, better resolution of the titration event. Under these conditions, we also observed a clear biphasic curve (Figure 3). The biphasic curves are indicative of two binding events and we calculated the thermodynamic data for both (Table 2).

**Table 2:** The *K*_d_ and thermodynamic values determined by ITC for all peptide–TLE1 binding experiments.

	N1	*K*_d_1 [nm]	Δ*H*1 [kcal mol^−1^]	−*T*Δ*S*1 [kcal mol^−1^]	N2	*K*_d_2 [nm]	Δ*H*2 [kcal mol^−1^]	−*T*Δ*S*2 [kcal mol^−1^]
MWRPW	0.24±0.001	3.5±1.9	−12.8±0.2	1.3	0.6±0.004	772±7.1	−11.1±0.11	2.72
FWRPW	0.18±0.002	8.6±3.9	−20.5±3.7	9.2±3.8	0.7±0.004	79.4±24.6	−14.6±0	4.6±0.2
**18**	0.18±0.01	24.7±24.2	−16.9±0	6.4±0.6	0.8±0.01	522±39.6	−11.8±1.8	3.2±1.7

The first phase of the curves corresponded to a molar ratio of approximately 0.2 (that is, 20 % of the protein is bound) and the second phase corresponded to an approximate molar ratio of 0.8; thus, the overall curve reached saturation at a molar ratio close to 1. This suggested that only one binding site per molecule of protein was occupied by the ligand. A possible explanation for the biphasic curve is that in the binding experiment the protein exists in two conformations which do not rapidly interconvert and show different binding affinities for the peptides. The observation that the molar ratios for the two parts of the biphasic curve correspond to different peptides is in agreement with this hypothesis.

In the following paragraph, we focus the discussion of the ITC results on the second binding event (*K*_d_2, Δ*H*2 and −*T*Δ*S*2) for three reasons. The second binding event covers binding to the large majority of the protein (≈80 %); the *K*_d_ values are in agreement with published values; and, finally, due to the experimental set up, the relative errors are smaller. However, we include data for the first binding event (*K*_d_1, Δ*H*1 and −*T*Δ*S*1) and they broadly follow the same trend.

The rank order based on the ITC *K*_d_ values (*K*_d_2) confirms the rank order from the thermal shift assay described above. The acyclic FWRPW peptide shows the highest affinity with a *K*_d_ of 79 nm. It thus shows almost 10‐fold more potent binding than the peptide representing the original MWRPW sequence from the TLE1 binding partners. The *K*_d_2 value for cyclic peptide **18** is 522 nm and thus less potent than the corresponding acyclic peptide, which suggests that introduction of the hydrocarbon linker leads to a small loss of activity. Interestingly, binding of **18** is accompanied by a reduced loss of entropy compared with the acyclic peptide, which is in agreement with the hypothesis that the introduction of a constraint reduces the entropic penalty (albeit that this reduced entropic loss is overcompensated for by a larger enthalpic loss, leading to a higher *K*_d_ compared with that of the acyclic FWRPW peptide).

To be able to interpret these thermodynamic data in light of the binding modes, we set out to determine the crystal structure of the cyclic peptide **18**. Briefly, we grew apo crystals of the TLE1 WD40 domain by using slightly modified previously published conditions^3^ and succeeded in solving the structure of cyclic peptide **18** bound to TLE1 to 2.18 Å resolution by soaking with a 2.5 mm solution of **18**. The asymmetric unit contained two TLE1 monomers and the electron density was evident in both binding sites. However, the quality of the electron density differed in the two independent TLE1 monomers. Chain A showed strong ligand density and allowed us to model cyclic peptide **18** with full occupancy. The ligand density in chain B was weaker and refined at a lower occupancy (0.83). Therefore, we focus the discussion on the peptide bound to chain A.

**Figure 4:**
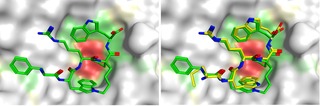
Left: Co‐crystal structure of constrained peptide **18** bound to the WDR domain of TLE1. Right: Super‐positioning of the acyclic (yellow) and cyclic (green) peptides **1** and **18**.

Figure 4 depicts the constrained peptide bound to TLE1 and an overlay with the published structure of our acyclic design template, SMWRPW (pdb code 2CE9).

Overall, the binding mode of the constrained peptide is almost identical to that of the published acyclic peptide‐bound structure. The overall root‐mean‐square deviation (RMSD) between the two structures is 0.55 Å. The N‐terminal phenylalanine side chain of the constrained peptide occupies a similar position to that of the methionine side chain, with the aromatic side chain efficiently packing against the hydrophobic part of Glu 550; this potentially explains the slightly higher affinity of acyclic FWRPW, compared with that of the MWRPW peptide. The most significant difference between the cyclic peptide conformation and the bound SMRWPW conformation is the linker, which appears to cause a slight change in position of the N‐terminal tryptophan and could go some way to explain the lower affinity of **18** compared with that of the linear FWRPW peptide. This slight movement may create an unfavourable, modestly repulsive, interaction that outweighs the gain achieved through constraining the peptide. The observation that our cyclic peptide shows a higher *K*_d_ value, despite replicating the bioactive conformation very accurately, underscores the challenge of designing constrained peptides. Minor differences that are outside the predictive power of current structure‐based design tools, even if high‐resolution crystal structures are available, can have a significant effect on bioactivity.

## Conclusion

We developed a concise synthetic route to a constrained proof‐of‐concept peptide **18**. Biophysical analysis by ITC and thermal shift assays and X‐ray crystallography confirmed that the constrained peptide bound to the WD40 domain of TLE1. Furthermore, the observation that the constrained peptide shows binding thermodynamics that are entropically favoured, relative to the acyclic FWRPW peptide, is in agreement with the hypothesis that rigidifying the peptide lowered the entropic penalty upon binding to the target. However, the constrained peptide also showed an approximately six‐fold lower affinity than that of the acyclic peptide. The crystal structure of the constrained peptide bound to TLE1 suggests that the linker causes some strain in the molecule that may, at least partially, explain the lower affinity. These observations underscore the known challenge of designing constrained peptides. Our constrained peptide replicated the bioactive conformation very well with an RMSD of 0.55 Å and yet a slight deviation caused a sufficient penalty to compensate for the gain achieved by introducing the constraint.

## Experimental Section

Experimental and characterisation details for all new compounds, computational data, ITC data, assays data, crystallographic data, and NMR spectra are provided in the Supporting Information.

### Accession codes

Atomic coordinates and structure factors for the crystal structure of TLE1 with constrained peptide **18** can be accessed by using PDB code 5MWJ.

## Conflict of interest

The authors declare no conflict of interest.

## Supporting information

As a service to our authors and readers, this journal provides supporting information supplied by the authors. Such materials are peer reviewed and may be re‐organized for online delivery, but are not copy‐edited or typeset. Technical support issues arising from supporting information (other than missing files) should be addressed to the authors.

Supplementary
