# Supplementary material for: Structure‐Enabled Discovery of a Stapled Peptide Inhibitor to Target the Oncogenic Transcriptional Repressor TLE1
Source: Chemistry. 2017 Jun 27;23(40):9577–84. doi: 10.1002/chem.201700747 (PMC5575474; doi:10.1002/chem.201700747)
Supplement: Supplementary file 1 — Supplementary [file CHEM-23-9577-s001.pdf]

CHEMISTRY
A European Journal

Supporting Information

Structure-Enabled Discovery of a Stapled Peptide Inhibitor to Target the Oncogenic Transcriptional Repressor TLE1

Sally McGrath,[a] Marcello Tortorici,[a] Ludovic Drouin,[a] Savade Solanki,[a] Lewis Vidler,[a] Isaac Westwood,[a] Peter Gimeson,[c] Rob Van Montfort,[b] and Swen Hoelder*[a]

chem_201700747_sm_miscellaneous_information.pdf

Table of contents

General Experimental S5

Cloning, Expression and Purification of humanTLE1 443-770 S6

Peptides S8

Thermal Shift Assay S8

ITC S8

Supplementary figure S10

Characterisation of compounds S11

Hydrogenation Product (4) S11

(*R*)-Methyl 2-allyl-1-((*S*)-2-((*S*)-3-(1-allyl-1*H*-indol-3-yl)-2-(((benzyloxy)carbonyl) amino) propanamido)-5-(3-((2,2,4,6,7-pentamethyl-2,3-dihydrobenzofuran-5-yl) sulfonyl)guanidino) pentanoyl)pyrrolidine -2-carboxylate (5) S12

(*R*)-Methyl 2-allylpyrrolidine-2-carboxylate, HCl salt (6-HCl), (*R*)-2-Allylpyrrolidine-2-carboxylic acid, HCl salt (37) and (*R*)-Methyl 2-allylpyrrolidine-2-carboxylate (6) S13

(*S*)-3-(1-Allyl-1*H*-indol-3-yl)-2-(benzyloxycarbonylamino)propanoic acid (7) S15

(*S*)-(9*H*-Fluoren-9-yl)methyl 2-oxo-1-(*N*-(2,2,4,6,7-pentamethyl-2,3-dihydrobenzofuran-5-ylsulfonyl)carbamimidoyl)piperidin-3-ylcarbamate (8) S16

(*R*)-Methyl 1-((*S*)-2-(((9H-fluoren-9-yl)methoxy)carbonylamino)-5-(3-(2,2,4,6,7-pentamethyl-2,3-dihydrobenzofuran-5-ylsulfonyl)guanidino)pentanoyl)-2-allylpyrrolidine-2-carboxylate (9) S17

(*R*)-Methyl 2-allyl-1-((*S*)-2-amino-5-(3-(2,2,4,6,7-pentamethyl-2,3-dihydrobenzofuran-5-ylsulfonyl)guanidino)pentanoyl)pyrrolidine-2-carboxylate (10) and *N*-(*N*-(3-((3*R*,8a*R*)-8a-Allyl-1,4-dioxooctahydropyrrolo[1,2-*a*]pyrazin-3-yl)propyl)carbamimidoyl)-2,2,4,6,7-pentamethyl-2,3-dihydrobenzofuran-5 -sulfonamide (11) S18

Metathesis Product (12) S20

Methionine Coupling Product (13) and Oxidised Form (14) S22

Phenylalanine Coupling Product (15) **S23**
Ester Hydrolysis Product (16) **S24**
Final Constrained Peptide, C-terminal acid (18) **S25**
1*H*-Indole-3-carbaldehyde (19) **S26**
***tert*-Butyl 3-formyl-1*H*-indole-1-carboxylate (20)** **S27**
***tert*-Butyl 3-(hydroxymethyl)-1*H*-indole-1-carboxylate (21)** **S28**
***tert*-Butyl 3-(bromomethyl)-1*H*-indole-1-carboxylate (22)** **S29**
(*R*)-3,6-Diethoxy-2-isopropyl-2,5-dihydropyrazine (23) **S29**
***tert*-Butyl 3-(((2*S*,5*R*)-3,6,diethoxy-5-isopropyl-2,5-dihydropyrazin**
-2-yl)methyl)-1*H*-indole-1-carboxylate (24) **S30**
***tert*-Butyl 2-bromo-3-methyl-1*H*-indole-1-carboxylate (25)** **S31**
***tert*-Butyl 2-bromo-3-(bromomethyl)-1*H*-indole-1-carboxylate (26)** **S32**
(5*R*)-2-Benzyl-3,6-diethoxy-5-isopropyl-2,5-dihydropyrazine (27) **S33**
***tert*-Butyl 2-bromo-3-(((2*S*,5*R*)-3,6-diethoxy-5-isopropyl-2,5**
-dihydropyrazin-2-yl)methyl)-1*H*-indole-1-carboxylate (28)
and *tert*-Butyl 2-bromo-3-(((2*R*,5*R*)-3,6-diethoxy-5-isopropyl-2,5
-dihydropyrazin-2-yl)methyl)-1*H*-indole-1-carboxylate (29) **S36**
***tert*-Butyl 3-((*tert*-butoxycarbonyloxy)methyl)-1*H*-indole-1-carboxylate (30)**
and 1-Allyl-1*H*-indole-3-carbaldehyde (31) **S37**
***tert*-Butyl 3-((allyloxycarbonyloxy)methyl)-1*H*-indole-1-carboxylate (32)** **S39**
***S*)-3-(1-Allyl-1*H*-indol-3-yl)-2-aminopropanoic acid (33)** **S40**
(3*R*,7a*R*)-7a-Allyl-3-(trichloromethyl)tetrahydropyrrolo[1,2-*c*]oxazol
-1(3*H*)-one (34) **S41**
(3*R*,7a*R*)-1-Oxo-3-(trichloromethyl)hexahydropyrrolo[1,2-c]oxazole
-7a-carbaldehyde (35) **S42**
(3*R*,7a*R*)-3-(Trichloromethyl)-7a-vinyltetrahydropyrrolo[1,2-c]oxazol
-1(3*H*)-one (36) **S42**

NMR Spectra S44

LC/MS Spectra of TLE1 and tested peptides S79

X-Ray crystallography data collection, refinement S84

References S85

General Experimental

Unless otherwise stated, reagents and solvents were purchased from commercial suppliers (AAPPTEC, Abgent, Acros, Advanced ChemTech, Alfa Aesar, Apollo, Applied Biosystems, Avocado, ChemBridge, Fisher, Fluorochem, Generon, Merck Chemicals, Novabiochem, Pepceuticals, Sigma-Aldrich and VWR) and used without further purification. Chromatography solvents were HPLC grade and were used without further purification. All reactions were carried out in oven-dried flasks under a positive pressure of N_2, and air- and moisture-sensitive reagents transferred *via* syringe. Brine refers to saturated aqueous solution of NaCl. The term concentrated *in vacuo* refers to rotary evaporation.

Normal phase thin layer chromatography was conducted on standard commercial aluminium sheets pre-coated with a 0.2 mm layer of silica gel (Merck 60-254), and normal phase flash column chromatography was performed on silica gel 40 – 63 µm (Fluka 40 or Geduran 60) or on pre-packed Biotage SNAP columns. Reversed-phase thin layer chromatography was conducted on glass plates pre-coated with a 0.2 mm layer of KC18F reversed-phase silica (Whatman 4803-600) and reversed-phase flash column chromatography was performed on a pre-packed 30 g Biotage SNAP C-18 column.

Final peptides (and *trans* isomer of metathesis product) were made up to 7.5-14 mg/mL solutions in either MeOH or a MeOH/H_2O mix and purified by semi-preparative RP-HPLC using one of the following sets of conditions:

A) 500-1000 µL standard injections (with needle rinse) of the sample were made onto a Phenomenex Gemini column (5 µm, 250 x 10 mm, C18, Phenomenex, Torrance, USA). Chromatographic separation at room temperature was carried out using a 1200 Series Preparative HPLC (Agilent, Santa Clara, USA) with the elutions reported under each peptide entry below. UV-Vis spectra were acquired at 254 nm and 280 nm on a 1200 Series Prep Scale diode array detector (Agilent, Santa Clara, USA). Collection was triggered by timed fractions, and collected on a 1200 Series Fraction Collector (Agilent, Santa Clara, USA). Raw data were processed using Agilent Chemstation Software.

B) 500-1000 µL standard injections (with needle rinse) of the sample were made onto a Phenomenex Luna column (10 µm, 250 x 21.2 mm, C18, Phenomenex, Torrance, USA). Chromatographic separation at room temperature was carried out using a Gilson GX-281 Liquid Handler system combined with a Gilson 322 HPLC pump (Gilson, Middleton, USA) with the elutions reported under each peptide entry below. UV-Vis spectra were acquired at 254 nm on a Gilson 156 UV-Vis detector (Gilson, Middleton, USA). Collection was triggered

by UV signal, and collected using a Gilson GX-281 Liquid Handler system (Gilson, Middleton, USA). Raw data were processed using Gilson Trilution Software.

^{1}H NMR spectra were recorded on a Bruker Avance 500 MHz spectrometer using an internal deuterium lock. Chemical shifts were measured in parts per million (ppm) relative to tetramethylsilane (TMS, δ = 0) and were referenced to the following residual solvent signals: $CHCl_3$ (δ 7.26), CD_2HOD (δ 3.32), DHO (δ 4.79) and $(CD_3)(CD_2H)SO$ (δ 2.50). Data are presented in the following format: chemical shift (multiplicity, coupling constants (*J* in Hz, order corresponds to order of multiplicities reported), integration, assignment). Atom numbering is arbitrary and does not refer to IUPAC nomenclature.

^{13}C NMR spectra were recorded on a Bruker Avance 500 MHz spectrometer using an internal deuterium lock. Chemical shifts were measured in parts per million (ppm) relative to tetramethylsilane (TMS, δ = 0) and were referenced to the following residual solvent signals: $CHCl_3$ (δ 77.16), CD_2HOD (δ 49.00) and $(CD_3)(CD_2H)SO$ (δ 39.52). Data are presented in the following format: chemical shift (assignment). Atom numbering is arbitrary and does not refer to IUPAC nomenclature.

LCMS analyses and high resolution mass spectrometry were performed on an Agilent 1200 series HPLC and diode array detector coupled to a 6210 time of flight mass spectrometer with dual multimode APCI/ESI source. Samples were supplied as approximately 1 mg/mL solutions in MeOH or $CHCl_3$ with 0.5-10 µL injected on a partial loop fill. Analytical separation was carried out at 30 °C on either a Merck Chromolith SpeedROD column (RP-18e, 50 x 4.6 mm) using a flow rate of 2 mL/min or a Merck Purospher STAR column (RP-18e, 30 x 4 mm) using a flow rate of 1.5 mL/min. Detection was at 254 nm. Molecular weight scan range was 85 – 950, 160 – 950 or 160 – 1700. HRMS references: **caffeine** $[M+H]^+$ 195.08765; **reserpine** $[M+H]^+$ 609.28066 or **hexakis (2,2-difluroethoxy)phosphazene** $[M+H]^+$ 622.02896; and **hexakis(1*H*,1*H*,3*H*-tetrafluoropentoxy)phosphazene** $[M+H]^+$ 922.00980.

The gradients for each method were as follows, with MeOH as eluent A and 0.1% formic acid in water as eluent B.

Fast4min:

Time / min	A (%)	B (%)
0	10	90

Fast4minLipophilic:

Time / min	A (%)	B (%)
0	10	90

2.5	90	10
3.5	90	10
3.8	10	90
4	10	90

1	100	0
3.5	100	0
3.8	10	90
4	10	90

Melting points were determined on a Reichert Thermovar melting point apparatus and are uncorrected. Optical rotations were recorded on a Bellingham & Stanley Ltd. ADP440 Polarimeter with a path length of 0.5 dm, using a light emitting diode with interference filter (289 nm). Concentrations (*c*) are quoted in g / 100 mL. IR analyses were carried out on a Bruker Alpha-P FT-IR spectrometer and absorptions are specified in wavenumbers (cm^{-1}). Elemental analyses were determined by the London Metropolitan University Analytical Service (Stephen Boyle).

In silico modifications were performed manually from the Pearl group's experimentally observed SMWRPW pose using the builder tab in MOE 2012.10.[1] Glide refinement was performed using standard precision mode with the GlideScore scoring function. For the receptor, PDB structure 2CE9 was processed using the Protein Preparation Wizard in Maestro.[2] A grid box of 20 Å length centred on the ligand was used. Default parameters were used with up to 5 poses generated per ligand.

Cloning, Expression and Purification of humanTLE1 443-770.

The construct encoding the sequence for human TLE1 443-770 was generated according Laurence Pearl's laboratory procedure.[3] Briefly, the C-terminal region of human TLE1 (GenBank accession number M99435) encoding amino acids 443–770 was PCR amplified and cloned into pFastBacHTb (GIBCO) in-frame with the N-terminal His6 tag sequence. Starting from this construct we generated recombinant baculovirus using the Bac-to-Bac® Baculovirus Expression System (Thermo Fisher Scientific). For protein production, Sf9 insect cells were grown in sf-900 II SFM media (Thermo Fisher Scientific) to a cell density of around 2×10^{6} cells per milliliter and infected with 20 µL of virus per 10^{7}cells. Infected cell cultures were harvested 3 days post-infection.

Cell pellets were resuspended in 6 volumes of lysis buffer (50 mM Tris pH 7.5, 500 mM NaCl, 1 mM MgCl) containing 1× complete EDTA-free protease inhibitors and 25 U/mL benzonase nuclease. The resuspended cells were lysed by sonication. Following

centrifugation, the supernatant was purified over 2x HiTrap talon crude columns (Cobalt-IMAC, GE). Columns were washed with 10mM Imidazole and then the protein was eluted in 250mM Imidazole.

The Talon eluate was incubated overnight at 4 °C with TEV protease to cleave the 6xHis tag and then re-loaded on talon column: the unbound wash contains the cleaved hTLE1 443-770.

The protein was subsequently purified over a Superdex 75 16/60 column that was equilibrated in 50 mM Hepes pH 7.5 + 250 mM naCl + 1 mM TCEP.

Selected fractions were pooled, concentrated to 8 mg/mL and stored at -80 °C. The protein was stable in these conditions for up to three months.

Protein concentration was measured using an ND-1000 UV spectrophotometer (Nanodrop Technologies Inc., DE., USA).

Crystals of apo-TLE1 were produced by microbatch (under oil). For the plate using 6.2 mg/mL TLE1 construct, the drops contained 1:1 precipitant solution (PEG 8000 (22%), 100mM sodium cacodylate, 100 mM $Ca(OAc)_2$): protein solution (6.2 mg/mL TLE1 batch 2C, 25 mM Tris pH 8.0, 140 mM NaCl, 0.5 mM EDTA, 5 mM DTT). For the plate using 8.2 mg/mL TLE1 construct, the drops contained 1:1 precipitant solution (PEG 8000 (16%), 100mM sodium cacodylate, 100 mM $Ca(OAc)_2$): protein solution (8.2 mg/mL TLE1 batch 2C, 25 mM Tris pH 8.0, 140 mM NaCl, 0.5 mM EDTA, 5 mM DTT). The crystals were grown over 1-2 days at 20 °C. Some crystals were transferred to hanging drop (vapour diffusion) plates with the wells containing precipitant solution (PEG 8000 (22%), 100mM Na cacodylate, 100 mM $Ca(OAc)_2$) and the drops containing the same precipitant solution supplemented with 1.0 or 2.5 mM peptide and a resultant final DMSO concentration of 1.0% or 2.5%, respectively. These crystals were soaked for around 5 h at 20 °C. Apo or peptide-soaked crystals were harvested, briefly transferred to cryprotectant (either paratone-N or a buffer containing ethylene glycol (40%), PEG 8000 (50%), 100 mM sodium cacodylate, 100 mM $Ca(OAc)_2$) and flash frozen in liquid nitrogen.

X-Ray data were collected at the Diamond Light Source, Oxford, UK on beamline I04. Crystals belonged to the space group $P2_1$ and diffracted to between 1.6 and 2.9 Å. Data were integrated and merged using MOSFLM[4a,b] and AIMLESS.[4c] The structures were solved by molecular replacement using PHASER,[5] with either 2CE9 or our refined apo structure as the molecular replacement model with ligands and water removed. The structures were manually rebuilt in COOT[6] and refined with BUSTER[7] in iterative cycles.

Ligand restraints for the constrained peptide were generated with Grade[8] and Mogul.[9] The quality of the structures was assessed with MOLPROBITY.[10]

Peptides

Linear, unmodified peptides FWRPW and MWRPW were purchased from Cambridge Peptides as lyophilized powder. The peptides were then reconstituted in 1:1 mixture of DMSO and H_2O as 10 mM stocks and stored at -80 °C.

The concentration in solution was determined by UV spectrophotometry and confirmed by NMR.

LCMS spectra generated at Cambrige Peptide of these peptides are included in the LCMS section of the supporting information.

Thermal Shift Assay

Thermal Shift Assay (TSA) against 6 μM humanTLE1 443-770 was carried out using a C1000 Thermal Cycler CFX Real Time Detection System (Bio-Rad, Hemel Hempstead, UK). The assay buffer consisted of 25 mM Tris pH7.5, 125 mM NaCl, 1 mM DTT and 0.0002%Tween20 + 3x SYPRO® Orange™ protein gel stain (Sigma-Aldrich). All experiments were performed in a black frame, white 384-well FrameStar skirted PCR-plate (4titude, Surrey, UK). The plate was heated from 10 °C to 95 °C with a heating range of 0.5 °/min. For each experiment, the data range of the protein unfolding transition was established and the melting temperature (Tm) calculated using Vortex scripting (Vortex software, Dotmatics, Hertfordshire, UK, www.dotmatics.com).

All the peptides were dissolved in 1:1 mixture of H_2O and DMSO to obtain a final 10 mM stock solution. The peptides were then titrated against a fixed concentration of TLE1 and the change in the melting temperature caused by ligand binding was calculated by subtracting Tm of the protein alone from each melting temperature obtained in the presence of a ligand (ΔTm). All measurements were carried out in triplicate.

ITC

ITC experiments were carried out at 25 °C in a MicroCal iTC200 (GE healthcare) in high gain feedback mode, with an injection volume of 1.5 μL, a time spacing of 180 s between injections, a stirrer speed of 700rpm, a filter period of 5 s and a reference power of 6 μcal/s.

The purified protein was placed in the sample cell and the peptides were injected in the cell through the titration syringe. The final ITC samples were prepared in buffer containing 50

mM Hepes pH7.5, 250 mM NaCl, 1 mM TCEP and 0.05% Tween 20. Peptides were prepared as 10 mM stock solutions in 1:1 mixture of H_2O and DMSO and then diluted in buffer to the final desired concentrations. The DMSO content of the protein solution was then adjusted according to the peptide samples in order to have a perfect buffer match. The reference cell contained degassed water.

A mock titration (peptide injections into buffer) was performed for every ITC experiment to verify the generated signal was not due to peptide-buffer interactions or dilution effects.

Baseline assignment, peak integration and data fitting were accomplished by use of MicroCal PEAQ ITC Analysis software (Malvern).

Supplementary figure

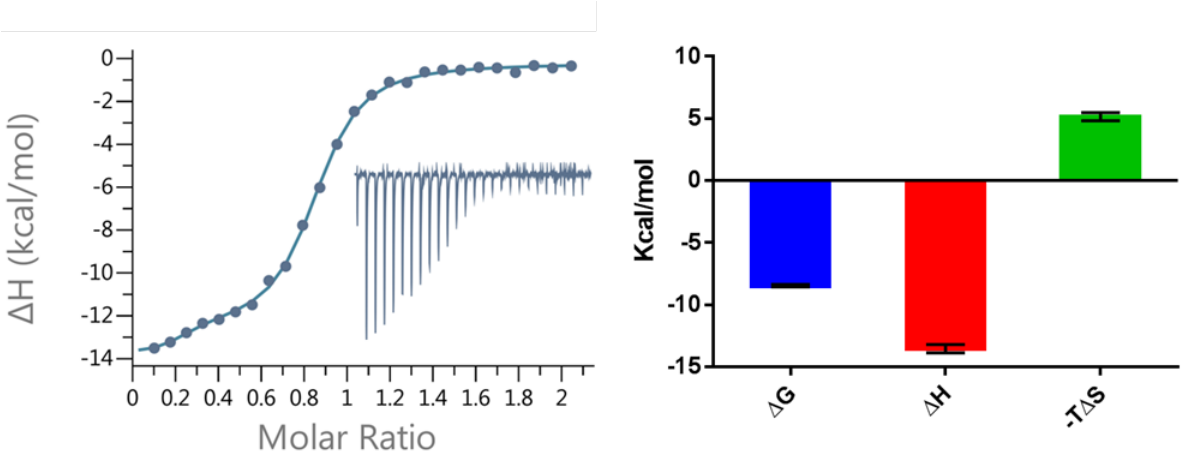

Figure S1. ITC measurement of MWRPW binding to TLE1. Experiments performed with TLE1 30 μM and MWRPW peptide 350μM. On the left, data fitting to a one-site independent binding model, integrated heats are shown in the inset. On the right, histograms showing ΔG, ΔH, and –TΔS, histograms represent averaged values, error bars denote SD the thermodynamic values are also presented in Table S1. Error bars denote SD, n = 2.

Table S1. Kd and thermodynamic values determined in ITC for MWRPW-TLE1 binding experiments

Kd (nM)	N	ΔH (Kcal/mol)	ΔS (Kcal/mol/T)	-TΔS (Kcal/mol)	ΔG (Kcal/mol)
704 ± 14	0.81 ± 0.017	-13.54 ± 0.325	-0.017 ± 0.001	5.14 ± 0.32	- 8.4 ± 0.009

.

Characterisation of compounds

Hydrogenation Product (4)

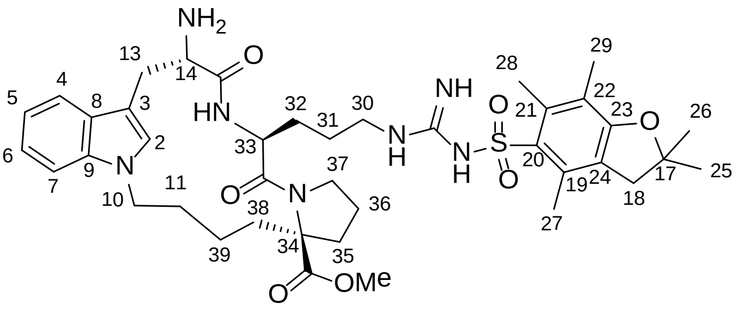

To a solution of metathesis product **(12)** (588 mg, 0.650 mmol) in MeOH (60 mL) was added $Pd(OH)_2/C$ (20 wt. % loading (dry basis), ≤50% water, 904 mg, 0.650 mmol) and the mixture stirred under balloon-pressure hydrogen at 20 °C for 22 h. The catalyst was filtered off (celite) and the filtrate concentrated *in vacuo*. The reaction was repeated with another 588 mg starting material, this time stirring for 20 h. Crude materials were combined and purified by flash column chromatography (0-100% EtOAc in cyclohexane then 0-15% MeOH in EtOAc) to yield the title compound as a white solid (761 mg, 0.98 mmol, 76%).

m.p.: 170-173 °C; R_f = 0.4 (2:3:8 water / propan-2-ol / EtOAc); ^{1}H NMR (500 MHz, MeOH) δ 0.84-0.97 (1H, m C$\underline{H}_2$-39a), 1.19-1.26 (1H, m, C$\underline{H}_2$-39b), 1.45 (3H, s, C$\underline{H}_3$-25/C$\underline{H}_3$-26), 1.45 (3H, s, C$\underline{H}_3$-26/C$\underline{H}_3$-25), 1.57-1.70 (5H, m, C$\underline{H}_2$-11a, C$\underline{H}_2$-31, C$\underline{H}_2$-32a, C$\underline{H}_2$-38a), 1.74-1.79 (1H, m, C$\underline{H}_2$-32b), 1.82-1.95 (3H, m, C$\underline{H}_2$-11b, C$\underline{H}_2$-36), 2.04-2.12 (2H, m, C$\underline{H}_2$-35), 2.08 (3H, s, C$\underline{H}_3$-27/C$\underline{H}_3$-28/C$\underline{H}_3$-29), 2.52 (3H, s, C$\underline{H}_3$-27/C$\underline{H}_3$-28/C$\underline{H}_3$-29), 2.59 (3H, s, C$\underline{H}_3$-27/C$\underline{H}_3$-28/C$\underline{H}_3$-29), 2.65-2.72 (1H, m, C$\underline{H}_2$-38b), 2.99 (2H, s, C$\underline{H}_2$-18), 3.17-3.26 (4H, m, C$\underline{H}_2$-13, C$\underline{H}_2$-30), 3.64 (3H, s, OC$\underline{H}_3$), 3.71-3.74 (2H, m, C$\underline{H}_2$-37), 3.91 (1H, dd, J = 4, 7 Hz, H-14), 4.09-4.25 (2H, m, C$\underline{H}_2$-10), 4.63-4.65 (1H, m, H-33), 6.96 (1H, s, H-2), 7.05 (1H, dd, J = 8, 8 Hz, H-5), 7.14 (1H, J = 7, 7 Hz, H-6), 7.31 (1H, d, J = 8 Hz, H-7), 7.63 (1H, d, J = 8 Hz, H-4); ^{13}C NMR (126 MHz, MeOH) δ 11.1 (C-27/C-28/C-29), 17.0 (C-27/C-28/C-29), 18.2 (C-27/C-28/C-29), 19.2 (C-39), 23.2 (C-36), 24.8 (C-31), 27.3 (C-25, C-26), 28.4 (C-13, C-32), 29.1 (C-11), 32.3 (C-38), 35.9 (C-35), 40.2 (C-30), 42.6 (C-18), 43.7 (C-10), 49.1 (C-37), 50.3 (C-33), 51.5 (O$\underline{C}H_3$), 55.1 (C-14), 69.0 (C-34), 86.3 (C-17), 106.6 (C-3), 109.1 (C-7), 117.1 (Pbf-Cq), 118.2 (C-4), 118.7 (C-5), 121.0 (C-6), 124.6 (Pbf-Cq), 128.4 (C-8), 128.7 (C-2), 132.1 (Pbf-Cq), 133.0 (Pbf-Cq), 135.8 (C-9), 138.0 (Pbf-Cq), 156.7 ($\underline{C}N_3$), 158.5 (C-23), 170.6 (N$\underline{C}$OC), 172.0 (N$\underline{C}$OC), 174.4 ($\underline{C}O_2CH_3$); $[\alpha]_D^{22}$ = -48 (c 1, $CHCl_3$); IR (solid) 3339, 2923, 1736, 1546, 1099, 1085, 566; LCMS (Fast4min) t_r = 2.68 min, m/z 778 $[M + H]^+$; purity

(AUC) > 95%; HRMS (ESI) *m/z* calcd for $C_{40}H_{56}N_7O_7S$ $[M + H]^+$ 778.3956, found $[M + H]^+$ 778.3945.

(*R*)-Methyl 2-allyl-1-((*S*)-2-((*S*)-3-(1-allyl-1*H*-indol-3-yl)-2-(((benzyloxy)carbonyl) amino) propanamido)-5-(3-((2,2,4,6,7-pentamethyl-2,3-dihydrobenzofuran-5-yl) sulfonyl)guanidino) pentanoyl)pyrrolidine-2-carboxylate (5)

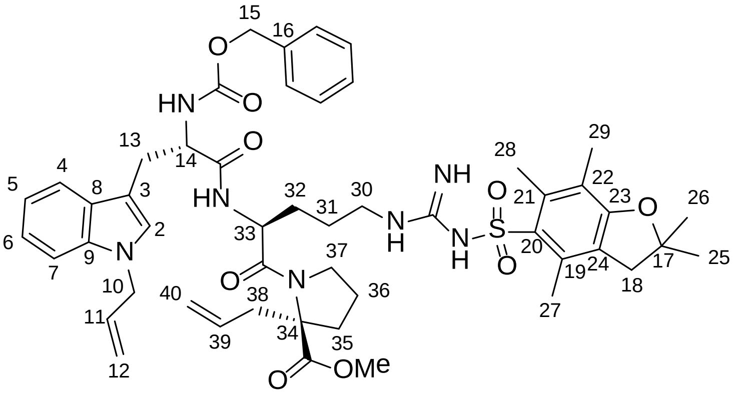

To a solution of (*S*)-3-(1-allyl-1*H*-indol-3-yl)-2-(benzyloxycarbonylamino)propanoic acid **(7)** (131 mg, 0.35 mmol) in DMF (6 mL) were added DIPEA (0.09 mL, 0.50 mmol) and HATU (130 mg, 0.342 mmol), and the mixture was stirred at room temperature for 3.5 h. A separate solution of (*R*)-methyl 1-((*S*)-2-((((9H-fluoren-9-yl)methoxy)carbonyl)amino)-5-(3-((2,2,4,6,7-pentamethyl-2,3-dihydrobenzofuran-5-yl) sulfonyl)guanidino)pentanoyl)-2-allyl-pyrrolidine-2-carboxylate **(9)** (114 mg, 0.14 mmol) in EtOH (4 mL) was cooled to 0 °C and treated with NaOEt (15 mg, 0.22 mmol). After stirring for 2.5 h, HCl (1.25 M in MeOH, 0.29 mL, 0.36 mmol) was added and the solvent removed *in vacuo*. To the residue was added the first reaction mixture at 0 °C, and the mixture stirred for 30 min. EtOAc (25 mL) and brine (25 mL) were added and the organic layer was collected, dried ($MgSO_4$) and concentrated *in vacuo*. The crude material was purified by flash column chromatography (25-100% EtOAc in cyclohexane) to yield the title compound as a cream solid (94 mg, 0.10 mmol, 70%).

m.p.: 195-199 °C; R_f = 0.3 (EtOAc); ^{1}H NMR (500 MHz, $CDCl_3$) δ 1.40-1.50 (2H, br m, $C\underline{H}_2$-31/$C\underline{H}_2$-32), 1.45 (6H, s, $C\underline{H}_3$-25, $C\underline{H}_3$-26), 1.52-1.58 (1H, br m, $C\underline{H}_2$-32/$C\underline{H}_2$-31), 1.78-1.80 (1H, br m, $C\underline{H}_2$-32/$C\underline{H}_2$-31), 1.93-2.04 (3H, br m, $C\underline{H}_2$-35a, $C\underline{H}_2$-36), 2.06-2.12 (1H, br m, $C\underline{H}_2$-35b), 2.09 (3H, s, $C\underline{H}_3$-27/$C\underline{H}_3$-28/$C\underline{H}_3$-29), 2.52 (3H, s, $C\underline{H}_3$-27/$C\underline{H}_3$-28/$C\underline{H}_3$-29), 2.59 (3H, s, $C\underline{H}_3$-27/$C\underline{H}_3$-28/$C\underline{H}_3$-29), 2.64 (1H, dd, J = 8, 14 Hz, $C\underline{H}_2$-38a), 2.94 (2H, s, $C\underline{H}_2$-18), 3.00-3.07 (2H, m, $C\underline{H}_2$-30a, $C\underline{H}_2$-38b), 3.16-3.23 (3H, m, $C\underline{H}_2$-13, $C\underline{H}_2$-30b), 3.52-3.57 (1H, m, $C\underline{H}_2$-37a), 3.60-3.63 (1H, m, $C\underline{H}_2$-37b), 3.67 (3H, s, $OC\underline{H}_3$), 4.47-4.51 (1H, m, H-14),

4.61-4.66 (3H, m, $C\underline{H}_2$-10, H-33), 4.98-5.15 (6H, m, $C\underline{H}_2$-12, $C\underline{H}_2$-15, $C\underline{H}_2$-40), 5.53 (1H, br s, $N\underline{H}$), 5.61-5.69 (1H, m, H-39), 5.85-5.97 (2H, m, H-11, $N\underline{H}$), 6.13 (2H, br s, $N\underline{H}_2$), 6.93 (1H, s, H-2), 7.05 (1H, dd, J = 7, 8 Hz, H-5), 7.18 (1H, dd, J = 7, 7 Hz, H-6), 7.25-7.31 (6H, m, H-7, 5 × Ar-$C\underline{H}$), 7.58 (1H, d, J = 8 Hz, H-4); ^{13}C NMR (126 MHz, $CDCl_3$) δ 12.5 (C-27/C-28/C-29), 17.9 (C-27/C-28/C-29), 19.3 (C-27/C-28/C-29), 23.7 (C-36), 23.9 (C-31/C-32), 27.9 (C-13), 28.6 (C-25, C-26), 28.7 (C-32/C-31), 35.1 (C-35), 37.9 (C-38), 40.8 (C-30), 43.2 (C-18), 48.6 (C-37), 48.7 (C-10), 50.7 (C-33), 52.6 ($O\underline{C}H_3$), 55.7 (C-14), 67.1 (C-15), 68.9 (C-34), 86.3 (C-17), 108.8 (C-3), 109.8 (C-7), 117.3 (C-12/C-40), 117.4 (Pbf-Cq), 118.9 (C-4), 119.3 (C-5), 119.5 (C-40/C-12), 121.9 (C-6), 124.6 (Pbf-Cq), 127.0 (C-2), 127.9 (2 × Ar-$\underline{C}H$), 128.0 (C-8), 128.2 (Ar-$\underline{C}H$), 128.5 (2 × Ar-$\underline{C}H$), 132.3 (Pbf-Cq), 132.9 (C-39), 133.0 (Pbf-Cq), 133.4 (C-11), 136.0 (C-16), 136.4 (C-9), 138.4 (Pbf-Cq), 156.1 ($N\underline{C}O_2$/$\underline{C}N_3$), 156.3 ($\underline{C}N_3$/$N\underline{C}O_2$), 158.7 (C-23), 169.4 ($N\underline{C}OC$), 171.7 ($N\underline{C}OC$), 174.1 ($\underline{C}O_2CH_3$); $[\alpha]_D^{22}$ = +17 (c 1, $CHCl_3$); IR (solid) 2923, 1737 (C=O), 1621, 1548, 1089, 728; LCMS (Fast4min) t_r = 3.40 min, m/z 938 $[M + H]^+$; purity (AUC) > 95%; HRMS (ESI) m/z calcd for $C_{50}H_{63}N_7O_9S$ $[M + H]^+$ 938.4481, found $[M + H]^+$ 938.4447.

(*R*)-Methyl 2-allylpyrrolidine-2-carboxylate, HCl salt (6-HCl), (*R*)-2-Allylpyrrolidine-2-carboxylic acid, HCl salt (37) and (*R*)-Methyl 2-allylpyrrolidine-2-carboxylate (6)

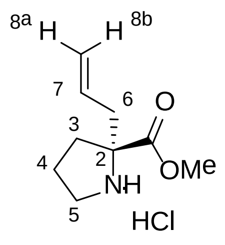

6·HCl

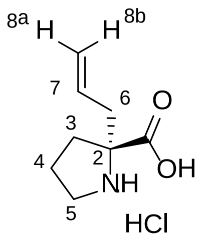

37

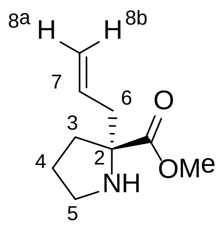

6

To a solution of (3*R*,7a*R*)-7a-allyl-3-(trichloromethyl)tetrahydropyrrolo[1,2-*c*]oxazol-1(3*H*)-one **(34)** (3.45 g, 12.1 mmol) in MeOH (20 mL) was added hydrogen chloride (1.25 M in MeOH, 100 mL). The mixture was heated to reflux for 4 h, cooled to room temperature, and most of the solvent removed *in vacuo*. The crude material was purified by flash column chromatography (gradient elution 2.5-10% MeOH in CH_2Cl_2) to yield three products: (*R*)-methyl 2-allylpyrrolidine-2-carboxylate, HCl salt **(6-HCl)** (1.25 g, 6.08 mmol, 50%) as a brown solid which possessed spectroscopic data that were consistent with those in the literature,[11] (*R*)-2-allylpyrrolidine-2-carboxylic acid, HCl salt **(37)** (1.05 g, 5.49 mmol, 45%) as a brown oil and (*R*)-methyl 2-allylpyrrolidine-2-carboxylate **(6)** (60 mg, 0.34 mmol, 3%) as a brown oil.

(*R*)-Methyl 2-allylpyrrolidine-2-carboxylate, HCl salt (6-HCl)

m.p: 119-124 °C, lit[12] 122.5-123 °C; R_f = 0.3 (2:3:8 water / propan-2-ol / EtOAc); ^{1}H NMR (500 MHz, MeOD) δ 2.00-2.15 (3H, br m, C$\underline{H}_2$-4, C$\underline{H}_2$-3a), 2.47-2.52 (1H, br m, C$\underline{H}_2$-3b), 2.70 (1H, dd, *J* = 7, 14 Hz, H-6a), 2.93 (1H, dd, *J* = 7, 14 Hz, H-6b), 3.45 (2H, br dd, *J* = 7, 7 Hz, C$\underline{H}_2$-5), 3.87 (3H, s, OC$\underline{H}_3$), 5.28 (1H, d, *J* = 10, H-8a), 5.33 (1H, d, *J* = 17, H-8b), 5.74 (1H, dddd, *J* = 7, 7, 10, 17 Hz, H-7); ^{13}C NMR (126 MHz, MeOD) δ 22.2 (C-4), 34.0 (C-3), 38.9 (C-6), 45.8 (C-5), 54.2 (O$\underline{C}$H$_3$), 72.7 (C-2), 121.8 (C-8), 129.6 (C-7), 171.7 ($\underline{C}$OOCH$_3$); $[\alpha]_D^{22}$ = -52 (*c* 2, MeOH), lit[13] $[\alpha]_D$ = +74, (*c* 2, CH_2Cl_2); LCMS (Fast4min) t_r = 0.78 min, *m/z* 170 [M + H]$^+$, purity (AUC) > 95%; HRMS (ESI) *m/z* calcd for $C_9H_{15}NNaO_2$ [M + Na]$^+$ 192.0995, found [M + Na]$^+$ 192.0991.

(*R*)-2-Allylpyrrolidine-2-carboxylic acid, HCl salt (37)

R_f = 0.1 (2:3:8 water / propan-2-ol / EtOAc); ^{1}H NMR (500 MHz, MeOD) δ 2.01-2.17 (3H, br m, C$\underline{H}_2$-4, C$\underline{H}_2$-3a), 2.48-2.51 (1H, br m, C$\underline{H}_2$-3b), 2.63-2.75 (1H, br m, H-6a), 2.94 (1H, dd, *J* = 7, 15 Hz, H-6b), 3.43-3.45 (2H, br m, C$\underline{H}_2$-5), 5.29 (1H, d, *J* = 10, H-8a), 5.36 (1H, d, *J* = 17, H-8b), 5.70-5.83 (1H, m, H-7); ^{13}C NMR (126 MHz, MeOD) δ 22.5 (C-4), 34.4 (C-3), 39.0 (C-6), 45.8 (C-5), 73.2 (C-2), 121.3 (C-8), 130.2 (C-7); $[\alpha]_D$ not obtained as material not sufficiently pure; LCMS (Fast4min) t_r = 0.52 min, *m/z* 156 [M + H]$^+$, purity (AUC) = 59% (41% ester **6-HCl**); HRMS (ESI) *m/z* calcd for $C_8H_{14}NO_2$ [M + H]$^+$ 156.1019, found [M + H]$^+$ 156.1022.

(*R*)-Methyl 2-allylpyrrolidine-2-carboxylate (6)

R_f = 0.3 (2:3:8 water / propan-2-ol / EtOAc); ^{1}H NMR (500 MHz, MeOD) δ 1.74-1.92 (3H, m, C$\underline{H}_2$-4, C$\underline{H}_2$-3a), 2.22-2.28 (1H, m, C$\underline{H}_2$-3b), 2.44 (1H, dd, *J* = 7, 14 Hz, H-6a), 2.63 (1H, dd, *J* = 7, 14 Hz, H-6b), 3.04 (2H, dd, *J* = 7, 7 Hz, C$\underline{H}_2$-5), 3.74 (3H, s, OC$\underline{H}_3$), 5.09-5.15 (2H, m, C$\underline{H}_2$-8), 5.75 (1H, dddd, *J* = 7, 8, 15, 17 Hz, H-7); ^{13}C NMR (126 MHz, MeOD) δ 23.8 (C-4), 34.7 (C-3), 42.3 (C-6), 45.5 (C-5), 51.6 (O$\underline{C}$H$_3$), 69.8 (C-2), 118.0 (C-8), 132.7 (C-7), 175.0 ($\underline{C}$OOCH$_3$); $[\alpha]_D^{22}$ = -66 (*c* 2, MeOH), IR (oil) 3368 (N-H), 1737 (C=O), 1640, 1435, 1239, 930 (C=C); LCMS (Fast4min) t_r = 0.63 min, *m/z* 170 [M + H]$^+$, purity (AUC) > 95%; HRMS (ESI) *m/z* calcd for $C_9H_{15}NNaO_2$ [M + Na]$^+$ 192.0995, found [M + Na]$^+$ 192.0992.

(*S*)-3-(1-Allyl-1*H*-indol-3-yl)-2-(benzyloxycarbonylamino)propanoic acid (7)

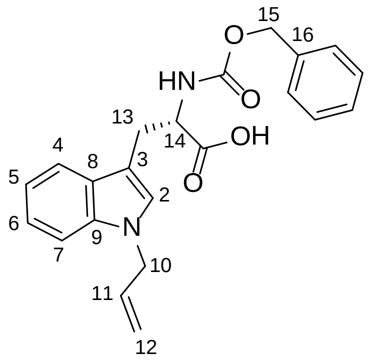

To a suspension of L-tryptophan (204 mg, 1.00 mmol) in DMF (5 mL) was added NaH (60% in mineral oil, 120 mg, 3.00 mmol) and the mixture stirred at room temperature for 30 min. The suspension was cooled to 0 °C and a solution of allyl bromide (87 µL, 1.0 mmol) in DMF (5 mL) was added dropwise over 1 h. After stirring at 0 °C for 2 h, water (10 mL), Na_2CO_3 (212 mg, 2.00 mmol) and benzyl chloroformate (0.14 mL, 1.0 mmol) were added at 0 °C. After stirring at 0 °C for 3 h, more benzyl chloroformate (72 µL, 0.50 mmol) was added and after a final 1 h at 0 °C the reaction mixture was diluted with EtOAc (25 mL). The layers were separated, the organic layer was extracted with 0.5 M NaOH (aq, 100 mL) and the combined aqueous layers were acidified to pH 1 with 5.82 M HCl (aq, 10 mL) and extracted with EtOAc (50 mL). The resultant organic layer was dried ($MgSO_4$) and concentrated *in vacuo*. The crude material was purified by flash column chromatography (gradient elution 9:1 EtOAc / cyclohexane → EtOAc → 9:1 EtOAc / MeOH) to yield the title compound (107 mg, 0.283 mmol, 28%) as a yellow solid.

m.p: 75-76 °C; R_f = 0.4 (EtOAc); ^{1}H NMR (500 MHz, 1:1 MeOD / $CDCl_3$) δ 3.17 (1H, dd, J = 7, 15 Hz, C$\underline{H}_2$-13a), 3.34 (1H, dd, J = 4, 15 Hz, C$\underline{H}_2$-13b), 4.49-4.51 (1H, br m, H-14), 4.55-4.56 (2H, br m, C$\underline{H}_2$-10), 4.90-5.07 (4H, m, C$\underline{H}_2$-12, C$\underline{H}_2$-15), 5.81-5.89 (1H, m, H-11), 6.88 (1H, s, H-2), 6.99 (1H, dd, J = 8, 8 Hz, H-5), 7.11 (1H, dd, J = 8, 8 Hz, H-6), 7.20-7.25 (6H, m, 6 × Ar-C$\underline{H}$), 7.54 (1H, d, J = 8 Hz, H-4); ^{13}C NMR (126 MHz, 1:1 MeOD / $CDCl_3$) δ 27.6 (C-13), 48.4 (C-10), 55.5 (C-14), 66.6 (C-15), 109.5 (C-7), 109.7 (C-3), 115.7 (C-12), 118.8 (C-4), 119.0 (C-5), 121.4 (C-6), 126.7 (C-2), 127.7 (2 × Ar-$\underline{C}$H), 127.9 (Ar-$\underline{C}$H), 128.3 (2 × Ar-$\underline{C}$H), 128.4 (C-8), 133.5 (C-11), 136.3 (C-9/C-16), 136.3 (C-16/C-9), 156.6 (N$\underline{C}$O), 176.7 ($\underline{C}O_2$H); $[\alpha]_D^{22}$ = -11 (c 0.5, $CHCl_3$); IR (solid) 3309 (O-H), 1683 (C=O), 1552 (N-H), 1330 (O-H), 990 (C=C), 923 (C=C); LCMS (Fast4min) t_r = 3.04 min, m/z 379 $[M + H]^+$, purity (AUC) = 87%; HRMS (ESI) m/z calcd for $C_{22}H_{22}N_2NaO_4$ $[M + Na]^+$ 401.1472, found $[M + Na]^+$ 401.1474.

(*S*)-(9*H*-Fluoren-9-yl)methyl 2-oxo-1-(*N*-(2,2,4,6,7-pentamethyl-2,3-dihydrobenzofuran-5-ylsulfonyl)carbamimidoyl)piperidin-3-ylcarbamate (8)

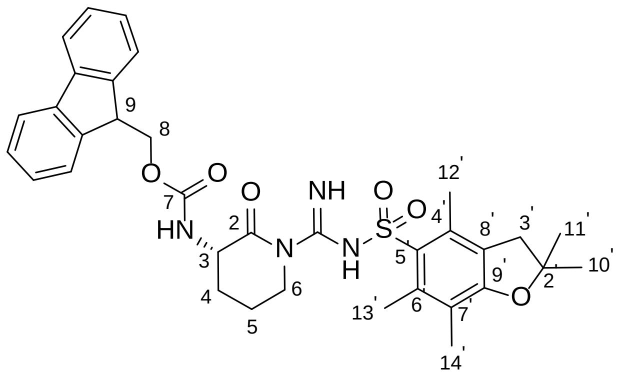

At room temperature, to (*R*)-methyl 2-allylpyrrolidine-2-carboxylate, HCl salt **(6-HCl)** (23 mg, 0.11 mmol) were added (*S*)-2-(((9*H*-fluoren-9-yl)methoxy)carbonylamino)-5-(3-(2,2,4,6,7-pentamethyl-2,3-dihydrobenzofuran-5-ylsulfonyl)guanidine)pentanoic acid (88 mg, 0.14 mmol), MeCN (2 mL), DIPEA (60 µL, 0.34 mmol) and HATU (57 mg, 0.15 mmol). After stirring for 2.5 h, the solvent was removed *in vacuo* and the crude material purified by flash column chromatography (1:1 cyclohexane / EtOAc) to yield the title compound as a white solid (40 mg, 0.063 mmol, 57%).

m.p: 102-107 °C; R_f = 0.3 (1:1 cyclohexane / EtOAc); ^{1}H NMR (500 MHz, $CDCl_3$) δ 1.48 (6H, s, C<u>H</u>$_3$-10', C<u>H</u>$_3$-11'), 1.53-1.64 (1H, br m, C<u>H</u>$_2$-4a), 1.84-1.95 (2H, br m, C<u>H</u>$_2$-5), 2.13 (3H, s, C<u>H</u>$_3$-14'), 2.45-2.52 (1H, br m, C<u>H</u>$_2$-4b), 2.55 (3H, s, C<u>H</u>$_3$-12'), 2.60 (3H, s, C<u>H</u>$_3$-13'), 2.98 (2H, s, C<u>H</u>$_2$-3'), 3.38-3.45 (1H, br m, C<u>H</u>$_2$-6a), 4.24 (1H, t, *J* = 7 Hz, H-9), 4.39-4.60 (4H, br m, C<u>H</u>$_2$-6b, H-3, C<u>H</u>$_2$-8), 5.59 (1H, br s, N<u>H</u>), 7.32 (2H, dd, *J* = 7, 8 Hz, 2 × Ar-C<u>H</u>), 7.41 (2H, dd, *J* = 7, 7 Hz, 2 × Ar-C<u>H</u>), 7.60-7.62 (2H, br m, 2 × Ar-C<u>H</u>), 7.77 (2H, br d, *J* = 7 Hz, 2 × Ar-C<u>H</u>), 7.91 (1H, br s, N<u>H</u>), 9.41 (1H, br s, N<u>H</u>); ^{13}C NMR (126 MHz, $CDCl_3$) δ 12.4 (C-14'), 18.0 (C-13'), 19.2 (C-12'), 19.6 (C-5), 25.2 (C-4), 28.6 (C-10', C-11'), 42.0 (C-6), 43.2 (C-3'), 47.2 (C-9), 52.7 (C-3), 67.2 (C-8), 86.7 (C-2'), 117.8 (C-6'/C-7'), 120.0 (2 × Ar-<u>C</u>H), 124.9 (C-4'), 125.1 (2 × Ar-<u>C</u>H), 127.1 (2 × Ar-<u>C</u>H), 127.8 (2 × Ar-<u>C</u>H), 131.7 (C-5'/C-8'), 132.8 (C-8'/C-5'), 138.9 (C-7'/C-6'), 141.3 (Fmoc-Cq), 143.7 (C-7), 143.8 (Fmoc-Cq), 153.8 (Fmoc-Cq), 156.0 (Fmoc-Cq), 159.3 (C-9'), 175.7 (C-2); $[\alpha]_D^{22}$ = +13 (*c* 0.5, $CHCl_3$); IR (solid) 1697 (C=O), 1616, 1523, 1262, 759; LCMS (Fast4minLipophilic) t_r = 1.88 min, *m/z* 631 $[M + H]^+$, purity (AUC) = 86%; HRMS (ESI) *m/z* calcd for $C_{34}H_{39}N_4O_6S$ $[M + H]^+$ 631.2585, found $[M + H]^+$ 631.2580.

(*R*)-Methyl 1-((*S*)-2-(((9H-fluoren-9-yl)methoxy)carbonylamino)-5-(3-(2,2,4,6,7-pentamethyl-2,3-dihydrobenzofuran-5-ylsulfonyl)guanidino)pentanoyl)-2-allylpyrrolidine-2-carboxylate (9)

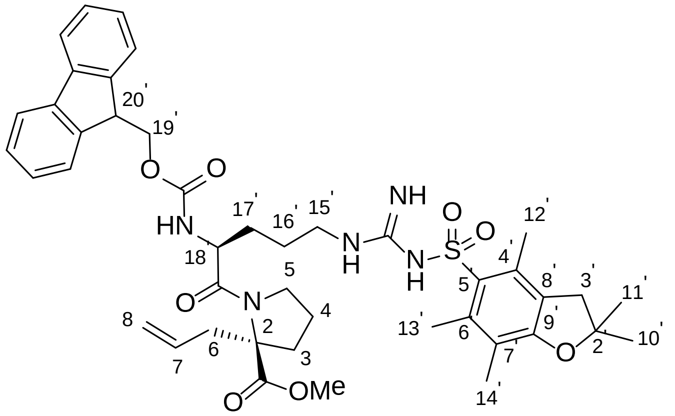

To a solution of (*R*)-methyl 2-allylpyrrolidine-2-carboxylate, HCl salt **(6-HCl)** (200 mg, 0.970 mmol) in MeCN (1.6 mL) was added DIPEA (0.51 mL, 2.9 mmol) at room temperature. After 30 min, HATU (738 mg, 1.94 mmol), (*S*)-2-(((9*H*-fluoren-9-yl)methoxy) carbonyl-amino)-5-(3-(2,2,4,6,7-pentamethyl-2,3-dihydrobenzofuran-5-ylsulfonyl)guanidine) pentanoic acid (1.26 g, 1.94 mmol) and MeCN (1 mL) were added and the mixture was heated to 50 °C. After 4.5 h, DIPEA (0.17 mL, 0.97 mmol), HATU (369 mg, 0.970 mmol), (*S*)-2-(((9*H*-fluoren-9-yl)methoxy)carbonylamino)-5-(3-(2,2,4,6,7-pentamethyl-2,3-dihydrobenzo-furan-5-ylsulfonyl)guanidine)pentanoic acid (630 mg, 0.970 mmol) and MeCN (0.3 mL) were added. After a final 1 h at 50 °C the reaction mixture was cooled to room temperature, diluted with MeCN, filtered and concentrated *in vacuo*. The crude material was purified by flash column chromatography (gradient elution 1:1 EtOAc / cyclohexane → EtOAc → 95:5 EtOAc / MeOH) to yield the title compound as a colourless oil (376 mg, 0.470 mmol, 48%).

R_f = 0.4 (EtOAc) ; ^{1}H NMR (500 MHz, $CDCl_3$) δ 1.44 (6H, s, C<u>H</u>$_3$-10’, C<u>H</u>$_3$-11’), 1.57-1.68 (3H, br m, C<u>H</u>$_2$-16’, C<u>H</u>$_2$-17’a), 1.80-2.13 (5H, br m, C<u>H</u>$_2$-3, C<u>H</u>$_2$-4, C<u>H</u>$_2$.17’b), 2.09 (3H, s, C<u>H</u>$_3$-14’), 2.53 (3H, s, C<u>H</u>$_3$-12’/ C<u>H</u>$_3$-13’), 2.60 (3H, s, C<u>H</u>$_3$-13’/ C<u>H</u>$_3$-12’), 2.61-2.66 (1H, m, C<u>H</u>$_2$-6b), 2.93 (2H, s, C<u>H</u>$_2$-3’), 3.08-3.12 (1H, m, C<u>H</u>$_2$-6a), 3.18-3.29 (2H, br m, C<u>H</u>$_2$-15’), 3.69 (3H, s, OC<u>H</u>$_3$), 3.58-3.72 (2H, m, C<u>H</u>$_2$-5), 4.17 (1H, br t, *J* = 7 Hz, H-20’), 4.36 (2H, br d, *J* = 7 Hz, C<u>H</u>$_2$-19’), 4.47 (1H, br dd, *J* = 7, 13 Hz, H-18’), 5.06-5.09 (2H, m, C<u>H</u>$_2$-8), 5.66 (1H, ddt, *J* = 8, 17, 8 Hz, H-7), 5.93-6.02 (1H, br m, N<u>H</u>), 6.14 (2H, s, 2 × N<u>H</u>), 7.26-7.30 (2H, m, Ar-C<u>H</u>), 7.38 (2H, dd, *J* = 7, 8 Hz, Ar-C<u>H</u>), 7.56 (2H, d, *J* = 7 Hz, Ar-C<u>H</u>), 7.75 (2H, d, *J* = 8 Hz, Ar-C<u>H</u>); ^{13}C NMR (126 MHz, $CDCl_3$) δ 12.4 (C-14’), 17.9 (C-12’/C-13’), 19.2 (C-13’/C-12’), 23.7 (C-4), 24.3 (C-16’), 28.6 (C-10’, C-11’), 29.1 (C-17’), 35.2 (C-3), 37.8 (C-6), 40.8 (C-15’), 43.2 (C-3’), 47.1 (C-20’), 48.7 (C-5), 52.1 (C-18’), 52.6 (O<u>C</u>H$_3$), 67.0 (C-19’), 68.9

(C-2), 86.3 (C-2'), 117.4 (C-6'), 119.5 (C-8), 119.9 (2 × Ar-C̲H), 124.5 (C-4'), 125.1 (2 × Ar-C̲H), 127.1 (2 × Ar-C̲H), 127.7 (2 × Ar-C̲H), 132.3 (C-7'/C-8'), 132.8 (C-7), 133.1 (C-8'/C-7'), 138.3 (C-5'), 141.3 (2 × Fmoc-Cq), 143.7 (Fmoc-Cq), 143.7 (Fmoc-Cq), 156.2 (C̲N_3/NC̲O_2), 156.3 (NC̲O_2/C̲N_3), 158.7 (C-9'), 170.1 (NC̲OC), 174.2 (C̲O_2CH_3); $[\alpha]_D^{22}$ = +7 (*c* 1, MeOH); IR (oil) 3422, 2972, 1711 (C=O), 1728 (C=O), 1547, 1247, 1091, 835, 740, 556; LCMS (Fast4min) t_r = 3.42 min, *m/z* 800 $[M + H]^+$, purity (AUC) > 95%; HRMS (ESI) *m/z* calcd for $C_{43}H_{53}N_5NaO_8S$ $[M + Na]^+$ 822.3507, found $[M + Na]^+$ 822.3508.

(*R*)-Methyl 2-allyl-1-((*S*)-2-amino-5-(3-(2,2,4,6,7-pentamethyl-2,3-dihydrobenzofuran-5-ylsulfonyl)guanidino)pentanoyl)pyrrolidine-2-carboxylate (10) and *N*-(*N*-(3-((3*R*,8a*R*)-8a-Allyl-1,4-dioxooctahydropyrrolo[1,2-*a*]pyrazin-3-yl)propyl)carbamimidoyl)-2,2,4,6,7-pentamethyl-2,3-dihydrobenzofuran-5-sulfonamide (11)

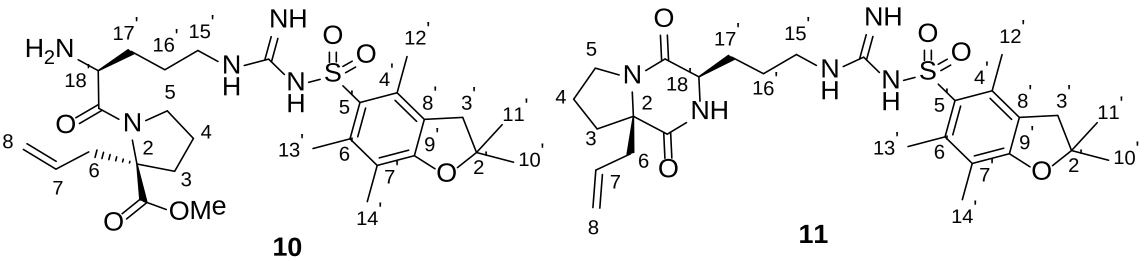

10 **11**

A solution of (*R*)-methyl 1-((*S*)-2-(((9*H*-fluoren-9-yl)methoxy)carbonylamino)-5-(3-(2,2,4,6,7-pentamethyl-2,3-dihydrobenzofuran-5-ylsulfonyl)guanidine)pentanoyl)-2-allylpyrrolidine-2-carboxylate **(9)** (20 mg, 0.025 mmol) in DMF (0.8 mL) was cooled to 0 °C and piperidine (2.5 µL, 0.025 mmol) added. After stirring at 0 °C for 2 h, the reaction mixture was diluted with water (20 mL) and extracted with Et_2O (5 × 10 mL). The combined organic layers were washed with brine (20 mL), dried ($MgSO_4$) and concentrated *in vacuo*. The crude material was purified by flash column chromatography (2:3:8 water : propan-2-ol : EtOAc) to yield trace amounts of two products: methyl 2-allyl-1-((*S*)-2-amino-5-(3-(2,2,4,6,7-pentamethyl-2,3-sihydrobenzo-furan-5-ylsulfonyl)guanidino)pentanoyl)pyrrolidine-2-carboxylate **(10)** and *N*-(*N*-(3-((3*R*,8a*R*)-8a-allyl-1,4-dioxooctahydropyrrolo[1,2-*a*]pyrazin-3-yl)propyl)carbamimido-yl)-2,2,4,6,7-pentamethyl-2,3-dihydrobenzofuran-5-sulfonamide **(11)**.

(*R*)-Methyl 2-allyl-1-((*S*)-2-amino-5-(3-(2,2,4,6,7-pentamethyl-2,3-dihydrobenzofuran-5-ylsulfonyl)guanidino)pentanoyl)pyrrolidine-2-carboxylate (10)

^{1}H NMR (500 MHz, $CDCl_3$) δ 1.47 (6H, s, CH̲$_3$-10', CH̲$_3$-11'), 1.60-2.18 (11H, br m, CH̲$_2$-4, CH̲$_2$-5, CH̲$_3$-12'/CH̲$_3$-13'/CH̲$_3$-14', CH̲$_2$-16', CH̲$_2$-17'), 2.53 (3H, s, CH̲$_3$-12'/CH̲$_3$-13'/CH̲$_3$-14'),

2.60 (3H, s, C$\underline{H}_3$-12'/C$\underline{H}_3$-13'/C$\underline{H}_3$-14'), 2.67 (1H, dd, J = 8, 14 Hz, C$\underline{H}_2$-6a), 2.96 (2H, s, C$\underline{H}_2$-3'), 3.11 (1H, dd, J = 7, 14 Hz, C$\underline{H}_2$-6b), 3.22 (2H, br t, J = 5 Hz, C$\underline{H}_2$-15'), 3.56-3.67 (2H, m, C$\underline{H}_2$-5a, H-18'), 3.71 (3H, s, OC$\underline{H}_3$), 3.73-3.81 (1H, br m, C$\underline{H}_2$-5b), 5.10-5.12 (2H, m, C$\underline{H}_2$-8), 5.67-5.75 (1H, m, H-7), 6.16 (1H, br s, N$\underline{H}$), 6.27 (2H, s, N$\underline{H}_2$); insufficient material to obtain ^{13}C NMR or $[\alpha]_D$; LCMS (Fast4minLipophilic) t_r = 1.63 min, *m/z* 578 [M + H]$^+$; HRMS (ESI) *m/z* calcd for $C_{28}H_{44}N_5O_6S$ [M + H]$^+$ 578.3007, found [M + H]$^+$ 578.3051.

_N_-(_N_-(3-((3_R_,8a_R_)-8a-Allyl-1,4-dioxooctahydropyrrolo[1,2-_a_]pyrazin-3-yl)propyl)carbamimidoyl)-2,2,4,6,7-pentamethyl-2,3-dihydrobenzofuran-5-sulfonamide (11)

m.p: 102 °C; R_f = 0.3 (EtOAc); ^{1}H NMR (500 MHz, $CDCl_3$) δ 1.47 (6H, s, C$\underline{H}_3$-10', C$\underline{H}_3$-11'), 1.55-1.71 (2H, br m, C$\underline{H}_2$-16'), 1.92-2.03 (4H, br m, C$\underline{H}_2$-4, C$\underline{H}_2$-17'), 2.11 (3H, s, C$\underline{H}_3$-13'), 2.11-2.16 (1H, m, C$\underline{H}_2$-4a), 2.21-2.25 (1H, m, C$\underline{H}_2$-4b), 2.45 (1H, dd, J = 8, 14 Hz, C$\underline{H}_2$-6a), 2.53 (3H, s, C$\underline{H}_3$-12'), 2.59 (3H, s, C$\underline{H}_3$-14'), 2.56-2.60 (1H, m, C$\underline{H}_2$-6b), 2.97 (2H, s, C$\underline{H}_2$-3'), 3.13-3.19 (1H, m, C$\underline{H}_2$-15'a), 3.22-3.28 (1H, m, C$\underline{H}_2$-15'b), 3.48-3.53 (1H, m, C$\underline{H}_2$-5a), 3.77-3.83 (1H, m, C$\underline{H}_2$-5b), 4.18 (1H, t, J = 4 Hz, H-18'), 5.18-5.21 (2H, m, C$\underline{H}_2$-8), 5.74-5.83 (1H, m, H-7), 6.21 (1H, br s, N$\underline{H}$), 7.34 (1H, br s, N$\underline{H}$); ^{13}C NMR (126 MHz, $CDCl_3$) δ 11.9 (C-13'), 17.4 (C-14'), 18.7 (C-12'), 19.6 (C-4/C-17'), 23.3 (C-16'), 27.3 (C-17'/C-4), 28.1 (C-10', C-11'), 33.6 (C-3), 40.2 (C-15'), 40.9 (C-6), 42.7 (C-3'), 44.4 (C-5), 54.4 (18'), 67.4 (C-2), 85.9 (C-2'), 117.0 (C-6'/C-7'), 117.0 (C-7'/C-6'), 120.3 (C-8), 124.2 (C-4'), 130.7 (C-7), 131.8 (C-8'), 137.9 (C-5'), 155.9 (C-9'/$\underline{C}N_3$), 158.3 ($\underline{C}N_3$/C-9'), 164.9 (N$\underline{C}$O), 171.6 (N$\underline{C}$O); insufficient material to obtain $[\alpha]_D$; IR (oil) 3336, 1634 (C=O), 1548 (C=O), 1089; LCMS (Fast4min) t_r = 2.83 min, *m/z* 546 [M + H]$^+$, purity (AUC) = 83%; HRMS (ESI) *m/z* calcd for $C_{27}H_{40}N_5O_5S$ [M + H]$^+$ 546.2745, found [M + H]$^+$ 546.2747.

Metathesis Product (12)

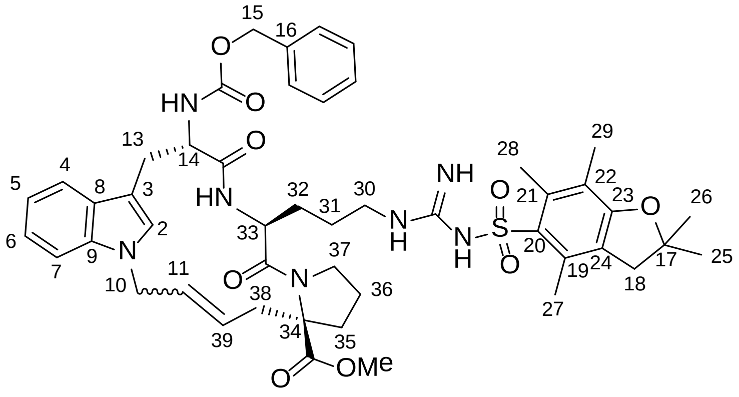

Method A (Small Scale)

To a solution of (*R*)-methyl 2-allyl-1-((*S*)-2-((*S*)-3-(1-allyl-1*H*-indol-3-yl)-2-(((benzyloxy)-carbonyl)amino) propanamido)-5-(3-((2,2,4,6,7-pentamethyl-2,3-dihydro-benzofuran-5-yl)-sulfonyl)guanidino)pentanoyl) pyrrolidine-2-carboxylate **(5)** (20 mg, 0.021 mmol) in CH_2Cl_2 (12 mL) were added 1,4-benzoquinone (0.9 mg, 0.01 mmol) and Grubbs catalyst, second generation (3.7 mg, 0.004 mmol), and the reaction mixture was heated to reflux for 1 h. The mixture was cooled to room temperature and the solvent removed *in vacuo*. The crude mixture was purified by flash column chromatography (25-100% EtOAc in cyclohexane) to yield the title compound as a yellow solid with a *trans*/*cis* ratio of approximately 4:1 (15 mg, 0.02 mmol, 78%). A small sample of the *trans* isomer was isolated by RP-HPLC using 15 min isocratic elution at 80:20 MeOH:water (both modified with 0.1% formic acid) at a flow rate of 5.0 mL/min as a pale yellow solid (3 mg).

Method B (Large Scale)

To a solution of (*R*)-methyl 2-allyl-1-((*S*)-2-((*S*)-3-(1-allyl-1*H*-indol-3-yl)-2-(((benzyloxy)-carbonyl)-amino) propanamido)-5-(3-((2,2,4,6,7-pentamethyl-2,3-dihydro-benzofuran-5-yl)-sulfonyl)guanidino)pentanoyl) pyrrolidine-2-carboxylate **(5)** (1.52 g, 1.62 mmol) in CH_2Cl_2 (163 mL) were added 1,4-benzoquinone (72 mg, 0.67 mmol) and Grubbs catalyst, second generation (138 mg, 0.160 mmol), and the mixture heated to reflux for 2 h. The mixture was cooled to room temperature and the solvent removed *in vacuo*. The crude material was purified by flash column chromatography (25-100% EtOAc in cyclohexane) to yield the title compound as a yellow solid with a *trans*/*cis* ratio of approximately 9:1 (1.22 g, 1.34 mmol, 83%).

4:1 *Trans*/*cis* mixture

m.p.: 129-133 °C; R_f = 0.2 (EtOAc); $[\alpha]_D^{24}$ = +8 (*c* 1, MeOH); IR (solid) 3321, 2927, 1732 (C=O), 1622, 1547, 1243, 1088, 733; LCMS (Fast4min) t_r = 3.35 min, *m/z* 910 $[M + H]^+$; purity (AUC) > 95%; HRMS (ESI) *m/z* calcd for $C_{48}H_{60}N_7O_9S$ $[M + H]^+$ 910.4168, found $[M + H]^+$ 910.4175.

***Trans* isomer**

m.p.: 140-145 °C; R_f = 0.2 (EtOAc); ^{1}H NMR (500 MHz, MeOD) δ 1.30-1.40 (2H, m, $C\underline{H}_2$-31), 1.44 (6H, s, $C\underline{H}_3$-25, $C\underline{H}_3$-26), 1.47-1.52 (2H, m, $C\underline{H}_2$-32a, $C\underline{H}_2$-36a), 1.59-1.71 (2H, m, $C\underline{H}_2$-32b, $C\underline{H}_2$-36b), 1.84-1.87 (2H, m, $C\underline{H}_2$-35), 2.07 (3H, s, $C\underline{H}_3$-27/$C\underline{H}_3$-28/$C\underline{H}_3$-29), 2.17-2.24 (1H, m, $C\underline{H}_2$-37a), 2.29 (1H, dd, *J* = 10, 14 Hz, $C\underline{H}_2$-38a), 2.51 (3H, s, $C\underline{H}_3$-27/$C\underline{H}_3$-28/$C\underline{H}_3$-29), 2.57 (3H, s, $C\underline{H}_3$-27/$C\underline{H}_3$-28/$C\underline{H}_3$-29), 2.98 (2H, s, $C\underline{H}_2$-18), 3.02-3.11 (4H, m, $C\underline{H}_2$-13a, $C\underline{H}_2$-30, $C\underline{H}_2$-38b), 3.22-3.27 (1H, m, $C\underline{H}_2$-13b), 3.35-3.41 (1H, m, $C\underline{H}_2$-37b), 3.57 (3H, s, $OC\underline{H}_3$), 4.20-4.26 (1H, m, H-39), 4.35 (1H, dd, *J* = 4, 12 Hz, H-14), 4.52 (1H, dd, *J* = 7, 7 Hz, H-33), 4.69-4.78 (2H, m, $C\underline{H}_2$-10), 5.07 (2H, s, $C\underline{H}_2$-15), 5.80 (1H, d, *J* = 16 Hz, H-11), 7.03 (1H, dd, *J* = 7, 7 Hz, H-5), 7.10-7.13 (2H, m, H-2, H-6), 7.23 (1H, d, *J* = 8 Hz, H-7), 7.25-7.28 (1H, m, Ar-C$\underline{H}$), 7.30-7.33 (4H, m, 4 × Ar-C$\underline{H}$), 7.62 (1H, d, *J* = 8 Hz, H-4); ^{13}C NMR (126 MHz, MeOD) δ 11.1 (C-27/C-28/C-29), 17.0 (C-27/C-28/C-29), 18.2 (C-27/C-28/C-29), 22.8 (C-32/C-36), 24.6 (C-31), 26.9 (C-13), 27.3 (C-25, C-26), 28.9 (C-32/C-36), 34.9 (C-35/C-38), 34.9 (C-38/C-35), 40.3 (C-30), 42.5 (C-18), 45.9 (C-10), 48.5 (C-37), 49.8 (C-33), 51.4 ($O\underline{C}H_3$), 56.6 (C-14), 66.2 (C-15), 68.1 (C-34), 86.2 (C-17), 108.7 (C-3), 109.5 (C-7), 117.0 (Pbf-Cq), 118.6 (C-5), 118.7 (C-4), 121.1 (C-6), 124.5 (C-39), 124.6 (Pbf-Cq), 127.3 (2 × Ar-$\underline{C}$H), 127.5 (C-2), 127.6 (Ar-$\underline{C}$H), 128.1 (2 × Ar-$\underline{C}$H), 128.2 (C-8), 130.1 (C-11), 132.1 (Pbf-Cq), 133.1 (Pbf-Cq), 135.8 (C-9), 136.8 (C-16), 138.0 (C-20), 156.5 ($N\underline{C}O_2$/$\underline{C}N_3$), 156.6 ($\underline{C}N_3$/$N\underline{C}O_2$), 158.4 (C-23), 169.0 (N$\underline{C}$OC), 172.3 (N$\underline{C}$OC), 173.9 ($\underline{C}O_2CH_3$); $[\alpha]_D^{26}$ = +15 (*c* 0.1, MeOH); IR (solid) 3343, 2923, 1732 (C=O), 1621, 1548, 1246, 1089, 739; LCMS (Fast4min) t_r = 3.36 min, *m/z* 910 $[M + H]^+$; purity (AUC) 93%; HRMS (ESI) *m/z* calcd for $C_{48}H_{60}N_7O_9S$ $[M + H]^+$ 910.4168, found $[M + H]^+$ 910.4162.

Methionine Coupling Product (13) and Oxidised Form (14)

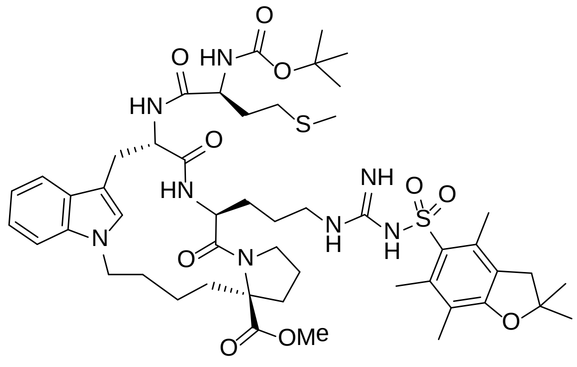

13

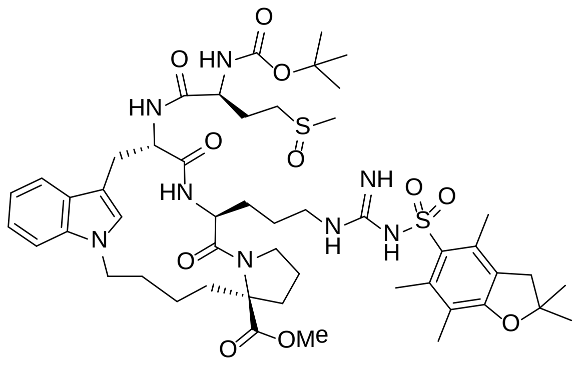

14

To a solution of *N*-α-*t*-Boc-L-methionine (13 mg, 0.05 mmol) in DMF (0.43 mL) were added DIPEA (0.20 mL, 1.10 mmol) and HBTU (18 mg, 0.05 mmol) and the mixture was stirred for 40 min at room temperature. Hydrogenation product **(4)** (10 mg, 0.013 mmol) was added and the mixture stirred for 105 min at room temperature. EtOAc (10 mL) and brine (10 mL) were added and the organic layer was separated, dried ($MgSO_4$) and concentrated *in vacuo* to yield crude **13**. The crude material was subjected to flash column chromatography (0-100% 2:3:8 water / propan-2-ol / EtOAc in EtOAc), which yielded trace amounts of **14**.

Methionine Coupling Product (13)

LCMS (Fast4min) t_r = 3.38 min, *m/z* 1009 $[M + H]^+$; purity (AUC) 71%; HRMS (ESI) *m/z* calcd for $C_{50}H_{73}N_8O_{10}S_2$ $[M + H]^+$ 1009.4886, found $[M + H]^+$ 1009.4841.

Oxidised Form (14)

LCMS (Fast4min) t_r = 3.25 min, *m/z* 1025 $[M + H]^+$; purity (AUC) 45%; HRMS (ESI) *m/z* calcd for $C_{50}H_{73}N_8O_{11}S_2$ $[M + H]^+$ 1025.4835, found $[M + H]^+$ 1025.4793.

Phenylalanine Coupling Product (15)

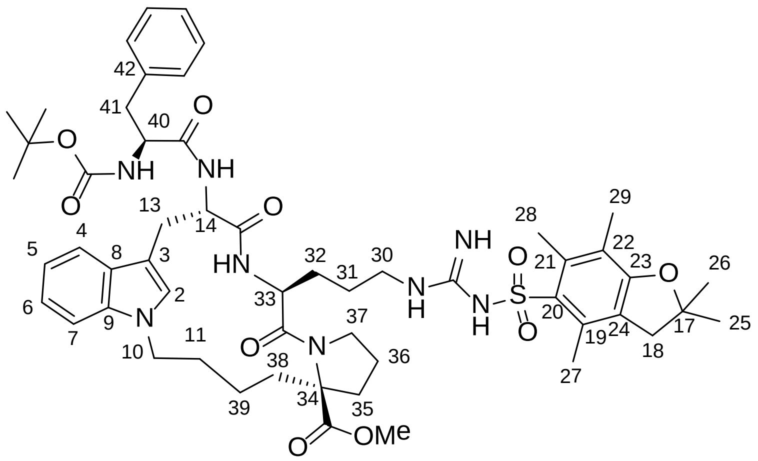

To a solution of *N*-α-*t*-Boc-L-phenylalanine (1.00 g, 3.77 mmol) in DMF (20 mL) were added DIPEA (1.85 mL, 10.6 mmol) and HBTU (1.30 g, 3.43 mmol) and the mixture was stirred at room temperature for 35 min. A solution of hydrogenation product **(4)** (741 mg, 0.95 mmol) in DMF (25 mL) was added and the mixture stirred for a further 90 min at room temperature. EtOAc (50 mL) and brine (50 mL) were added, and the organic layer was collected, dried ($MgSO_4$) and concentrated *in vacuo*. The crude material was purified by flash column chromatography (0-100% EtOAc in cyclohexane then 0-2% MeOH in EtOAc) to yield the title compound as a yellow solid (662 mg, 0.646 mmol, 68%).

m.p.: 116-120 °C; R_f = 0.7 (EtOAc); ^{1}H NMR (500 MHz, MeOD) δ 0.81-0.93 (1H, m, C$\underline{H}_2$-39a), 1.16-1.20 (1H, m, C$\underline{H}_2$-39b), 1.30-1.35 (9H, m, C(C$\underline{H}_3$)$_3$), 1.43 (6H, s, C$\underline{H}_3$-25, C$\underline{H}_3$-26), 1.56-1.94 (9H, m, C$\underline{H}_2$-11, C$\underline{H}_2$-31, C$\underline{H}_2$-32, C$\underline{H}_2$-36, C$\underline{H}_2$-38a), 2.04-2.07 (5H, m, C$\underline{H}_2$-27/C$\underline{H}_2$-28/C$\underline{H}_2$-29, C$\underline{H}_2$-35), 2.53 (3H, s, C$\underline{H}_2$-27/C$\underline{H}_2$-28/C$\underline{H}_2$-29), 2.60 (3H, s, C$\underline{H}_2$-27/C$\underline{H}_2$-28/C$\underline{H}_2$-29), 2.66 (1H, dd, *J* = 12, 12 Hz, C$\underline{H}_2$-38b), 2.81-2.97 (3H, m, C$\underline{H}_2$-18, C$\underline{H}_2$-41a), 3.12-3.17 (4H, m, C$\underline{H}_2$-41b, C$\underline{H}_2$-13a, C$\underline{H}_2$-30), 3.30-3.34 (1H, m, C$\underline{H}_2$-13b), 3.63 (3H, s, OC$\underline{H}_3$), 3.69-3.71 (2H, m, C$\underline{H}_2$-10), 4.34-4.42 (1H, m, H-40), 4.59-4.61 (1H, m, H-33), 4.74-4.75 (1H, m, H-14), 6.95 (1H, s, H-2), 7.03 (1H, dd, *J* = 7, 7 Hz, H-5), 7.12 (1H, dd, *J* = 7, 7 Hz, H-6), 7.16-7.20 (1H, m, Ar-C$\underline{H}$), 7.23-7.29 (5H, m, H-7, 4 × Ar-C$\underline{H}$), 7.52 (1H, d, *J* = 8 Hz, H-4); ^{13}C NMR (126 MHz, MeOD) δ 12.6 (C-27/C-28/C-29), 18.5 (C-27/C-28/C-29), 19.7 (C-27/C-28/C-29), 20.6 (C-39), 24.5 (C-36), 26.1 (C-31), 28.5 (1 × Boc-$\underline{C}$H$_3$), 28.7 (2 × Boc-$\underline{C}$H$_3$), 28.7 (C-13, C-25, C-26), 29.8 (C-32), 30.4 (C-11), 33.7 (C-38), 37.2 (C-35), 39.2 (C-41), 41.6 (C-30), 43.9 (C-18), 45.2 (C-10), 50.4 (C-37), 51.6 (C-33), 52.8 (O$\underline{C}$H$_3$), 55.5 (C-14), 57.3 (C-40), 70.2 (C-34), 80.6 ($\underline{C}$(CH$_3$)$_3$), 87.6 (C-17), 108.7 (C-3), 110.4 (C-7), 118.3 (Pbf-Cq), 119.9 (C-4), 120.0 (C-5), 122.3 (C-6) 125.9 (Pbf-Cq), 127.7 (Ar-$\underline{C}$H), 129.4 (2 × Ar-$\underline{C}$H), 129.8 (C-8), 129.9 (C-2), 130.4 (2 × Ar-CH), 133.4 (Pbf-Cq), 134.5 (Pbf-Cq), 137.0 (C-9), 138.5 (C-42), 139.5 (Pbf-Cq), 157.4 (N$\underline{C}$O$_2$), 157.9 ($\underline{C}$N$_3$), 159.7 (C-23), 171.9 (N$\underline{C}$OC), 172.9 (N$\underline{C}$OC), 173.6 (N$\underline{C}$OC), 175.7 ($\underline{C}$O$_2$CH$_3$); $[\alpha]_D^{23}$ = +2 (*c* 1, MeOH); IR (solid) 3321,

2933, 1735, 1642, 1518, 1436, 1242, 739, 659, 566; LCMS (Fast4min) t_r = 3.45 min, *m/z* 191 $[C_{13}H_{19}O]^+$ (pentamethyldihydrobenzofuran); purity (AUC) > 95%; HRMS (ESI) *m/z* calcd for $C_{54}H_{73}N_8O_{10}S$ $[M + H]^+$ 1025.5165, found $[M + H]^+$ 1025.5171.

Ester Hydrolysis Product (16)

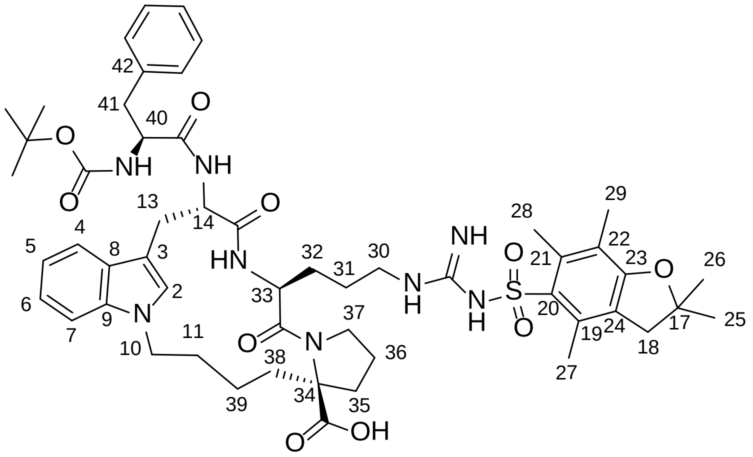

To a solution of phenylalanine coupling product **15** (531 mg, 0.518 mmol) in MeOH (60 mL) at 0 °C was added LiOH (1 M in H_2O, 20 mL, 20 mmol). The mixture was warmed to room temperature, THF (100 mL) was added and the mixture was stirred at 50 °C for 9 h. The mixture was cooled to room temperature and the solvents were removed *in vacuo*. Water (50 mL) and Et_2O (50 mL) were added and the layers separated. The aqueous layer was acidified to pH 1 with HCl (2 M in H_2O), and EtOAc (50 mL) was added. The layers were separated and the organic layer was dried ($MgSO_4$) and concentrated *in vacuo*. The crude material was purified by flash column chromatography (0-100% 2:3:8 water / propan-2-ol / EtOAc in EtOAc) to yield the title compound as an off-white solid (450 mg, 0.45 mmol, 86%).

m.p.: 163-166 °C; R_f = 0.2 (1:2:9 water / propan-2-ol / EtOAc); ^{1}H NMR (500 MHz, CD_3OD) δ 0.80-0.90 (1H, m, CH_2-39a), 1.20-1.27 (1H, m, CH_2-39b), 1.29-1.36 (9H, m, $C(CH_3)_3$), 1.44 (6H, s, CH_3-25, CH_3-26), 1.57-1.76 (6H, m, CH_2-11a, CH_2-31, CH_2-32, CH_2-38a), 1.83-1.99 (3H, m, CH_2-11b, CH_2-36), 2.07 (3H, s, CH_3-27/CH_3-28/CH_3-29), 2.08-2.14 (2H, m, CH_2-35), 2.52 (3H, s, CH_3-27/CH_3-28/CH_3-29), 2.59 (3H, s, CH_3-27/CH_3-28/CH_3-29), 2.65-2.72 (1H, m, CH_2-38b), 2.85-2.95 (1H, m, CH_2-41a), 2.97 (2H, s, CH_2-18), 3.12-3.20 (4H, m, CH_2-41b, CH_2-13a, CH_2-30), 3.30-3.32 (1H, m, CH_2-13b), 3.70-3.75 (2H, m, CH_2-37), 4.08-4.12 (1H, m, CH_2-10a), 4.20-4.26 (1H, m, CH_2-10b), 4.30-4.39 (1H, m, H-40), 4.60-4.64 (1H, m, H-33), 4.71 (1H, dd, J = 3, 8 Hz, H-14), 6.99 (1H, s, H-2), 7.04 (1H, dd, J = 7, 7 Hz, H-5), 7.13 (1H, dd, J = 8, 8 Hz, H-6), 7.17-7.32 (6H, m, H-7, 5 × Ar-CH), 7.53 (1H, d, J = 8 Hz, H-4); ^{13}C NMR (126 MHz, CD_3OD) δ 11.1 (C-27/C-28/C-29), 17.0 (C-27/C-28/C-29), 18.2 (C-27/C-

28/C-29), 19.6 (C-39), 23.1 (C-36), 24.6 (C-31), 27.1 (1 × Boc-$\underline{C}H_3$), 27.3 (2 × Boc-$\underline{C}H_3$), 27.3 (C-13, C-25, C-26), 28.3 (C-32), 29.2 (C-11), 32.5 (C-38), 36.1 (C-35), 37.8 (C-41), 40.2 (C-30), 42.6 (C-18), 44.0 (C-10), 49.1 (C-37), 50.3 (C-33), 54.2 (C-14), 56.0 (C-40), 69.2 (C-34), 79.3 ($\underline{C}(CH_3)_3$), 86.3 (C-17), 107.3 (C-3), 109.0 (C-7), 117.0 (Pbf-Cq), 118.6 (C-4/C-5), 118.6 (C-5/C-4), 120.9 (C-6), 124.6 (Pbf-Cq), 126.4 (Ar-$\underline{C}$H), 128.1 (2 × Ar-$\underline{C}$H), 128.5 (C-8), 128.7 (C-2), 129.0 (2 × Ar-$\underline{C}$H), 132.1 (Pbf-Cq), 133.0 (Pbf-Cq), 135.7 (C-9), 137.2 (C-42), 138.0 (Pbf-Cq), 156.1 ($N\underline{C}O_2/\underline{C}N_3$), 156.7 ($\underline{C}N_3/N\underline{C}O_2$), 158.4 (C-23), 170.4 (N$\underline{C}$OC), 170.6 (N$\underline{C}$OC), 172.3 (N$\underline{C}$OC), 176.5 ($\underline{C}O_2H$); $[\alpha]_D^{24}$ = -2 (*c* 1, MeOH); IR (solid) 3334, 2928, 1634, 1548, 1451, 1367, 1243, 1158, 1089, 737, 659, 566; LCMS (Fast4min) t_r = 3.41 min, *m/z* 191 $[C_{13}H_{19}O]^+$ (pentamethyldihydrobenzofuran); purity (AUC) > 95%; HRMS (ESI) *m/z* calcd for $C_{53}H_{71}N_8O_{10}S$ $[M + H]^+$ 1011.5008, found $[M + H]^+$ 1011.5011.

Final constraint peptide (18)

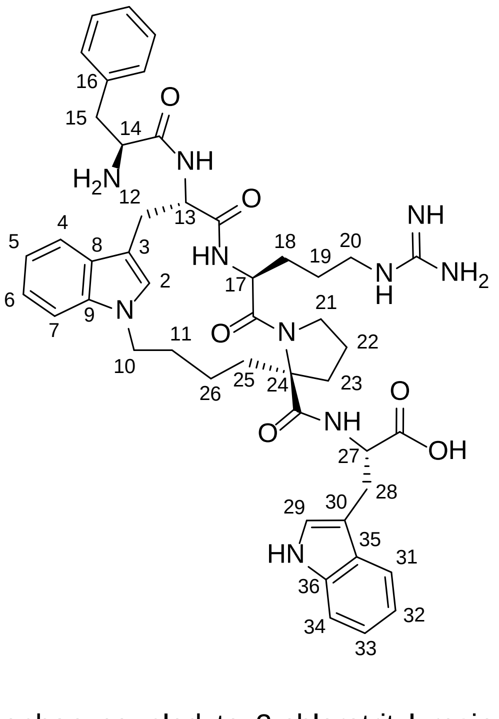

To 375 mg beads of tryptophan coupled to 2-chlorotrityl resin (0.2 mmol) was added a solution of product **16** (200 mg, 0.2 mmol), HATU (71 mg, 0.19 mmol) and DIPEA (0.74 mL, 4.2 mmol) in NMP (14 mL). The resulting suspension was shaken for 3 hours. The resin was collected and rinsed twice with DMF, dichloromethane and methanol. To cleave the final product from the resin, the beads were treated with a mixture of 94% TFA, 2.5% H_2O, 2.5% EDT and 1% TIS and shaken for 3 hours then the beads were drained and rinsed twice with more TFA. The TFA fractions were combined, concentrated in vacuum and the crude product was purified by RP-HPLC purification using 15 min gradient elution from 10:90 to

100:0 MeOH:water (both modified with 0.1% formic acid) at a flow rate of 20 mL/min to yield white solid (17 mg, 0.02 mmol, 10%); m.p.: 169-173 °C; ^{1}H NMR (500 MHz, MeOD) δ 0.40-0.51 (1H, m, CH_2-26a), 1.11-1.36 (5H, m, CH_2-26b, CH_2-18, CH_2-19), 1.45-1.52 (1H, m, CH_2-22a), 1.65-1.77 (5H, m, CH_2-22b, CH_2-11, CH_2-25), 1.90-1.97 (1H, m, CH_2-23a), 2.26-2.33 (1H, m, CH_2-23b), 2.63-2.73 (2H, m, CH_2-15a, CH_2-20a), 3.06-3.12 (1H, m, CH_2-20b), 3.15-3.19 (1H, m, CH_2-15b), 3.26-3.42 (6H, m, CH_2-12, H-14, CH_2-21a, CH_2-28), 3.50-3.55 (1H, m, CH_2-21b), 3.87-3.92 (1H, m, CH_2-10a), 4.37-4.41 (1H, m, CH_2-10b), 4.47-4.50 (1H, m, H-13/H-27), 4.63-4.67 (1H, m, H-17), 4.89-4.91 (1H, m, H-27/H-13), 6.97-7.00 (3H, m, 3 × Ar-CH), 7.08 (1H, dd, J = 7, 7 Hz, Ar-CH), 7.22-7.43 (9H, m, 9 × Ar-CH), 7.58 (1H, d, J = 8 Hz, Ar-CH), 7.69 (1H, d, J = 8 Hz, Ar-CH); ^{13}C NMR (126 MHz, MeOD) δ 21.3 (C-26), 23.3 (C-22), 24.8 (C-18/C-19), 27.6 (C-12/C-28), 28.7 (C-28/C-12), 29.7 (C-11/C-25), 30.1 (C-19/C-18), 35.1 (C-25/C-11), 36.0 (C-23), 38.7 (C-15), 42.0 (C-20), 47.2 (C-10), 49.9 (C-17), 50.9 (C-21), 56.2 (C-13/C-27), 56.5 (C-14), 56.9 (C-27/C-13), 73.7 (C-24), 108.4 (C-3/C-30), 111.7 (C-30/C-3), 112.0 (Ar-CH), 112.1 (Ar-CH), 119.6 (Ar-CH), 119.7 (Ar-CH), 119.7 (Ar-CH), 120.1 (Ar-CH), 122.0 (Ar-CH), 122.3 (Ar-CH), 124.4 (Ar-CH), 128.9 (Ar-CH), 129.5 (C-8/C-35), 129.8 (C-35/C-8), 130.4 (2 × Ar-CH), 130.5 (2 × Ar-CH), 130.7 (Ar-CH), 135.6 (C-16), 136.8 (C-9/C-36), 137.8 (C-36/C-9), 158.7 (CN_3), 170.0 (CNO), 172.3 (CNO), 172.7 (CNO), 174.4 (CNO), 178.8 (CO_2H); $[\alpha]_D^{22}$ = -80 (c 1, MeOH); IR (solid) 2927, 1630, 1435, 1200, 1174, 1127, 1014, 743, 719; LCMS (Fast4min) t_r = 2.27 min, m/z 845 $[M + H]^+$; purity (AUC) > 95%; HRMS (ESI) m/z calcd for $C_{46}H_{56}N_{10}O_6Na$ $[M + Na]^+$ 867.4277, found $[M + Na]^+$ 867.4284.

1*H*-Indole-3-carbaldehyde (19)

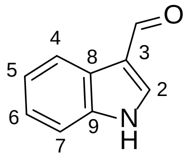

To a stirred flask of DMF (2.83 mL, 36.7 mmol) was added dropwise $POCl_3$ (0.87 mL, 9.3 mmol), keeping the temperature at 10-20 °C. A solution of indole (1.00 g, 8.54 mmol) in DMF (1.67 mL) was added slowly, keeping the temperature at 20-30 °C. The mixture was heated to 35 °C for 45 min. 3.5 g crushed ice was added, the mixture was stirred vigorously, and a further 3.5 g crushed ice was added. A solution of NaOH (3.77 g, 94.3 mmol) in water (10 mL) was added by dropping funnel, slowly at first then more rapidly. The solution was brought to the boil for 15 min, and the product collected by filtration and washed several

times with water. The title compound was isolated (1.12 g, 7.73 mmol, 91%) as a yellow solid which possessed spectroscopic data that were consistent with those in the literature.[14]

m.p: 191 °C, lit[15] 191-193 °C; R_f = 0.6 (9:1 CH_2Cl_2 / MeOH); ^{1}H NMR (500 MHz, $(CD_3)_2SO$) δ 7.21 (1H, ddd, J = 1, 8, 8 Hz, H-5) 7.26 (1H, ddd, J = 1, 8, 8 Hz, H-6), 7.51 (1H, d, J = 8 Hz, H-7), 8.09 (1H, d, J = 8 Hz, H-4), 8.27 (1H, s, H-2), 9.94 (1H, s, C<u>H</u>O), 12.09 (1H, br s, N<u>H</u>); ^{13}C NMR (126 MHz, $(CD_3)_2SO$) δ 112.4 (C-7), 118.2 (C-3), 120.8 (C-4), 122.1 (C-5), 123.4 (C-6), 124.1 (C-8), 137.0 (C-9), 138.3 (C-2), 184.9 (<u>C</u>HO); IR (solid) 3199-1721, 1627 (C=O), 1573, 1436, 1240, 755; LCMS (Fast4min) t_r = 2.03 min, m/z 146 $[M + H]^+$, purity (AUC) > 95%; HRMS (ESI) m/z calcd for C_9H_8NO $[M + H]^+$ 146.0600, found $[M + H]^+$ 146.0596; Anal. calcd for C_9H_7NO: C, 74.47; H, 4.86; N, 9.65. Found: C, 74.38; H, 4.81; N, 9.74.

***tert*-Butyl 3-formyl-1*H*-indole-1-carboxylate (20)**

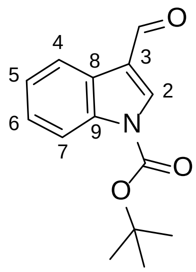

To a solution of $(Boc)_2O$ (2.48 g, 11.4 mmol) in MeCN (60 mL) was added 1*H*-indole-3-carbaldehyde **(19)** (1.10 g, 7.58 mmol) followed by DMAP (93 mg, 0.76 mmol). After stirring at room temperature for 15 min, imidazole (516 mg, 7.58 mmol) was added. After a further 5 min at room temperature, the reaction mixture was diluted with $CHCl_3$ (75 mL). The mixture was washed with HCl (0.5% in H_2O, 3 × 150 mL), and the organic layer was dried ($MgSO_4$) and concentrated *in vacuo* to yield the title compound (1.33 g, 5.42 mmol, 72%) as a pale yellow solid which possessed spectroscopic data that were consistent with those in the literature.[16]

m.p: 122 °C, lit[17] 121-123 °C; R_f = 0.8 (19:1 CH_2Cl_2 / MeOH); ^{1}H NMR (500 MHz, $CDCl_3$) δ 1.73 (9H, s, C(C<u>H</u>$_3$)$_3$), 7.39 (1H, ddd, J = 1, 8, 7 Hz, H-5), 7.43 (1H, ddd, J = 1, 8, 8 Hz, H-6), 8.17 (1H, d, J = 8 Hz, H-7), 8.25 (1H, s, H-2), 8.31 (1H, d, J = 7 Hz, H-4), 10.12 (1H, s, C<u>H</u>O); ^{13}C NMR (126 MHz, $CDCl_3$) δ 28.1 (C(<u>C</u>H$_3$)$_3$), 85.6 (<u>C</u>(CH$_3$)$_3$), 115.2 (C-7), 121.6 (C-3/C-8), 122.1 (C-4), 124.6 (C-5), 126.1 (C-6), 126.2 (C-8/C-3), 136.0 (C-9), 136.4 (C-2), 148.8 (N<u>C</u>O), 185.7 (<u>C</u>HO); LCMS (Fast4min) t_r = 3.16 min, m/z 146 $[M + H]^+$, purity (AUC) >

95%; HRMS (ESI) *m/z* calcd for C_9H_8NO [M – Boc + H]$^+$ 146.0600, found [M – Boc + H]$^+$ 146.0599; Anal. calcd for $C_{14}H_{15}NO_3$: C, 68.56; H, 6.16; N, 5.71. Found: C, 68.69; H, 6.08; N, 5.62.

***tert*-Butyl 3-(hydroxymethyl)-1*H*-indole-1-carboxylate (21)**

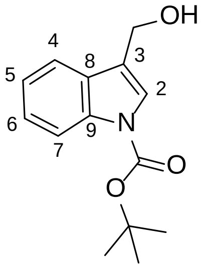

To a suspension of *tert*-butyl 3-formyl-1*H*-indole-1-carboxylate **(20)** (1.30 g, 5.30 mmol) in ethanol (3.5 mL) was added $NaBH_4$ (413 mg, 10.9 mmol) slowly to keep the temperature below 20 ^oC. The mixture was allowed to warm to room temperature and stirred for 22 h. The solvent was removed *in vacuo*, the residual oil shaken with NaOH (1 M in H_2O, 13.7 mL) and the alkaline solution extracted with Et_2O (3 × 20 mL). The organic layer was dried ($MgSO_4$) and concentrated *in vacuo* to yield the title compound (1.12 g, 4.53 mmol, 85%) as a colourless oil slowly crystallising to a yellow solid which possessed spectroscopic data that were consistent with those in the literature.[18]

m.p: 59-63 °C; R_f = 0.2 (4:1 cyclohexane / EtOAc); ^{1}H NMR (500 MHz, $CDCl_3$) δ 1.68 (9H, s, C(C$\underline{H}_3$)$_3$), 4.86 (2H, d, J = 1 Hz, C$\underline{H}_2$OH), 7.28 (1H, ddd, J = 1, 8, 8 Hz, H-5), 7.36 (1H, ddd, J = 1, 6, 8 Hz, H-6), 7.60 (1H, s, H-2), 7.67 (1H, d, J = 8 Hz, H-4), 8.16 (1H, br d, J = 6 Hz, H-7); ^{13}C NMR (126 MHz, $CDCl_3$) δ 28.2 (C($\underline{C}H_3$)$_3$), 57.2 ($\underline{C}H_2$OH), 83.8 ($\underline{C}$(CH_3)$_3$), 115.4 (C-7), 119.3 (C-5), 120.4 (C-3), 122.7 (C-6), 123.8 (C-2), 124.7 (C-4), 129.2 (C-8), 135.8 (C-9), 149.7 (N$\underline{C}$O); IR (solid) 3353 (O-H), 1725 (C=O), 1452, 1360, 1150, 1082, 745; LCMS (Fast4min) t_r = 2.97 min, *m/z* 130 [M – OH – Boc + H]$^+$, purity (AUC) > 95%; Anal. calcd for $C_{14}H_{17}NO_3$: C, 68.00; H, 6.93; N, 5.66. Found: C, 68.08; H, 7.00; N, 5.71.

tert-Butyl 3-(bromomethyl)-1H-indole-1-carboxylate (22)

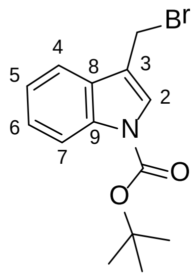

A solution of *tert*-butyl 3-(hydroxymethyl)-1*H*-indole-1-carboxylate **(21)** (100 mg, 0.405 mmol) and CBr_4 (159 mg, 0.486 mmol) in CH_2Cl_2 (1 mL) was cooled to 0 ^{o}C and triphenylphosphine (133 mg, 0.506 mmol) was added portionwise. The ice bath was removed and the reaction stirred for 1 h. A 4:1 mixture of cyclohexane and EtOAc (6 mL) was added and the resulting precipitate filtered over a small pad of silica gel. The pad was washed with the same solvent mixture (5 × 1.5 mL) and the filtrate concentrated *in vacuo* to yield the title compound (109 mg, 0.350 mmol, 87%) as a yellow oil slowly crystallising to a brown solid which possessed spectroscopic data that were consistent with those in the literature.[19]

m.p: 118-119 °C, lit[20] 106-107 °C; R_f = 0.3 (9:1 cyclohexane / EtOAc); ^{1}H NMR (500 MHz, $CDCl_3$) δ 1.68 (9H, s, C(C<u>H</u>$_3$)$_3$), 4.71 (2H, s, C<u>H</u>$_2$Br), 7.33 (1H, ddd, *J* = 1, 7, 8 Hz, H-5), 7.38 (1H, ddd, *J* =1, 7, 8 Hz, H-6), 7.69-7.71 (2H, m, H-2, H-4), 8.16 (1H, br d, *J* = 7 Hz, H-7); ^{13}C NMR (126 MHz, $CDCl_3$) δ 24.6 (<u>C</u>H$_2$Br), 28.2 (C(<u>C</u>H$_3$)$_3$), 84.2 (<u>C</u>(CH$_3$)$_3$), 115.5 (C-7), 117.2 (C-3), 119.3 (C-4), 122.9 (C-5), 125.0 (C-6), 125.1 (C-2), 128.7 (C-8) C-9 not observed; LCMS (Fast4min) t_r = 3.25 min, LCMS (Fast4min) *tr* = 3.25 min, purity (AUC) = 92%; compound do not ionise in MS.

(*R*)-3,6-Diethoxy-2-isopropyl-2,5-dihydropyrazine (23)

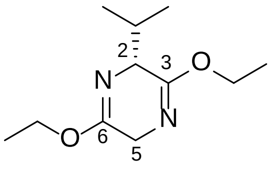

(*R*)-3-Isopropylpiperazine-2,5-dione (278 mg, 1.78 mmol) and $Et_3O\cdot BF_4$ (1.21 g, 6.37 mmol) were dissolved in CH_2Cl_2 (9 mL) and stirred at room temperature for 4 days. The solution was added portionwise to a vigorously stirred mixture of saturated aqueous $NaHCO_3$ (10 mL) and CH_2Cl_2 (10 mL) at 0 °C, while the pH was adjusted to 8-9 by the addition of NaOH (3 M in H_2O, 3.7 mL). The phases were separated and the aqueous phase extracted with

CH_2Cl_2 (2 × 10 mL). The combined organic layers were washed with brine (20 mL), dried ($MgSO_4$) and concentrated *in vacuo* to yield the title compound (343 mg, 1.62 mmol, 90%) as a colourless oil which possessed spectroscopic data that were consistent with those in the literature.[21]

R_f = 0.3 (9:1 cyclohexane / EtOAc); ^{1}H NMR (500 MHz, $CDCl_3$) δ 0.79 (3H, d, *J* = 7 Hz, $CH(C\underline{H}_3)_2$), 1.04 (3H, d, *J* = 7 Hz, $CH(C\underline{H}_3)_2$), 1.29 (3H, t, *J* = 7 Hz, $OCH_2C\underline{H}_3$), 1.30 (3H, t, *J* = 7 Hz, $OCH_2C\underline{H}_3$), 2.25 (1H, dqq, *J* = 3, 7, 7 Hz, $C\underline{H}(CH_3)_2$), 3.93-4.25 (7H, m, 2 × $OC\underline{H}_2CH_3$, H-2, $C\underline{H}_2$-5); ^{13}C NMR (126 MHz, $CDCl_3$) δ 14.3 (2 × $OCH_2\underline{C}H_3$), 17.1 (1 × $CH(\underline{C}H_3)_2$), 19.0 (1 × $CH(\underline{C}H_3)_2$), 32.6 ($\underline{C}H(CH_3)_2$), 46.7 (C-5), 60.8 ($O\underline{C}H_2CH_3$), 60.9 ($O\underline{C}H_2CH_3$), 61.1 (C-2), 161.8 (C-6), 164.4 (C-3); $[\alpha]_D^{22}$ = -57 (*c* 0.2, CH_2Cl_2), lit[231] $[\alpha]_D^{22}$ = -79 (no *c* or solvent specified); IR (oil) 2964, 1676 (C=N), 1224 (=C-O-C), 1031 (=C-O-C), 756; LCMS (Fast4min) t_r = 2.96 min, *m/z* 213 $[M + H]^+$, purity (AUC) > 95%; HRMS (ESI) *m/z* calcd for $C_{11}H_{21}N_2O_2$ $[M + H]^+$ 213.1598, found $[M + H]^+$ 213.1597.

tert-Butyl 3-(((2_S_,5_R_)-3,6,diethoxy-5-isopropyl-2,5-dihydropyrazin-2-yl)methyl)-1_H_-indole-1-carboxylate (24)

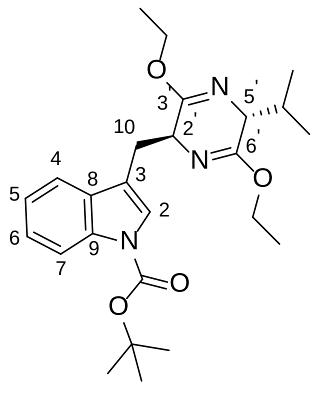

A solution of (*R*)-3,6-diethoxy-2-isopropyl-2,5-dihydropyrazine **(23)** (116 mg, 0.546 mmol) in THF (2 mL) was cooled to -78 °C and *n*-BuLi (2.5 M in hexanes, 0.24 mL, 0.60 mmol) was added dropwise. After stirring at -78 °C for 30 min, a solution of *tert*-butyl 3-(bromomethyl)-1*H*-indole-1-carboxylate **(22)** (149 mg, 0.479 mmol) in THF (1 mL) was added. The mixture was stirred at -78 °C for 12 h, and then allowed to slowly warm to room temperature. The solution was concentrated *in vacuo* and the residue taken up in a saturated aqueous solution of $NaHCO_3$ (20 mL). The aqueous layer was extracted with CH_2Cl_2 (2 × 20 mL), and the combined organic layers were washed with brine, dried ($MgSO_4$) and concentrated *in vacuo*. The crude material was purified by flash column chromatography (17:3

cyclohexane / Et_2O) to yield the title compound (91 mg, 0.21 mmol, 40%) as a white solid which possessed spectroscopic data that were consistent with those in the literature.[22] The undesired *2R*,5*R* diastereomer was not isolated but analysis of the crude ^{1}H NMR revealed a diastereomeric ratio of 3:1 in favour of the desired compound.

m.p: 48-52 °C; R_f = 0.3 (9:1 cyclohexane / EtOAc); ^{1}H NMR (500 MHz, $CDCl_3$) δ 0.68 (3H, d, J = 7 Hz, CH(C<u>H</u>$_3$)$_2$), 0.97 (3H, d, J = 7 Hz, CH(C<u>H</u>$_3$)$_2$), 1.24 (3H, t, J = 7 Hz, OCH$_2$C<u>H</u>$_3$), 1.33 (3H, t, J = 7 Hz, OCH$_2$C<u>H</u>$_3$), 1.66 (9H, s, C(C<u>H</u>$_3$)$_3$), 2.19 (1H, dqq, J = 3, 7, 7 Hz, C<u>H</u>(CH$_3$)$_2$), 3.18 (1H, dd, J = 5, 14 Hz, H-10a), 3.23 (1H, dd, J = 4, 14 Hz, H-10b), 3.58 (1H, dd, J = 4, 5 Hz, H-2'), 4.01-4.20 (4H, m, 2 × OC<u>H</u>$_2$CH$_3$), 4.29-4.32 (1H, m, H-5'), 7.20 (1H, ddd, J = 1, 8, 8 Hz, H-5), 7.27 (1H, ddd, J = 1, 7, 8 Hz, H-6), 7.38 (1H, s, H-2), 7.60 (1H, d, J = 8 Hz, H-4), 8.10 (1H, br d, J = 7 Hz, H-7); ^{13}C NMR (126 MHz, $CDCl_3$) δ 13.4 (2 × OCH$_2$<u>C</u>H$_3$), 15.6 (1 × CH(<u>C</u>H$_3$)$_2$), 18.0 (1 × CH(<u>C</u>H$_3$)$_2$), 27.2 (C(<u>C</u>H$_3$)$_3$), 28.3 (C-10), 30.7 (<u>C</u>H(CH$_3$)$_2$), 55.1 (C-2'), 59.4 (2 × O<u>C</u>H$_2$CH$_3$), 59.5 (C-5'), 82.0 (<u>C</u>(CH$_3$)$_3$), 113.9 (C-7), 115.8 (C-3), 118.6 (C-4), 120.9 (C-5), 122.9 (C-6), 123.2 (C-2), 130.4 (C-8), 134.2 (C-9), 148.7 (N<u>C</u>O), 161.3 (C-3'/6'), 162.5 (C-6'/3'); $[\alpha]_D^{22}$ = +27 (*c* 1, MeOH), lit[23] $[\alpha]_D^{27}$ = +52, (*c* 1, MeOH); IR (solid) 1732 (C=O), 1691 (C=N), 1453, 1229 (=C-O-C), 1035 (=C-O-C), 745; LCMS (Fast4minLipophilic) t_r = 2.12 min, *m/z* 442 $[M + H]^+$ purity (AUC) = 89%; HRMS (ESI) *m/z* calcd for $C_{25}H_{35}N_3NaO_4$ $[M + Na]^+$ 464.2529, found $[M + Na]^+$ 464.2520.

***tert*-Butyl 2-bromo-3-methyl-1*H*-indole-1-carboxylate (25)**

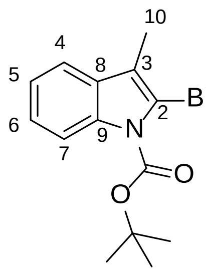

Method A (CCl_4)

To a solution of 3-methylindole (333 mg, 2.50 mmol) in CCl_4 (5 mL) was added NBS (451 mg, 2.50 mmol), and the mixture was stirred at room temperature for 20 min. The succinimide by-product was filtered off and washed with CCl_4 (2 × 0.5 mL). The combined filtrates were concentrated *in vacuo* and taken up in MeCN (7.5 mL). To the MeCN solution were added $(Boc)_2O$ (546 mg, 2.50 mmol) and DMAP (6 mg, 0.02 mmol). After stirring at room temperature for 55 min, EtOAc (10 mL) and HCl (2 M in H_2O, 2.5 mL) were added. The organic layer was separated, washed with brine (10 mL), dried ($MgSO_4$) and

concentrated *in vacuo*. The crude material was purified by flash column chromatography (9:1 cyclohexane / EtOAc) to yield the title compound (331 mg, 1.07 mmol, 43%) as a dark purple liquid which possessed spectroscopic data that were consistent with those in the literature.[24]

Method B (CH_2Cl_2)

To a solution of 3-methylindole (333 mg, 2.50 mmol) in CH_2Cl_2 (5 mL) was added NBS (451 mg, 2.50 mmol). After 10 min at room temperature the solvent was removed *in vacuo*. MeCN (7.5 mL) was added and the succinimide by-product filtered off. To the MeCN solution were added $(Boc)_2O$ (546 mg, 2.50 mmol) and DMAP (6 mg, 0.02 mmol). After stirring at room temperature for 10 min, EtOAc (10 mL) and HCl (2 M in H_2O, 2.5 mL) were added. The organic layer was separated, washed with brine (10 mL), dried ($MgSO_4$) and concentrated *in vacuo*. The crude material was purified by flash column chromatography (9:1 cyclohexane / EtOAc) to yield the title compound (343 mg, 1.11 mmol, 44%) as a dark purple oil which possessed spectroscopic data that were consistent with those in the literature.[24]

R_f = 0.5 (9:1 cyclohexane / EtOAc); ^{1}H NMR (500 MHz, $CDCl_3$) δ 1.71 (9H, s, C(C$\underline{H}_3$)$_3$), 2.28 (3H, s, C$\underline{H}_3$-10), 7.24 (1H, ddd, *J* = 1, 8, 8 Hz, H-5), 7.29 (1H, ddd, *J* = 2, 8, 9 Hz, H-6), 7.46 (1H, d, *J* = 8 Hz, H-4), 8.08 (1H, d, *J* = 9 Hz, H-7); ^{13}C NMR (126 MHz, $CDCl_3$) δ 10.2 ($\underline{C}H_3$-10), 28.2 (C($\underline{C}H_3$)$_3$), 84.6 ($\underline{C}$(CH$_3$)$_3$), 108.8 (C-3), 115.2 (C-7), 118.1 (C-4), 119.2 (C-2), 122.7 (C-5), 124.3 (C-6), 129.6 (C-8), 136.5 (C-9), 149.3 (N$\underline{C}$O); IR (oil) 2931, 1701 (C=O), 1620 (C=C aryl), 1470, 1447, 739; LCMS (Fast4min) t_r = 3.35 min, purity (AUC) > 95%.

***tert*-Butyl 2-bromo-3-(bromomethyl)-1*H*-indole-1-carboxylate (26)**

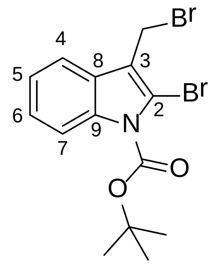

Method A (CCl_4)

A solution of *tert*-butyl 2-bromo-3-methyl-1*H*-indole-1-carboxylate **(25)** (149 mg, 0.480 mmol) in CCl_4 (3 mL) was heated to reflux, then NBS (92 mg, 0.52 mmol) and AIBN (3 mg, 0.02 mmol) were added. After 40 min at reflux, the mixture was cooled to room temperature and the solvent removed *in vacuo*. The succinimide by-product was filtered off and washed with CCl_4 (2 × 0.5 mL), and the combined filtrates were concentrated *in vacuo* to yield the title compound (107 mg, 0.275 mmol, 57%) as a yellow solid which possessed spectroscopic data that were consistent with those in the literature.[24]

Method B ($CHCl_3$)

A solution of *tert*-butyl 2-bromo-3-methyl-1*H*-indole-1-carboxylate **(25)** (166 mg, 0.535 mmol) in $CHCl_3$ (2.94 mL) was heated to reflux, then NBS (104 mg, 0.584 mmol) and AIBN (3 mg, 0.02 mmol) were added. After 40 min at reflux the mixture was cooled to room temperature and the solvent removed *in vacuo*. The residue was taken up in cyclohexane, the succinimide by-product filtered off and the cyclohexane solution concentrated *in vacuo* to yield the title compound (126 mg, 0.323 mmol, 60%) as a yellow solid which possessed spectroscopic data that were consistent with those in the literature.[24]

m.p: 96-102 °C; R_f = 0.3 (19:1 cyclohexane / EtOAc); ^{1}H NMR (500 MHz, $CDCl_3$) δ 1.72 (9H, s, C(C<u>H</u>$_3$)$_3$), 4.71 (2H, s, C<u>H</u>$_2$Br), 7.31 (1H, ddd, *J* = 1, 7, 8 Hz, H-5), 7.35 (1H, ddd, *J* = 2, 7, 8 Hz, H-6), 7.63 (1H, dd, *J* = 2, 7 Hz, H-4), 8.11 (1H, dd, *J* = 1, 7 Hz, H-7); ^{13}C NMR (126 MHz, $CDCl_3$) δ 24.0 (<u>C</u>H$_2$Br), 28.2 (C(<u>C</u>H$_3$)$_3$), 85.6 (<u>C</u>(CH$_3$)$_3$), 111.6 (C-2/C-3), 115.5 (C-7), 118.3 (C-4), 119.3 (C-3/C-2), 123.3 (C-5), 125.1 (C-6), 127.2 (C-8), 136.7 (C-9), 148.8 (N<u>C</u>O); LCMS (Fast4min) t_r = 3.36 min, *m/z* 209 [M – Br – Boc + H]$^+$, purity (AUC) = 88%; HRMS (ESI) *m/z* calcd for $C_9H_7{}^{81}BrN$ [M – Br – Boc + H]$^+$ 209.9736, found [M + H]$^+$ 209.9736.

(5*R*)-2-Benzyl-3,6-diethoxy-5-isopropyl-2,5-dihydropyrazine (27)

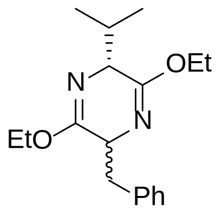

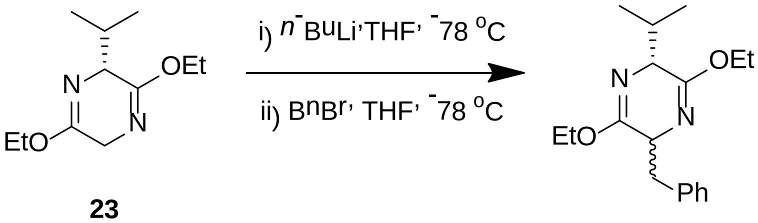

Scheme S1: Test reactions using benzyl bromide as a model electrophile.

Table S2: Conditions for benzyl bromide test reactions.

Entry	Eq of *n*-BuLi	Additive	LCMS Yield of 27[a]
**1**	1	-	50%
**2**	1.5	-	100%
**3**	1	DMPU	100%
**4**	1	CuI	100%
**5**	1	NaI	59%

[a]The diastereomeric purity of **27** was not determined

Entry 1

A solution of (*R*)-3,6-diethoxy-2-isopropyl-2,5-dihydropyrazine **(23)** (50 mg, 0.24 mmol) in THF (1.5 mL) was cooled to -78 °C and *n*-BuLi (2.5 M in hexanes, 90 µL, 0.24 mmol) was added dropwise. After stirring at -78 °C for 30 min, benzyl bromide (28 µL, 0.24 mmol) was added and the reaction monitored by LCMS. The final conversion to the title compound was approximately 50% by UV integration.

Entry 2

A solution of (*R*)-3,6-diethoxy-2-isopropyl-2,5-dihydropyrazine **(23)** (30 mg, 0.14 mmol) in THF (1 mL) was cooled to -78 °C and *n*-BuLi (2.5 M in hexanes, 85 µL, 0.21 mmol) was

added dropwise. After stirring at -78 °C for 30 min, benzyl bromide (17 µL, 0.14 mmol) was added and the reaction monitored by LCMS. The final conversion to the title compound was approximately 100% by UV integration.

Entry 3

A solution of (*R*)-3,6-diethoxy-2-isopropyl-2,5-dihydropyrazine **(23)** (30 mg, 0.14 mmol) in THF (1 mL) was cooled to -78 °C and *n*-BuLi (2.5 M in hexanes, 57 µL, 0.14 mmol) was added dropwise. After stirring at -78 °C for 30 min, DMPU (17 µL, 0.14 mmol) was added. After a further 30 min at -78 °C, benzyl bromide (17 µL, 0.14 mmol) was added and the reaction monitored by LCMS. The final conversion to the title compound was approximately 100% by UV integration.

Entry 4

A solution of (*R*)-3,6-diethoxy-2-isopropyl-2,5-dihydropyrazine **(23)** (20 mg, 0.094 mmol) in THF (1 mL) was cooled to -78 °C and *n*-BuLi (2.5 M in hexanes, 38 µL, 0.094 mmol) was added dropwise. After stirring at -78 °C for 30 min, CuI (18 mg, 0.094 mmol) was added. After a further 30 min at -78 °C, benzyl bromide (11 µL, 0.094 mmol) was added and the reaction monitored by LCMS. The final conversion to the title compound was approximately 100% by UV integration.

Entry 5

A solution of (*R*)-3,6-diethoxy-2-isopropyl-2,5-dihydropyrazine **(23)** (30 mg, 0.14 mmol) in THF (0.5 mL) was cooled to -78 °C, *n*-BuLi (2.5 M in hexanes, 57 µL, 0.14 mmol) was added dropwise, and the mixture stirred for 30 min. To a separate solution of benzyl bromide (17 µL, 0.14 mmol) in acetone (0.5 mL) was added NaI (25 mg, 0.14 mmol).[233] After 20 min this mixture was filtered, concentrated *in vacuo*, and the residue taken up in THF (0.5 mL). This benzyl iodide solution was transferred to the first solution at -78 °C and the reaction monitored by LCMS. The final conversion to the title compound was approximately 59% by UV integration.

LCMS (Fast4min) t_r = 2.79 min, *m/z* 303 $[M + H]^+$; HRMS (ESI) *m/z* calcd for $C_{18}H_{27}N_2O_2$ $[M + H]^+$ 303.2067, found $[M + H]^+$ 303.2036.

tert-Butyl 2-bromo-3-(((2_S_,5_R_)-3,6-diethoxy-5-isopropyl-2,5-dihydropyrazin-2-yl)methyl)-1_H_-indole-1-carboxylate (28) and _tert_-Butyl 2-bromo-3-(((2_R_,5_R_)-3,6-diethoxy-5-isopropyl-2,5-dihydropyrazin-2-yl)methyl)-1_H_-indole-1-carboxylate (29)

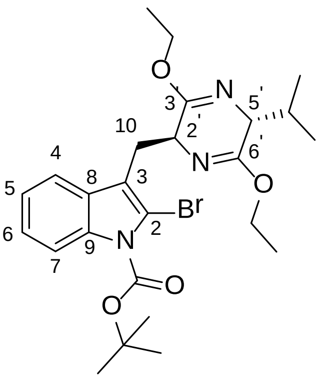

28

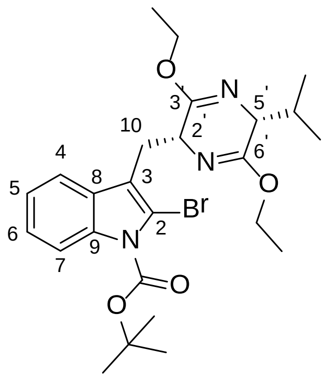

29

A solution of (*R*)-3,6-diethoxy-2-isopropyl-2,5-dihydropyrazine **(23)** (212 mg, 1.00 mmol) in THF (5 mL) was cooled to -78 °C and *n*-BuLi (2.5 M in hexanes, 0.40 mL, 1.0 mmol) was added dropwise. After stirring at -78 °C for 30 min, a solution of *tert*-butyl 2-bromo-3-(bromomethyl)-1*H*-indole-1-carboxylate **(26)** (260 mg, 0.670 mmol) in THF (10 mL) was added. The mixture was stirred at -78 °C for 10 h, and then allowed to slowly warm to room temperature. The reaction was quenched by the addition of a saturated aqueous solution of $NaHCO_3$ (25 mL), most of the THF was removed *in vacuo* and Et_2O (25 mL) was added. The aqueous layer was extracted with Et_2O (3 × 25 mL), and the combined organic layers were washed with brine (50 mL), dried ($MgSO_4$) and concentrated *in vacuo*. The crude material was purified by flash column chromatography (19:1 cyclohexane / EtOAc) to yield the desired 2*S*,5*R* diastereomer **28** (72 mg, 0.14 mmol, 20%) and the undesired 2*R*,5*R* diastereomer **29** (11 mg, 0.021 mmol, 3%) each as a colourless oil which possessed spectroscopic data that were consistent with those in the literature.[24]

tert-Butyl 2-bromo-3-(((2_S_,5_R_)-3,6-diethoxy-5-isopropyl-2,5-dihydropyrazin-2-yl)methyl)-1_H_-indole-1-carboxylate (28)

R_f = 0.3 (9:1 cyclohexane / EtOAc); ^{1}H NMR (500 MHz, $CDCl_3$) δ 0.67 (3H, d, J = 7 Hz, $CH(C\underline{H}_3)_2$), 1.00 (3H, d, J = 7 Hz, $CH(C\underline{H}_3)_2$), 1.20 (3H, t, J = 7 Hz, $OCH_2C\underline{H}_3$), 1.31 (3H, t, J = 7 Hz, $OCH_2C\underline{H}_3$), 1.71 (9H, s, $C(C\underline{H}_3)_3$), 2.23 (1H, dqq, J = 3, 7, 7 Hz, $C\underline{H}(CH_3)_2$), 2.99 (1H,

dd, *J* = 8, 14, H-10a), 3.36 (1H, dd, *J* = 4, 14, H-10b), 3.73 (1H, dd, *J* = 3, 3 Hz, H-5'), 3.95-4.30 (5H, m, 2 × $OC\underline{H}_2CH_3$, H-2'), 7.20 (1H, ddd, *J* = 1, 8, 8 Hz, H-5), 7.26 (1H, ddd, *J* = 1, 8, 8 Hz, H-6), 7.56 (1H, d, 8 Hz, H-4), 8.05 (1H, d, 8 Hz, H-7); ^{13}C NMR (126 MHz, $CDCl_3$) δ 14.3 ($OCH_2\underline{C}H_3$), 14.4 ($OCH_2\underline{C}H_3$), 16.6 (1 × $CH(\underline{C}H_3)_2$), 19.1 (1 × $CH(\underline{C}H_3)_2$), 28.2 ($C(\underline{C}H_3)_3$), 31.0 (C-10), 31.5 ($\underline{C}H(CH_3)_2$), 55.7 (C-2'), 60.5 (C-5'), 60.8 (2 × $O\underline{C}H_2CH_3$), 84.7 ($\underline{C}(CH_3)_3$), 110.3 (C-2/C-3), 115.0 (C-7), 119.1 (C-4), 120.6 (C-3/C-2), 122.4 (C-5), 124.1 (C-6), 129.6 (C-8), 136.5 (C-9), 149.2 (N$\underline{C}$O), 162.8 (C-3'), 163.4 (C-6'); material degraded before $[\alpha]_D$ could be obtained; LCMS (Fast4minLipophilic) t_r = 2.09 min, *m/z* 522 $[M + H]^+$, purity (AUC) = 66%; HRMS (ESI) *m/z* calcd for $C_{25}H_{34}{}^{81}BrN_3NaO_4$ $[M + Na]^+$ 544.1608, found $[M + Na]^+$ 544.1612.

tert-Butyl 2-bromo-3-(((2*R*,5*R*)-3,6-diethoxy-5-isopropyl-2,5-dihydropyrazin-2-yl)methyl)-1*H*-indole-1-carboxylate (29)

^{1}H NMR (500 MHz, $CDCl_3$) δ 0.80 (3H, d, *J* = 7 Hz, $CH(C\underline{H}_3)_2$), 1.05 (3H, d, *J* = 7 Hz, $CH(C\underline{H}_3)_2$), 1.21 (3H, t, *J* = 7 Hz, $OCH_2C\underline{H}_3$), 1.27 (3H, t, *J* = 7 Hz, $OCH_2C\underline{H}_3$), 1.71 (9H, s, $C(C\underline{H}_3)_3$), 2.06-2.14 (1H, br m, $C\underline{H}(CH_3)_2$), 2.97 (1H, dd, *J* = 9, 13 Hz, H-10a), 3.35 (1H, dd, *J* = 5, 13 Hz, H-10b), 3.88 (1H, dd, *J* = 4, 4 Hz, H-5'), 3.95-4.21 (4H, m, 2 × $OC\underline{H}_2CH_3$, 4.28-4.35 (1H, m, H-2'), 7.22 (1H, ddd, *J* = 1, 8, 8 Hz, H-5), 7.27 (1H, m, H-6), 7.56 (1H, br d, 8 Hz, H-4), 8.07 (1H, d, 8 Hz, H-7); ^{13}C NMR (126 MHz, $CDCl_3$) δ 14.4 ($OCH_2\underline{C}H_3$), 14.5 ($OCH_2\underline{C}H_3$), 17.9 (1 × $CH(\underline{C}H_3)_2$), 19.8 (1 × $CH(\underline{C}H_3)_2$), 28.4 ($C(\underline{C}H_3)_3$), 32.0 (C-10), 32.2 ($\underline{C}H(CH_3)_2$), 56.0 (C-2'), 60.7 (2 × $O\underline{C}H_2CH_3$), 61.3 (C-5'), 84.8 ($\underline{C}(CH_3)_3$), 110.4 (C-2/C-3), 115.3 (C-7), 119.1 (C-4), 119.3 (C-3/C-2), 122.7 (C-5), 124.4 (C-6), 129.7 (C-8), 136.8 (C-9), 149.4 (N$\underline{C}$O), 162.9 (C-3'/C-6'), 163.6 (C-6'/C-3'); material degraded before $[\alpha]_D$ could be obtained; LCMS (Fast4minLipophilic) t_r = 2.10 min, *m/z* 522 $[M + H]^+$, purity (AUC) = 60%; HRMS (ESI) *m/z* calcd for $C_{25}H_{35}{}^{81}BrN_3O_4$ $[M + H]^+$ 522.1789, found $[M + H]^+$ 522.1775.

tert-Butyl 3-((*tert*-butoxycarbonyloxy)methyl)-1*H*-indole-1-carboxylate (30) and 1-Allyl-1*H*-indole-3-carbaldehyde (31)

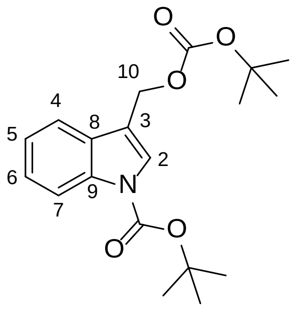

30

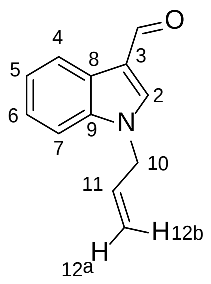

31

A solution of diisopropylamine (86 µL, 0.61 mmol) in THF (1.2 mL) was cooled to -78 °C and *n*-BuLi (2.5 M in hexanes, 0.25 mL, 0.63 mmol) added dropwise. The solution was stirred for 5 min, warmed to 0 °C and stirred for 15 min. A solution of *tert*-butyl 3-formyl-1*H*-indole-1-carboxylate **(20)** (100 mg, 0.408 mmol) in THF (1.6 mL) was cooled to -78 °C and the freshly-prepared LDA solution was added dropwise. After stirring at -78 °C for 60 min, allyl bromide (0.11 mL, 1.3 mmol) was added dropwise. The mixture was stirred at -78 °C for 60 min, warmed to room temperature and stirred for a further 16 h. The solvent was removed *in vacuo* and the residue was taken up in CH_2Cl_2 (25 mL) and washed with $NaHCO_3$ (5% in H_2O, 25 mL). The aqueous layer was extracted with CH_2Cl_2 (25 mL) and the combined organic layers dried ($MgSO_4$) and concentrated *in vacuo*. The crude material was purified by flash column chromatography (9:1 cyclohexane / EtOAc) to yield *tert*-butyl 3-((*tert*-butoxycarbonyloxy)methyl)-1*H*-indole-1-carboxylate **(30)** (5 mg, 0.013 mmol, 3%) as a colourless oil, and trace amounts of starting material **20**, *tert*-butyl 3-(hydroxymethyl)-1*H*-indole-1-carboxylate **(21)** and 1-allyl-1*H*-indole-3-carbaldehyde **(31)**. **21** and **31** possessed spectroscopic data that were consistent with those in the literature.[25]

***tert*-Butyl 3-((*tert*-butoxycarbonyloxy)methyl)-1*H*-indole-1-carboxylate (30)**

R_f = 0.2 (9:1 cyclohexane / EtOAc); ^{1}H NMR (500 MHz, $CDCl_3$) δ 1.41 (9H, s, C(C$\underline{H}_3$)$_3$), 1.59 (9H, s, C(C$\underline{H}_3$)$_3$), 5.18 (2H, d, J = 8 Hz, C$\underline{H}_2$-10), 7.19 (1H, ddd, J = 1, 7, 8 Hz, H-5), 7.26 (1H, ddd, J = 1, 7, 8 Hz, H-6), 7.56 (1H, d, J = 8 Hz, H-4), 7.59 (1H, s, H-2), 8.07 (1H, br d, J = 8 Hz, H-7); ^{13}C NMR (126 MHz, $CDCl_3$) δ 27.9 (C($\underline{C}$H$_3$)$_3$, 28.2 (C($\underline{C}$H$_3$)$_3$, 60.4 ($\underline{C}$H$_2$-10), 82.2 ($\underline{C}$(CH$_3$)$_3$), 83.9 ($\underline{C}$(CH$_3$)$_3$), 115.3 (C-7), 115.4 (C-3), 119.3 (C-4), 122.8 (C-5), 124.7 (C-6), 125.8 (C-2), 129.3 (C-8), 135.6 (C-9), 153.6 (O$\underline{C}$O*t*-Bu); IR (oil) 1732 (C=O), 1452, 767, 744; LCMS (Fast4min) t_r = 3.48 min, *m/z* 370 [M + Na]$^+$, purity (AUC) = 93%; HRMS (ESI) *m/z* calcd for $C_{19}H_{25}NNaO_5$ [M + Na]$^+$ 370.1625, found [M + Na]$^+$ 370.1629.

1-Allyl-1*H*-indole-3-carbaldehyde (31)

R_f = 0.6 (1:1 cyclohexane / EtOAc); ^{1}H NMR (500 MHz, $CDCl_3$) δ 4.80 (2H, ddd, *J* = 2, 2, 6 Hz, CH_2-10), 5.21 (1H, ddt, *J* = 2, 19, 2 Hz, H-12b), 5.33 (1H, ddt, *J* = 2, 11, 2, H-12a), 6.04 (1H, ddt, *J* = 11, 19, 6 Hz, H-11), 7.32-7.38 (3H, m, H-5, H-6, H-7), 7.73 (1H, s, H-2), 8.31-8.33 (1H, m, H-4), 10.03 (1H, s, CHO); IR (oil) 1655 (C=O), 1610 (C=C), 930 (C=C), 742; LCMS (Fast4min) t_r = 2.54 min, *m/z* 186 $[M + H]^+$, purity (AUC) = 78%; HRMS (ESI) *m/z* calcd for $C_{12}H_{11}NNaO$ $[M + Na]^+$ 208.0733, found $[M + Na]^+$ 208.0735.

tert-Butyl 3-((allyloxycarbonyloxy)methyl)-1*H*-indole-1-carboxylate (32)

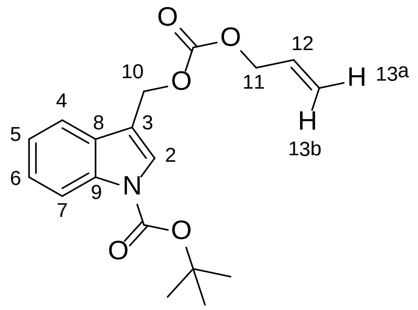

A solution of *tert*-butyl 3-(hydroxymethyl)-1*H*-indole-1-carboxylate **(21)** (100 mg, 0.405 mmol) and pyridine (41 µL, 0.51 mmol) in THF (2.3 mL) was cooled to 0 °C and a solution of allyl chloroformate (54 µL, 0.51 mmol) in THF (0.3 mL) was added dropwise. The reaction mixture was allowed to warm to room temperature and stirred for 7 h. The mixture was filtered and the filtrate concentrated *in vacuo*. The residue was taken up in Et_2O (20 mL), filtered again, and the filtrate washed with water (20 mL), brine (20 mL), dried ($MgSO_4$) and concentrated *in vacuo*. The crude material was purified by flash column chromatography (9:1 cyclohexane / EtOAc) to yield the title compound (83 mg, 0.25 mmol, 61%) as a yellow oil.

R_f = 0.2 (9:1 cyclohexane / EtOAc); ^{1}H NMR (500 MHz, $CDCl_3$) δ 1.67 (s, 9H, $C(CH_3)_3$), 4.64 (ddd, 2H, *J* = 1, 2, 6 Hz, CH_2-11), 5.26 (1H, ddt, *J* = 2, 11, 1 Hz, H-13a), 5.34 (2H, d, *J* = 1 Hz, CH_2-10), 5.35 (1H, ddt, *J* = 2, 20, 2 Hz, H-13b), 5.93 (1H, ddt, *J* = 11, 20, 6, H-12), 7.27 (1H, ddd, *J* = 1, 8, 8 Hz, H-5), 7.35 (1H, ddd, *J* = 1, 8, 8 Hz, H-6), 7.65 (1H, d, *J* = 8 Hz, H-4), 7.69 (1H, s, H-2), 8.15 (1H, br d, *J* = 8 Hz, H-7); ^{13}C NMR (126 MHz, $CDCl_3$) δ 28.3 ($C(CH_3)_3$), 61.7 (C-10), 68.7 (C-11), 84.2($C(CH_3)_3$), 115.1 (C-3), 115.5 (C-7), 119.0 (C-13), 119.4 (C-4), 123.1 (C-5), 124.9 (C-6), 126.2 (C-2), 129.3 (C-8), 131.7 (C-12), 135.8 (C-9), 149.6 (NCO), 155.2 (CO_3); IR (oil) 1733 (C=O), 1451, 835, 767, 744; LCMS (Fast4min) t_r =

3.37 min, *m/z* 354 $[M + H]^+$, purity (AUC) > 95%; HRMS (ESI) *m/z* calcd for $C_{18}H_{21}NNaO_5$ $[M + Na]^+$ 354.1312, found $[M + Na]^+$ 354.1321.

(*S*)-3-(1-Allyl-1*H*-indol-3-yl)-2-aminopropanoic acid (33)

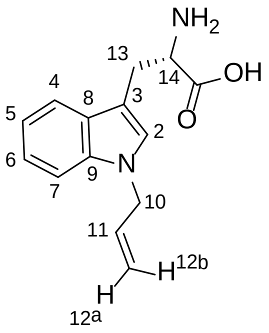

To a suspension of L-tryptophan (204 mg, 1.00 mmol) in DMF (5 mL) was added NaH (60% in mineral oil, 100 mg, 2.50 mmol) and the mixture stirred at room temperature for 30 min. Allyl bromide (90 µL, 1.0 mmol) was added dropwise and the mixture stirred for a further 6 h. The reaction was diluted with water (50 mL) and EtOAc (50 mL), the layers separated and the aqueous layer concentrated *in vacuo*. A 50 mg sample of the 353 mg crude material was purified by reversed-phase column chromatography (gradient elution 0-40% MeCN in water) to yield the title compound (14 mg, 0.057 mmol, estimated 40%) as a white solid which possessed spectroscopic data that were consistent with those in the literature.[26]

m.p: 221 °C, lit[26] 203-204 °C; R_f = 0.4 (2:3:8 water / propan-2-ol / EtOAc); ^{1}H NMR (500 MHz, MeOD) δ 3.15 (dd, 1H, *J* = 9, 15 Hz, C$\underline{H}_2$-13a), 3.49 (dd, 1H, *J* = 4, 15 Hz, C$\underline{H}_2$-13b), 3.84 (dd, 1H, *J* = 4, 9 Hz, H-14), 4.75-4.77 (m, 2H, C$\underline{H}_2$-10), 5.10 (ddt, 1H, *J* = 2, 17, 2 Hz, H-12b), 5.18 (ddt, 1H, *J* = 2, 12, 1 Hz, H-12a), 6.03 (ddt, 1H, *J* = 12, 17, 5 Hz, H-11), 7.08 (dd, 1H, *J* = 7, 8 Hz, H-5), 7.16 (s, 1H, H-2), 7.17 (dd, 1H, *J* = 7, 8 Hz, H-6), 7.34 (d, 1H, *J* = 8 Hz, H-7), 7.72 (d, 1H, *J* = 8 Hz, H-4); ^{13}C NMR (126 MHz, MeOD) δ 28.6 (C-13), 49.6 (C-10), 56.8 (C-14), 109.7 (C-3), 110.9 (C-7), 117.3 (C-12), 119.8 (C-4), 120.3 (C-5), 122.9 (C-

6), 128.6 (C-2), 129.3 (C-8), 135.3 (C-11), 138.3 (C-9), 174.9 ($\underline{C}O_2H$); $[\alpha]_D^{22}$ = -16 (*c* 0.5, MeOH); IR (solid) 2915 (O-H), 1584 (N-H), 1468, 1310 (O-H), 990, 909, 740; LCMS (Fast4min) t_r = 2.07 min, *m/z* 228 $[M – OH + H]^+$, purity (AUC) = 95%; HRMS (ESI) *m/z* calcd for $C_{14}H_{17}N_2O_2$ $[M + H]^+$ 245.1285, found $[M + H]^+$ 245.1286.

(3*R*,7a*R*)-7a-Allyl-3-(trichloromethyl)tetrahydropyrrolo[1,2-*c*]oxazol-1(3*H*)-one (34)

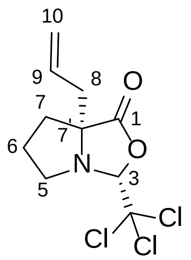

A solution of diisopropylamine (3.53 mL, 25.0 mmol) in THF (50 mL) was cooled to -78 °C and *n*-BuLi (2.5 M in hexanes, 10.0 mL, 25.0 mmol) was added dropwise. The solution was stirred for 5 min, warmed to 0 °C and stirred for 15 min. A solution of (3*R*,7a*S*)-3-(trichloromethyl)tetrahydropyrrolo[1,2-*c*]oxazol-1-(3*H*)-one (4.08 g, 16.7 mmol) in THF (75 mL) was cooled to -78 °C and the freshly-prepared LDA solution added dropwise over 20 min. After stirring at -78 °C for a further 30 min, allyl bromide (4.33 mL, 50.0 mmol) was added dropwise over 5 min. The mixture was stirred at -78 °C for 3 h, then the reaction was quenched with water (50 mL), warmed to room temperature and the THF removed *in vacuo*. The mixture was extracted with $CHCl_3$ (3 × 25 mL), and the combined organic extracts dried ($MgSO_4$) and concentrated *in vacuo* to yield the title compound (3.55 g, 12.5 mmol, 75%) as an orange oil which possessed spectroscopic data that were consistent with those in the literature.[11]

R_f = 0.2 (9:1 cyclohexane / EtOAc); ^{1}H NMR (500 MHz, $CDCl_3$) δ 1.61-1.71 (m, 1H, $C\underline{H}_2$-6a), 1.87-1.93 (m, 1H, $C\underline{H}_2$-6b), 1.99-2.05 (m, 1H, $C\underline{H}_2$-7a), 2.11-2.16 (m, 1H, $C\underline{H}_2$-7b), 2.55 (dd, 1H, *J* = 8, 14 Hz, $C\underline{H}_2$-8a), 2.62 (dddd, 1H, *J* = 1, 1, 7, 14 Hz, $C\underline{H}_2$-8b), 3.15-3.25 (m, 2H,

C$\underline{H}_2$-5), 4.98 (s, 1H, H-3), 5.16-5.20 (m, 2H, C$\underline{H}_2$-10), 5.85-5.93 (m, 1H, H-9); ^{13}C NMR (126 MHz, $CDCl_3$) δ 25.2 (C-6), 35.2 (C-7), 41.6 (C-8), 58.4 (C-5), 71.3 (C-7'), 100.5 (C-$\underline{C}Cl_3$), 102.4 (C-3), 119.9 (C-10), 132.0 (C-9), 176.2 (C-1); $[\alpha]_D^{22}$ = +32 (*c* 2, $CHCl_3$), lit[164] $[\alpha]_D^{25}$ = +45, (*c* 2, $CHCl_3$); IR (oil) 1796 (C=O), 1188, 1103, 919 (C=C), 799 (C-Cl); LCMS (Fast4min) t_r = 3.14 min, *m/z* 284 $[M + H]^+$, purity (AUC) > 95%; HRMS (ESI) *m/z* calcd for $C_{10}H_{13}Cl_3NO_2$ $[M + H]^+$ 284.0006, found $[M + H]^+$ 284.0014.

(3*R*,7a*R*)-1-Oxo-3-(trichloromethyl)hexahydropyrrolo[1,2-c]oxazole-7a-carbaldehyde (35)

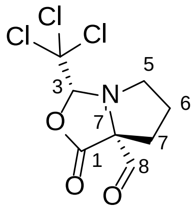

To a solution of diisopropylamine (4.6 mL, 33 mmol) in THF (23 mL) at -78 °C was added *n*-BuLi (2.5 M in hexanes, 13 mL, 33 mmol) dropwise. The solution was stirred for 5 min, warmed to 0 °C and stirred for 22 min. A solution of (3*R*,7a*S*)-3-(trichloromethyl)tetrahydropyrrolo[1,2-*c*]oxazol-1-(3*H*)-one (5.10 g, 20.9 mmol) in THF (25 mL) was cooled to -78 °C, and the freshly-prepared LDA solution (38.5 mL, 31.3 mmol) was added over 2 min. After stirring at -78 °C for 30 min, methyl formate (5.1 mL, 83) was added over 5 min and the mixture was warmed to -40 °C over 1 h. Citric acid (10% in H_2O, 25 mL) was added and the aqueous layer was extracted with Et_2O (2 × 25 mL). The combined organic layers were washed with brine (50 mL), dried ($MgSO_4$) and concentrated *in vacuo*. The crude material was purified by flash column chromatography (12-100% EtOAc in cyclohexane) to yield the title compound as an yellow semi- solid (2.20 g, 8.07 mmol, 39%) which possessed spectroscopic data that were consistent with those in the literature.[27]

R_f = 0.4 (1:1 EtOAc : cyclohexane); ^{1}H NMR (500 MHz, $CDCl_3$) δ 1.81-1.90 (1H, m, C$\underline{H}_2$-6a), 1.93-2.00 (1H, m, C$\underline{H}_2$-6b), 2.27-2.32 (1H, m, C$\underline{H}_2$-7a), 2.35-2.40 (1H, m, C$\underline{H}_2$-7b), 3.32-3.37 (1H, m, C$\underline{H}_2$-5), 3.51-3.56 (1H, m, C$\underline{H}_2$-5), 5.21 (1H, s, H-3), 9.59 (1H, s, H-8); ^{13}C NMR (126 MHz, $CDCl_3$) δ 25.5 (C-6), 33.9 (C-7), 59.0 (C-5), 78.2 (C-7'), 100.0 ($\underline{C}Cl_3$), 102.3 (C-3), 169.3 (C-1), 193.6 (C-8); $[\alpha]_D^{24}$ = +9 (*c* 1, MeOH), lit $[\alpha]_D^{25}$ = +30 (*c* 2, $CHCl_3$); LCMS

(Fast4min) t_r = 2.47 min, *m/z* 272 [M + H]$^+$; purity (AUC) = 94%; HRMS (ESI) *m/z* calcd for $C_8H_9Cl_3NO_3$ [M + H]$^+$ 271.9643, found [M + H]$^+$ 271.9640.

(3*R*,7a*R*)-3-(Trichloromethyl)-7a-vinyltetrahydropyrrolo[1,2-c]oxazol-1(3*H*)-one (36)

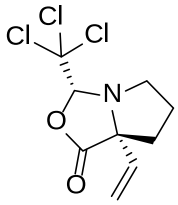

$MePPh_3Br$ (786 mg, 2.20 mmol) and KO*t*-Bu (247 mg, 2.20 mmol) were suspended in toluene (40 mL) and stirred for 2 h at 80 °C. The mixture was cooled to room temperature and a solution of (3*R*,7a*R*)-1-oxo-3-(trichloromethyl)hexahydropyrrolo[1,2-c]oxazole-7a-carbaldehyde **(35)** (500 mg, 1.83 mmol) in toluene (8 mL) was added. The mixture was stirred at room temperature for 21.5 h, and then at 50 °C for 3.5 h. After cooling to room temperature, Et_2O (50 mL) was added, the precipitate was removed by filtration, and the filtrate was concentrated *in vacuo*. The crude material was subjected to flash column chromatography (12-100% EtOAc in cyclohexane) but product **36** could not be separated from starting material **35**.

HRMS (ESI) *m/z* calcd for $C_9H_{11}{}^{35}Cl_2{}^{37}ClNO_2$ [M + H]$^+$ 271.9821, found [M + H]$^+$ 271.9812.

NMR spectra

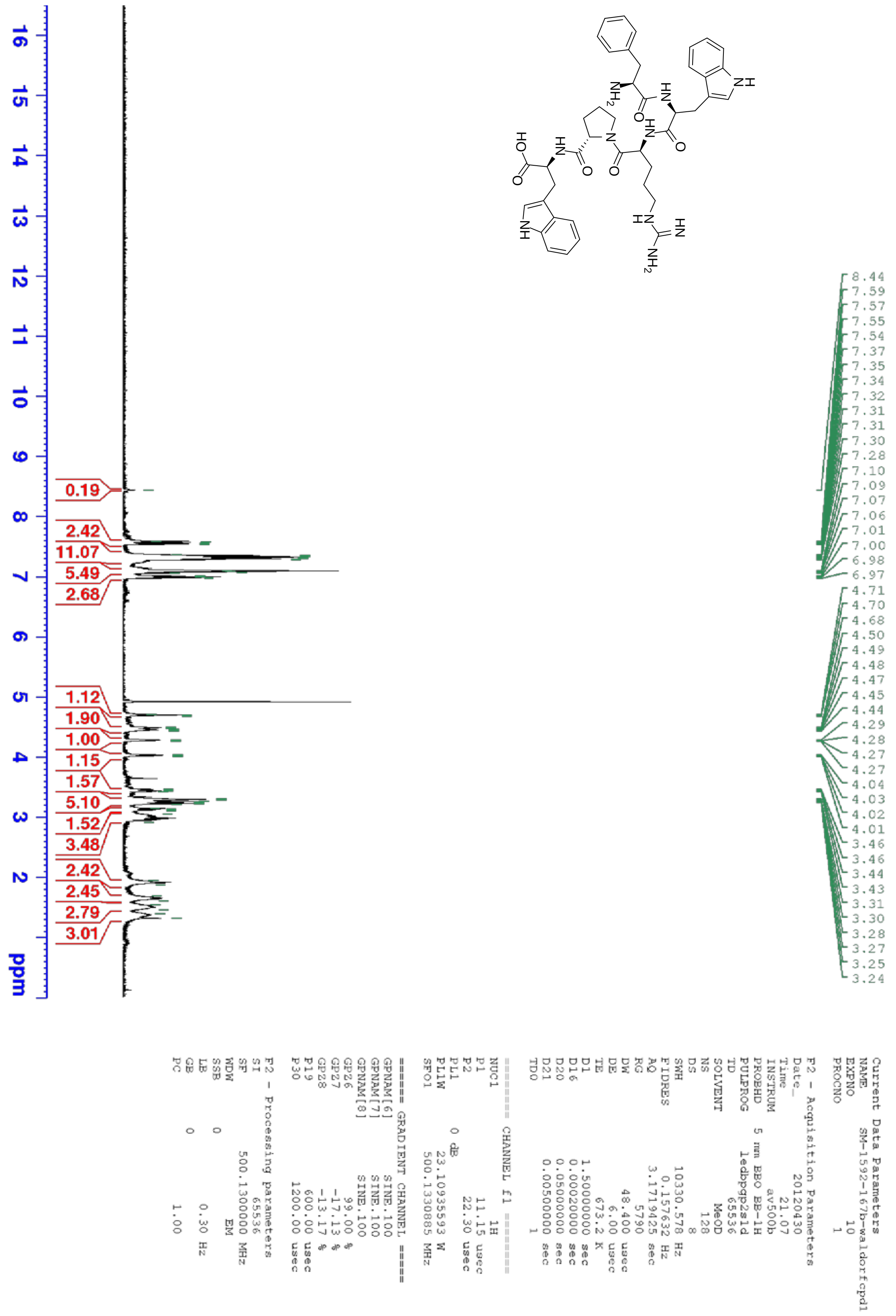

Figure S2. ^{1}H NMR (CD_3OD, 500 MHz) of **1**.

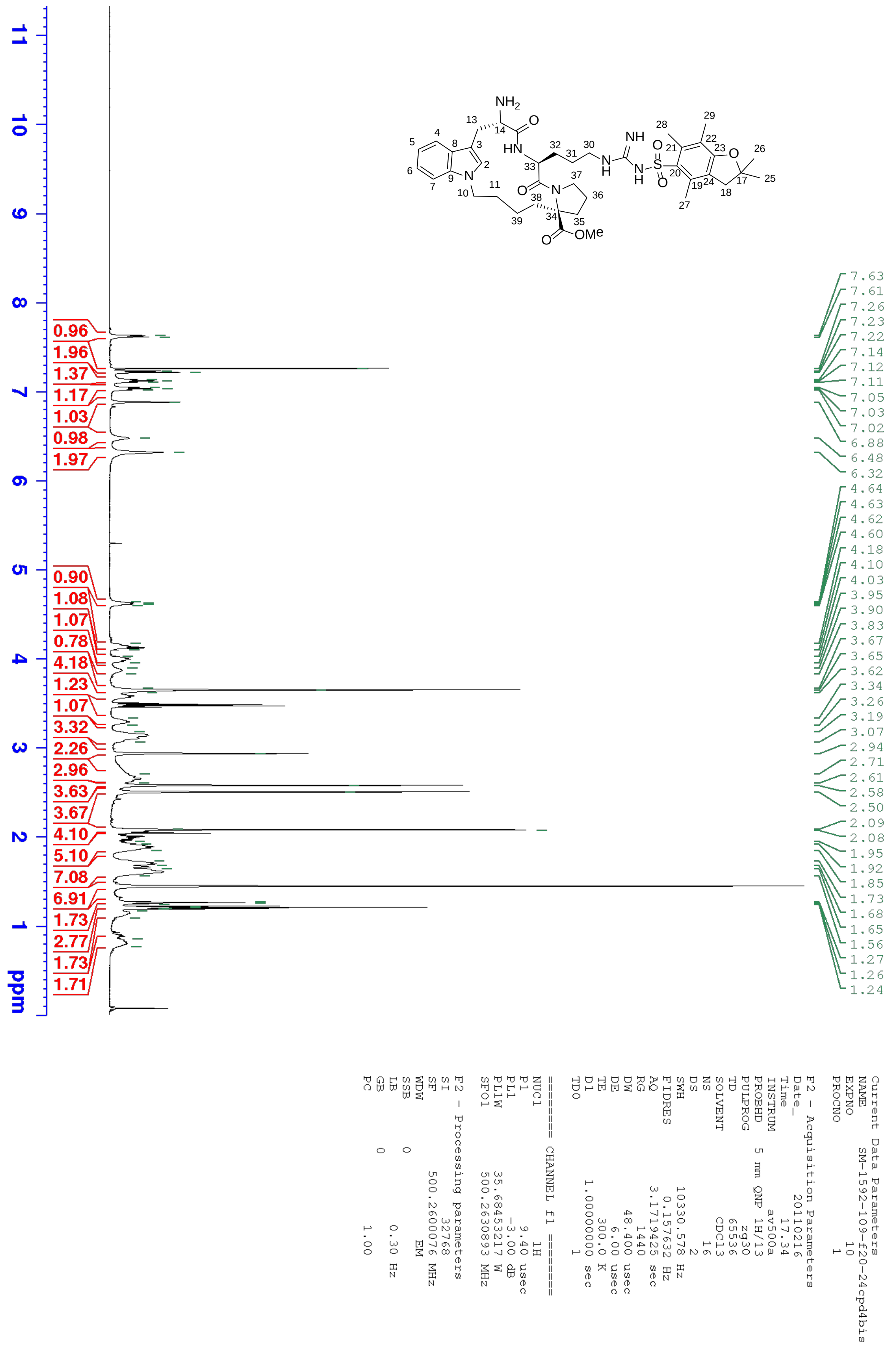

Figure S3. ^{1}H NMR ($CDCl_3$, 500 MHz) of **4**.

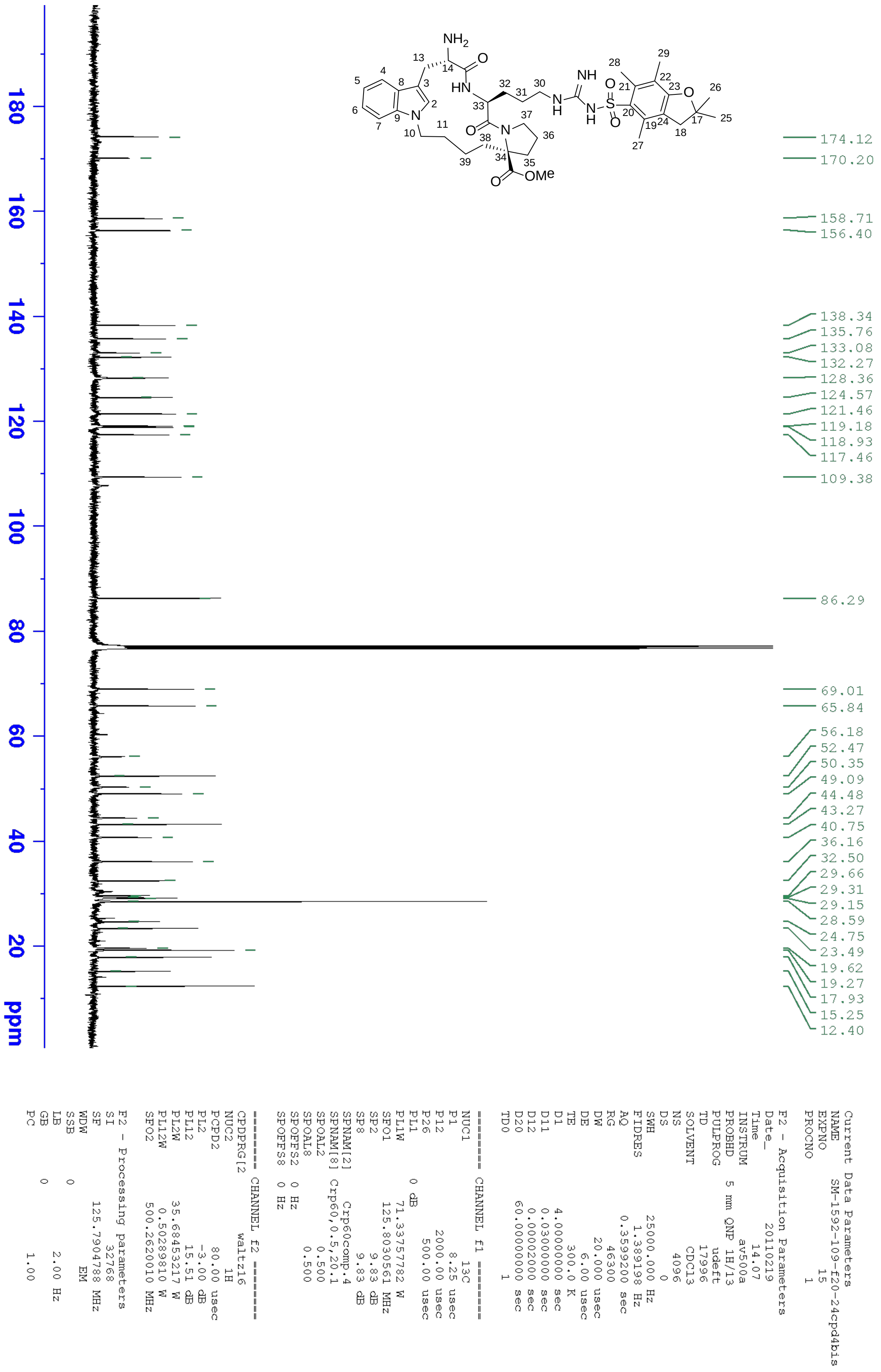

Figure S4. ^{13}C NMR ($CDCl_3$, 126 MHz) of **4**.

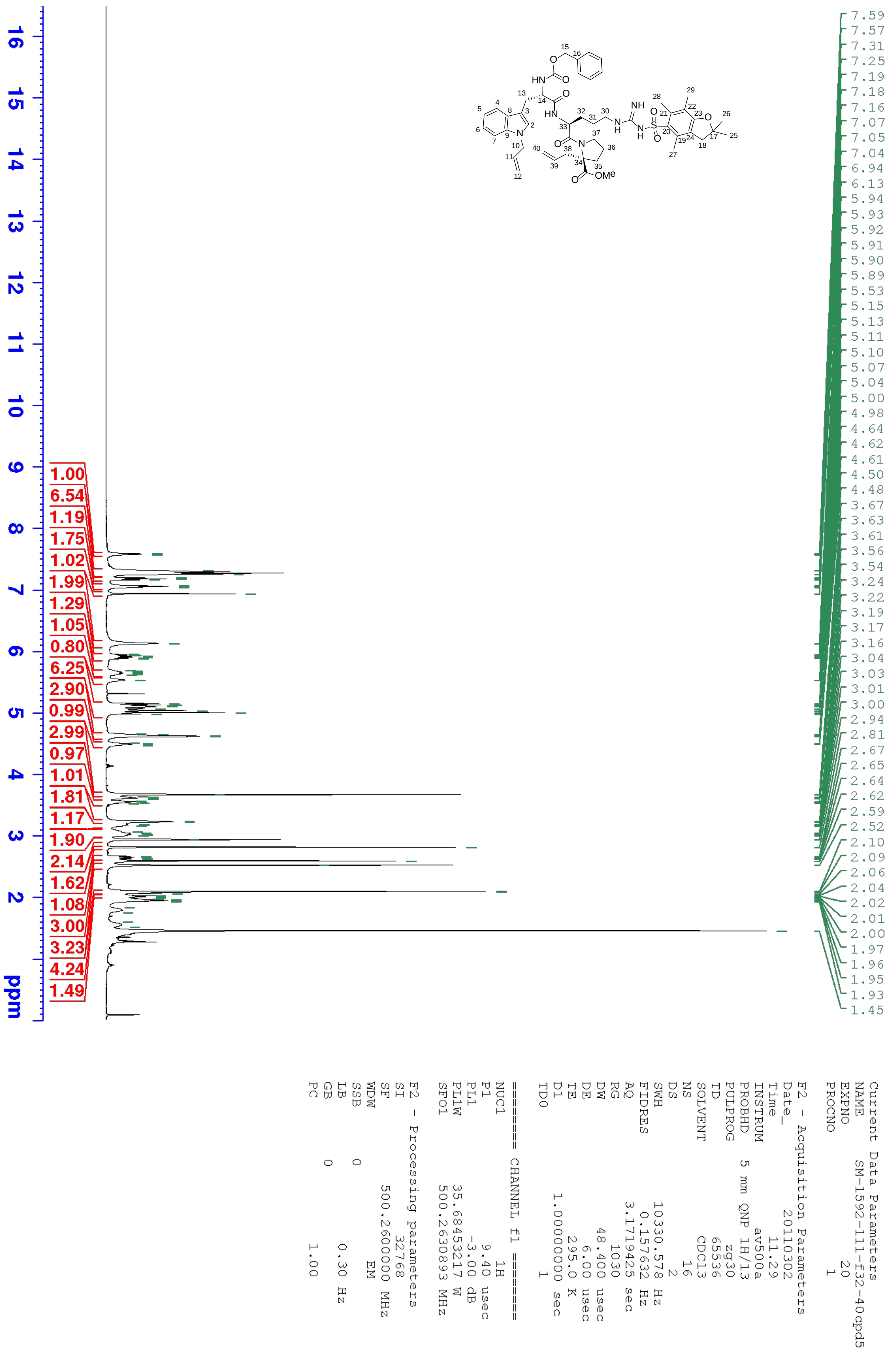

Figure S5. ^{1}H NMR ($CDCl_3$, 500 MHz) of **5**.

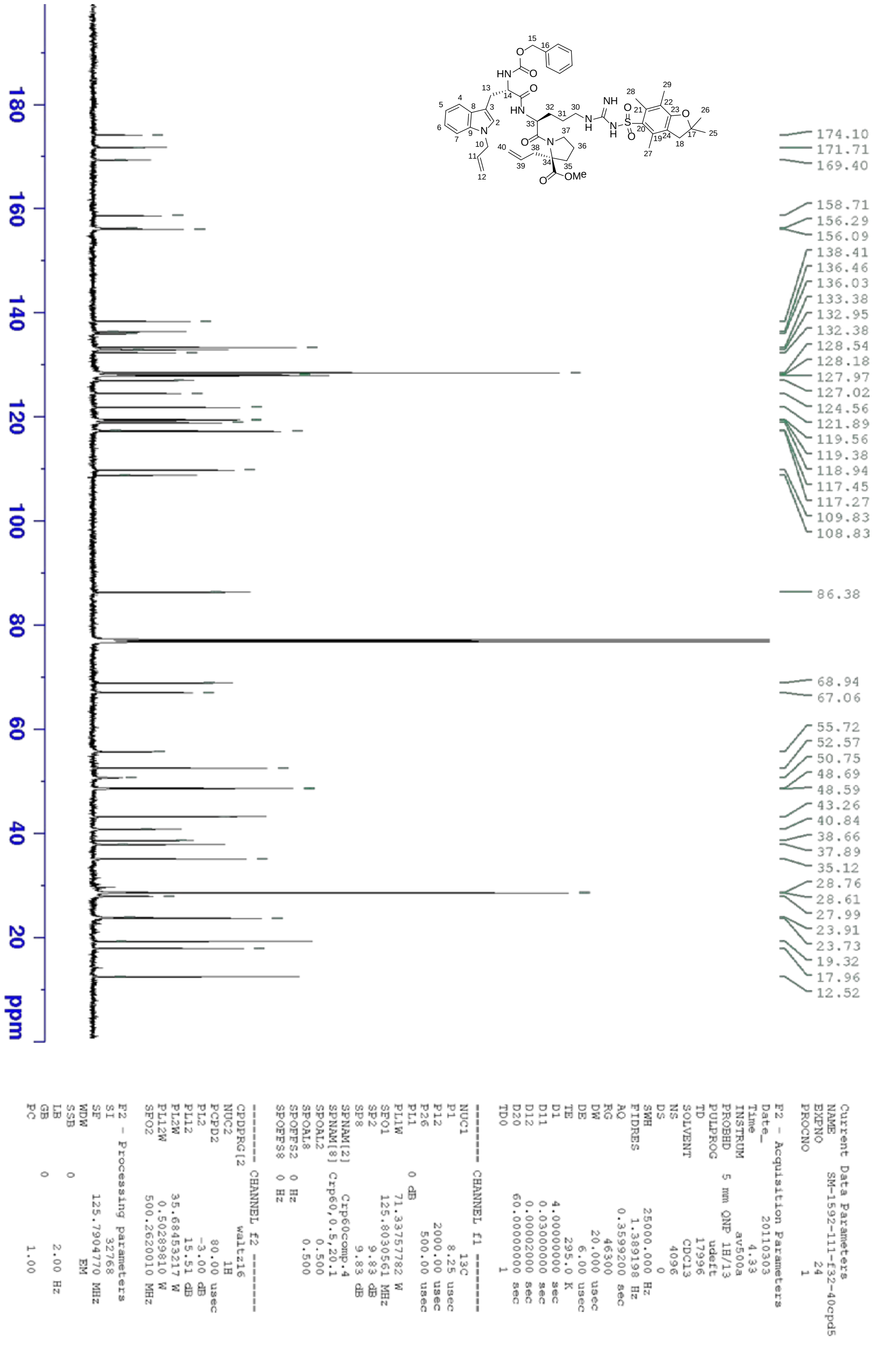

Figure S6. ^{13}C NMR ($CDCl_3$, 126 MHz) of **5**.

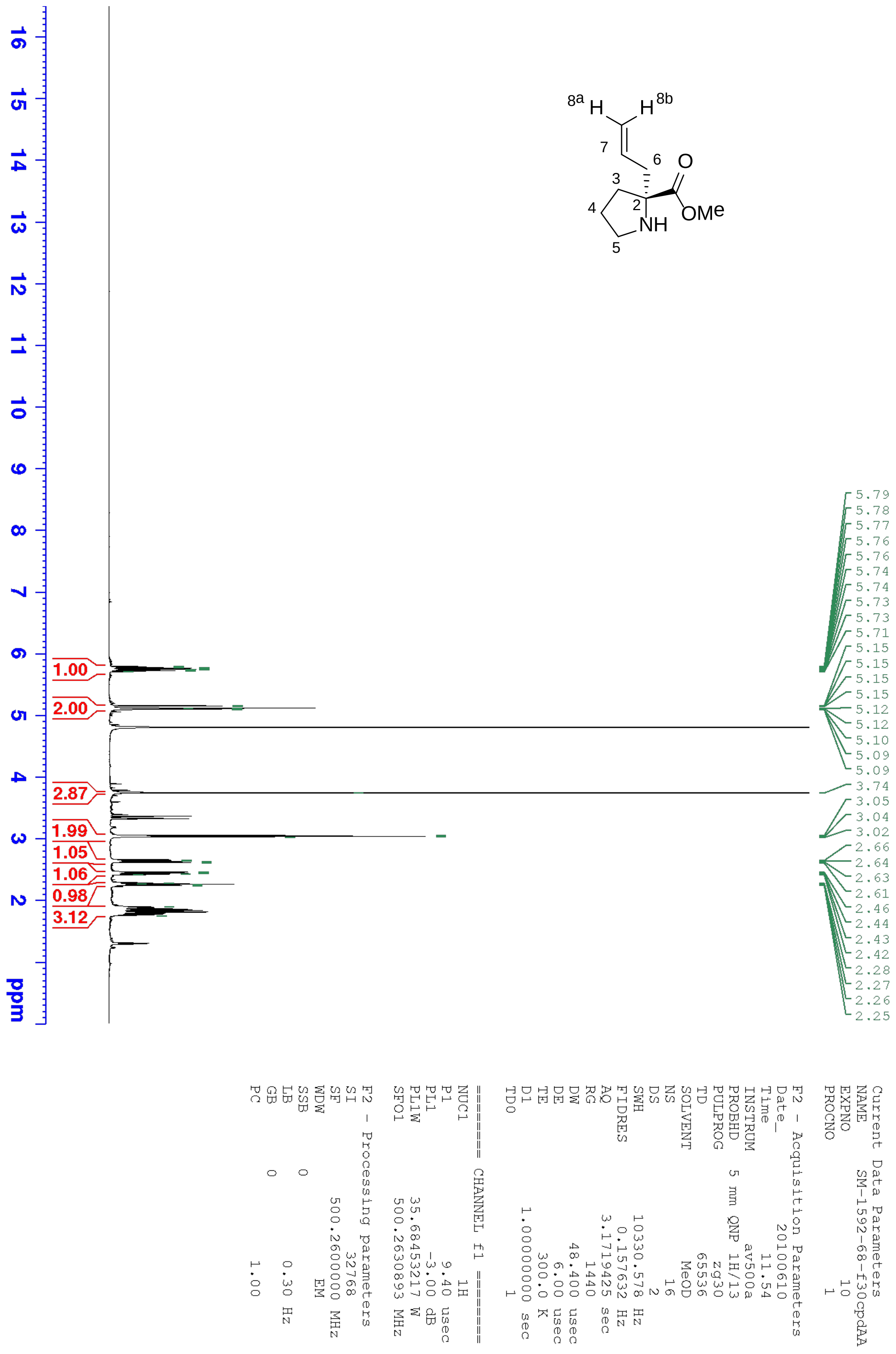

Figure S7. ^{1}H NMR (CD_3OD, 500 MHz) of **6**.

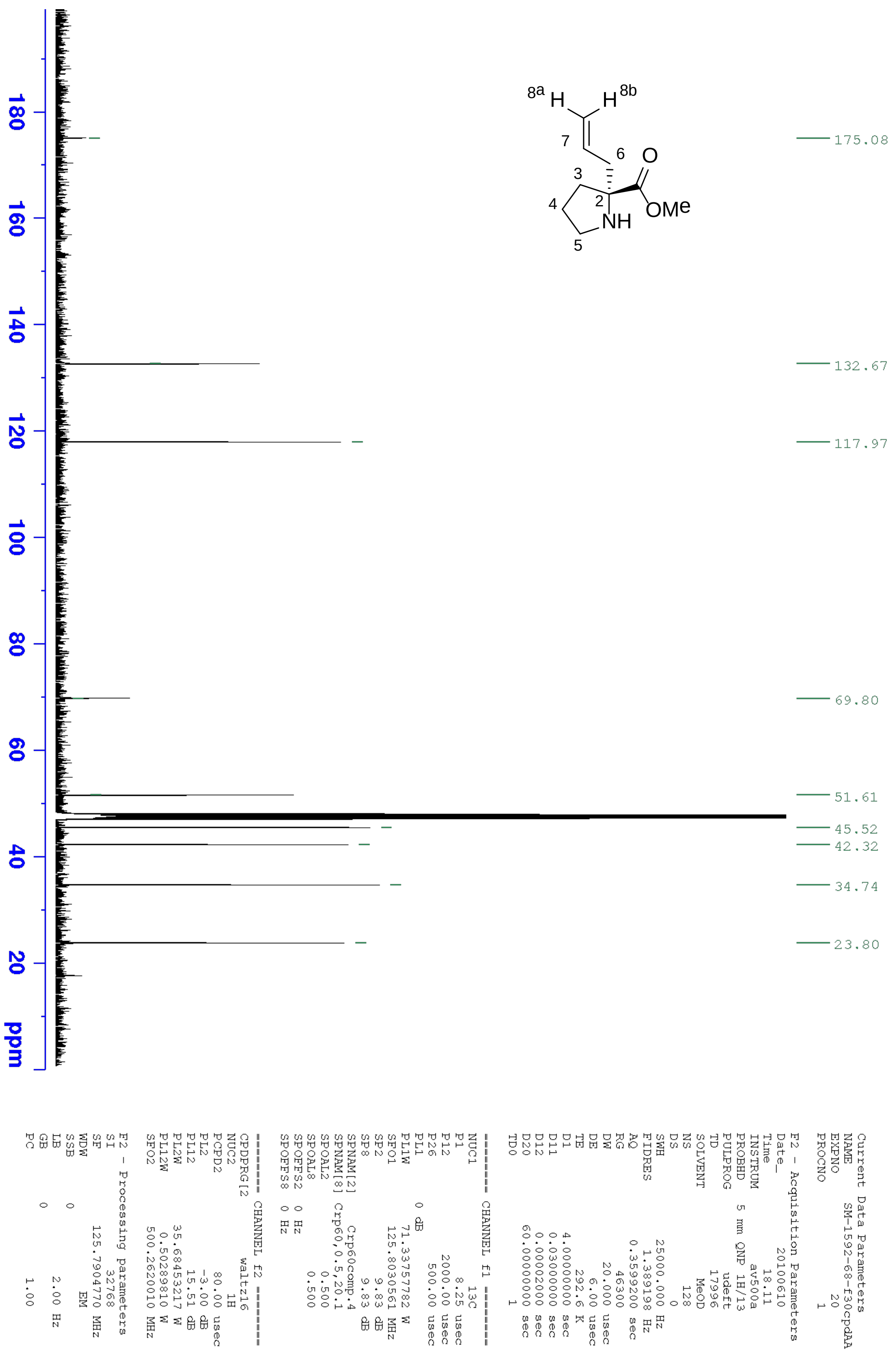

Figure S8. ^{13}C NMR (CD_3OD, 126 MHz) of **6**.

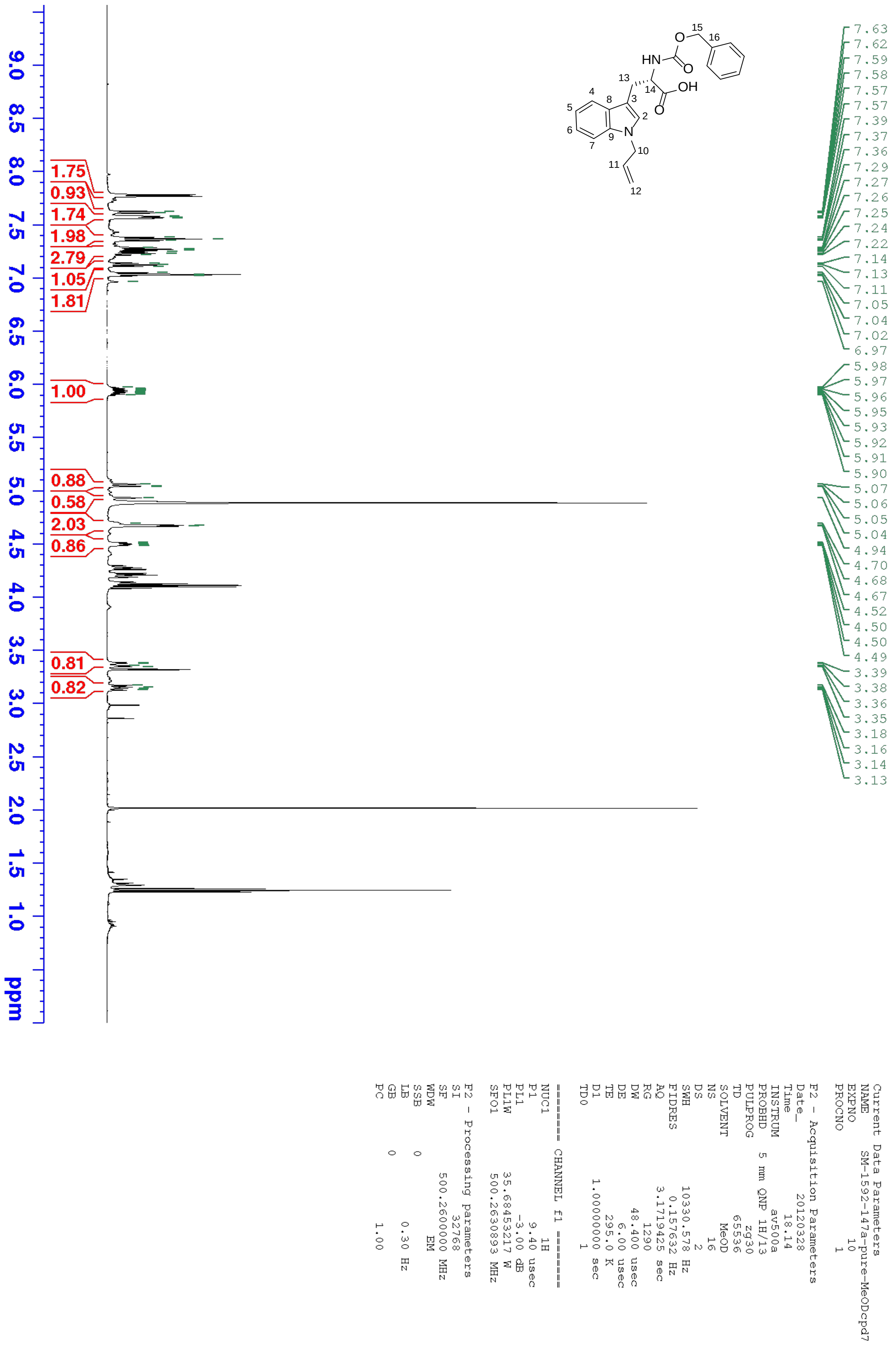

Figure S9. ^{1}H NMR (CD_3OD, 500 MHz) of **7**.

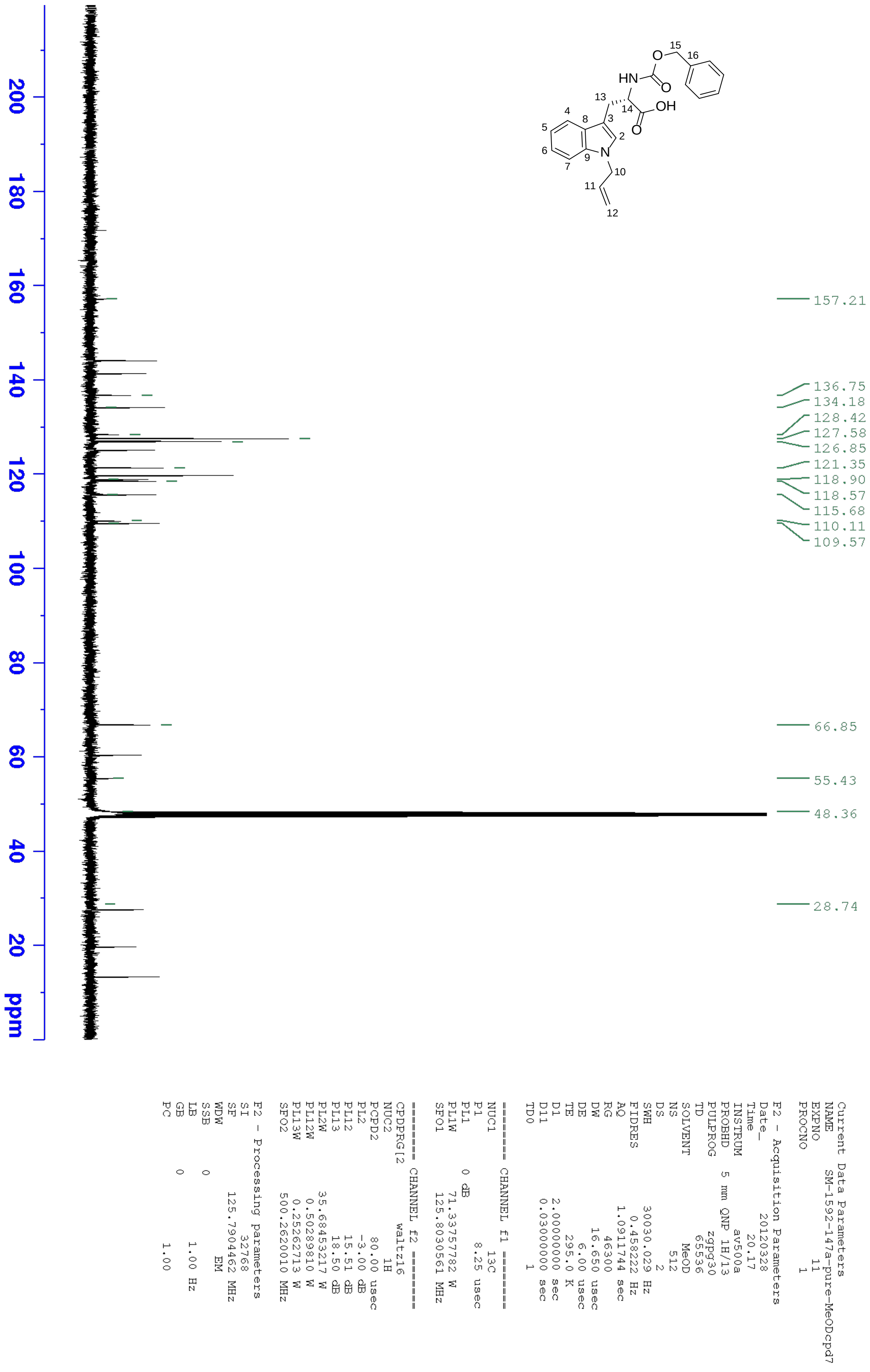

Figure S10. ^{13}C NMR (CD_3OD, 126 MHz) of **7**.

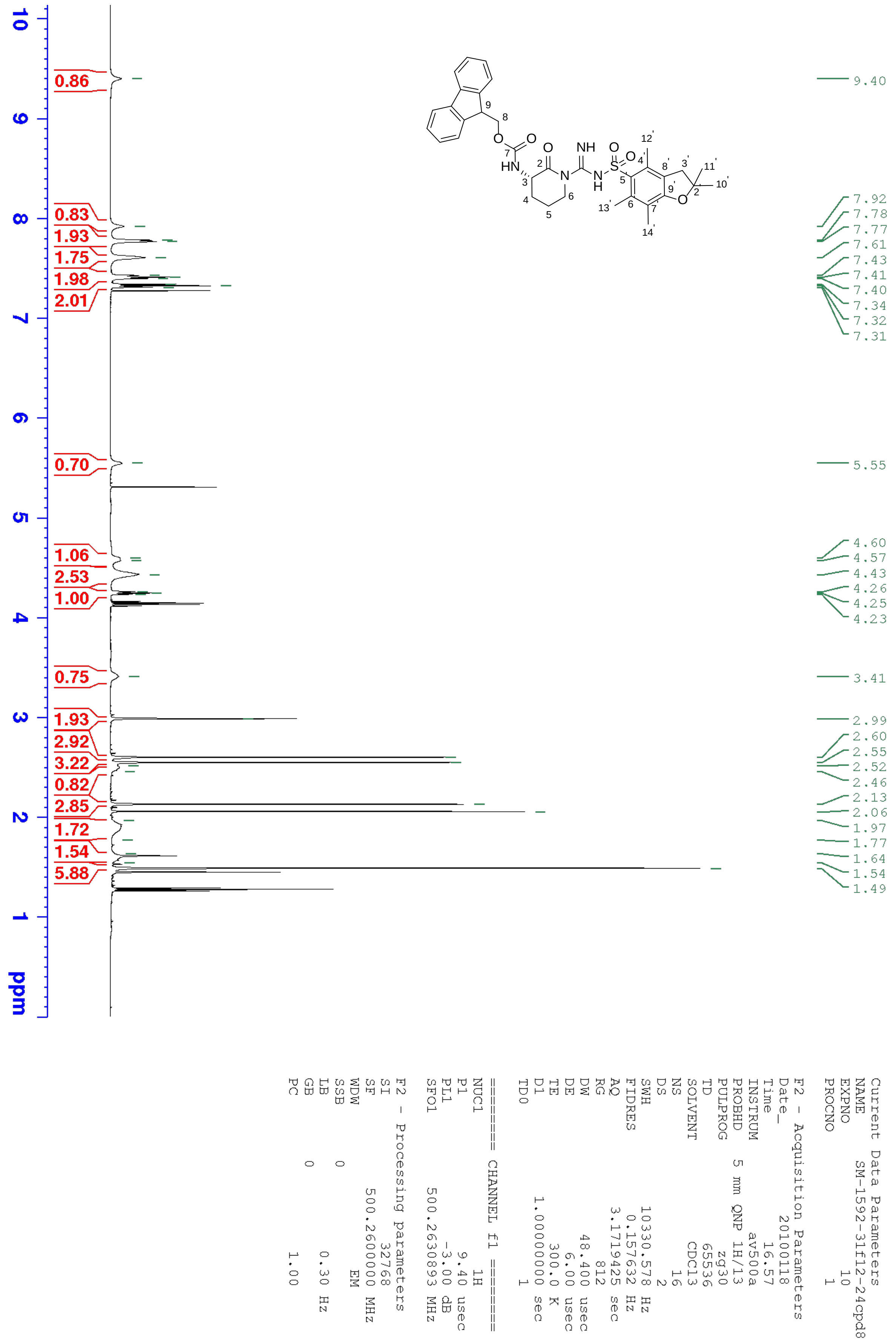

Figure S11. ^{1}H NMR ($CDCl_3$, 500 MHz) of **8**.

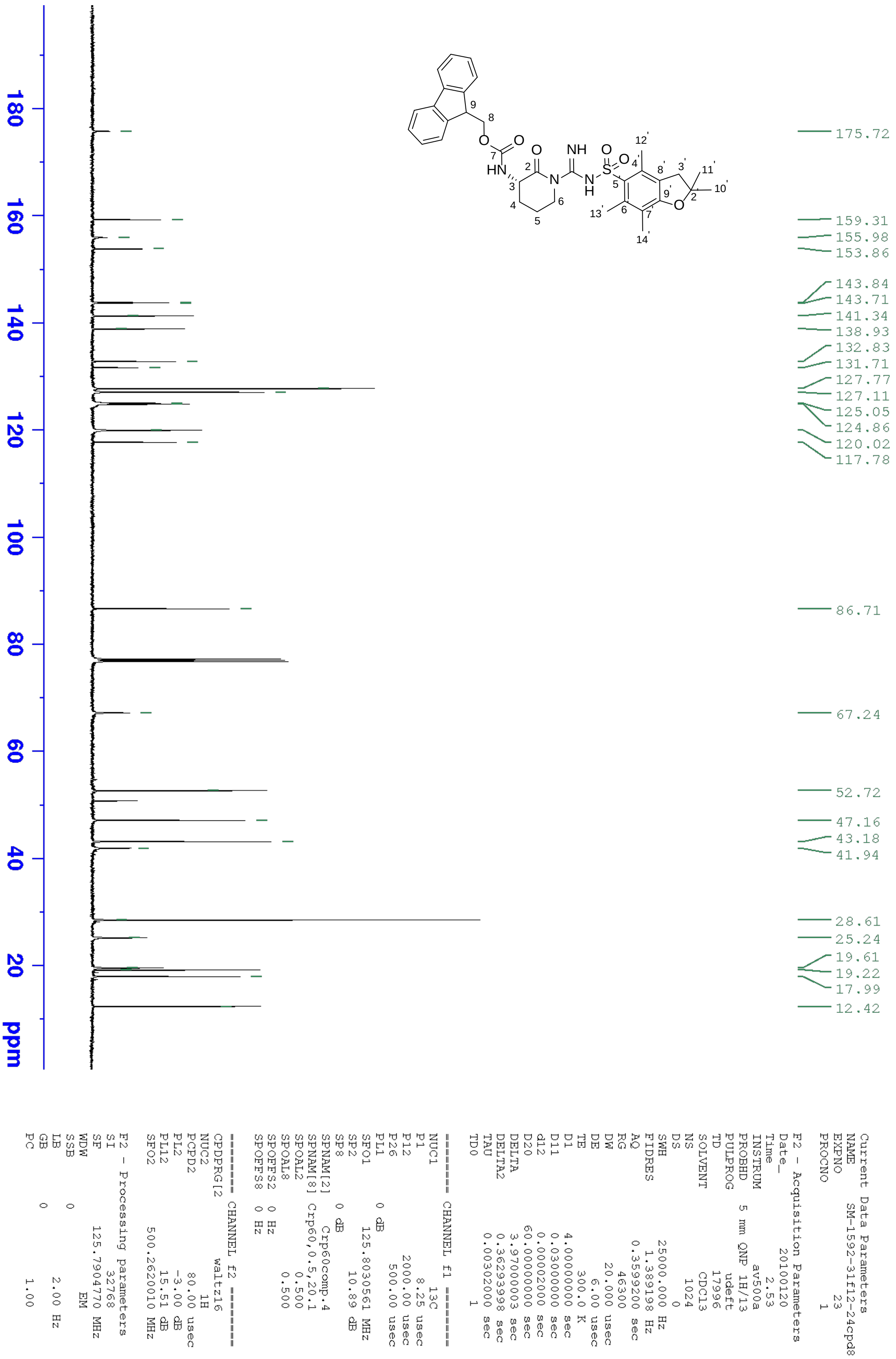

Figure S12. ^{13}C NMR ($CDCl_3$, 126 MHz) of **8**.

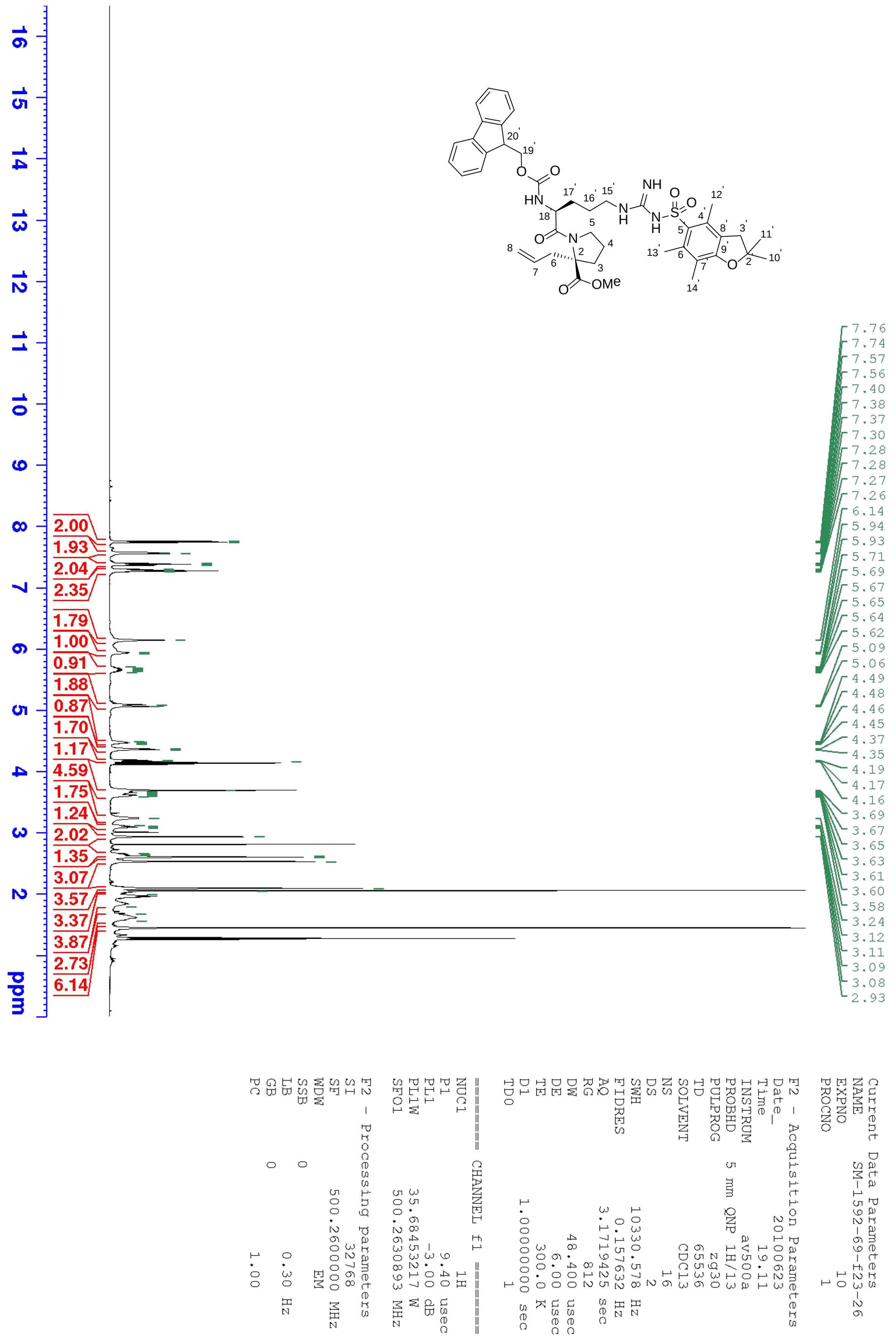

Figure S13. ^{1}H NMR ($CDCl_3$, 500 MHz) of **9**.

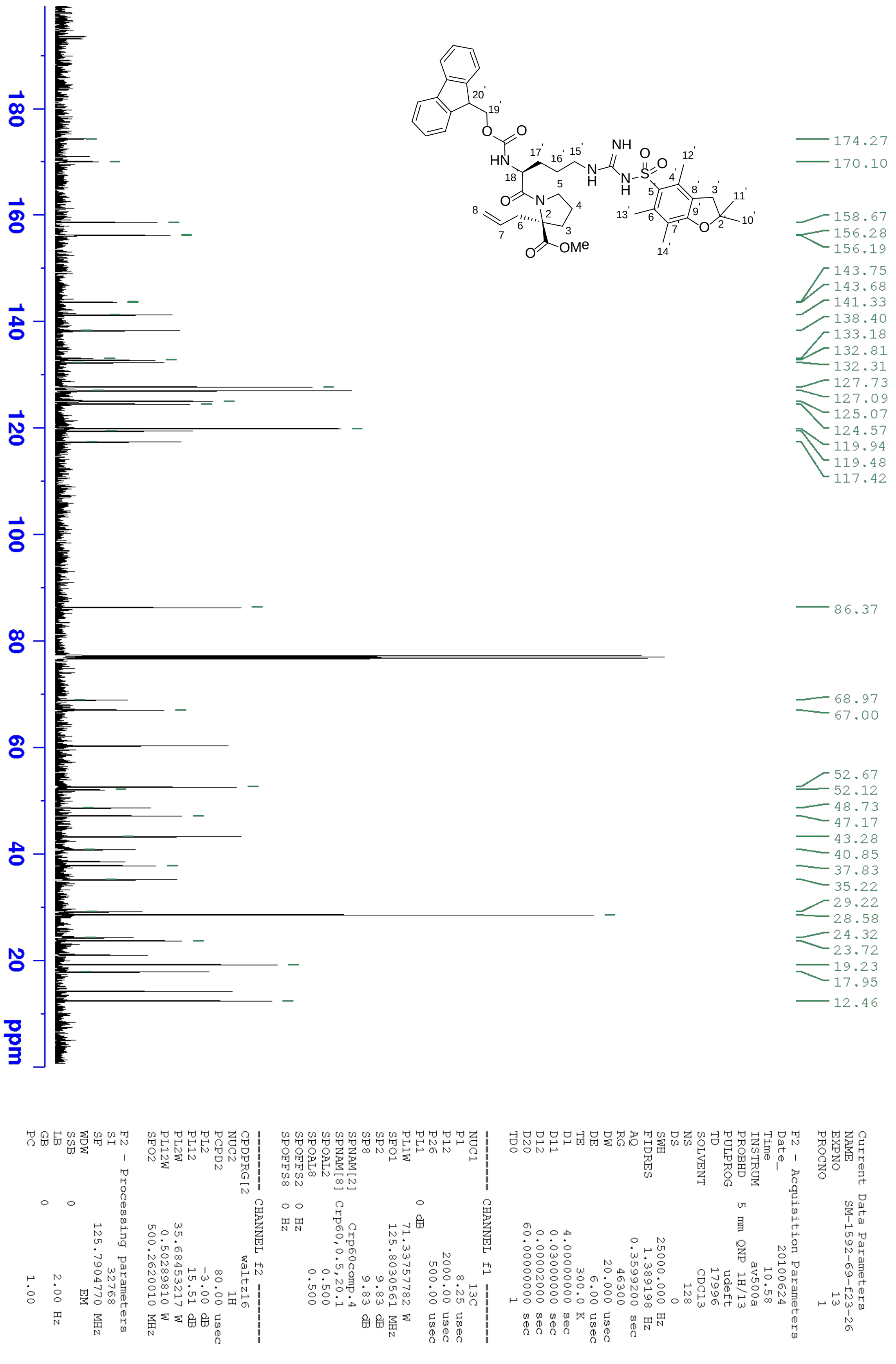

Figure S14. ^{13}C NMR ($CDCl_3$, 126 MHz) of **9**.

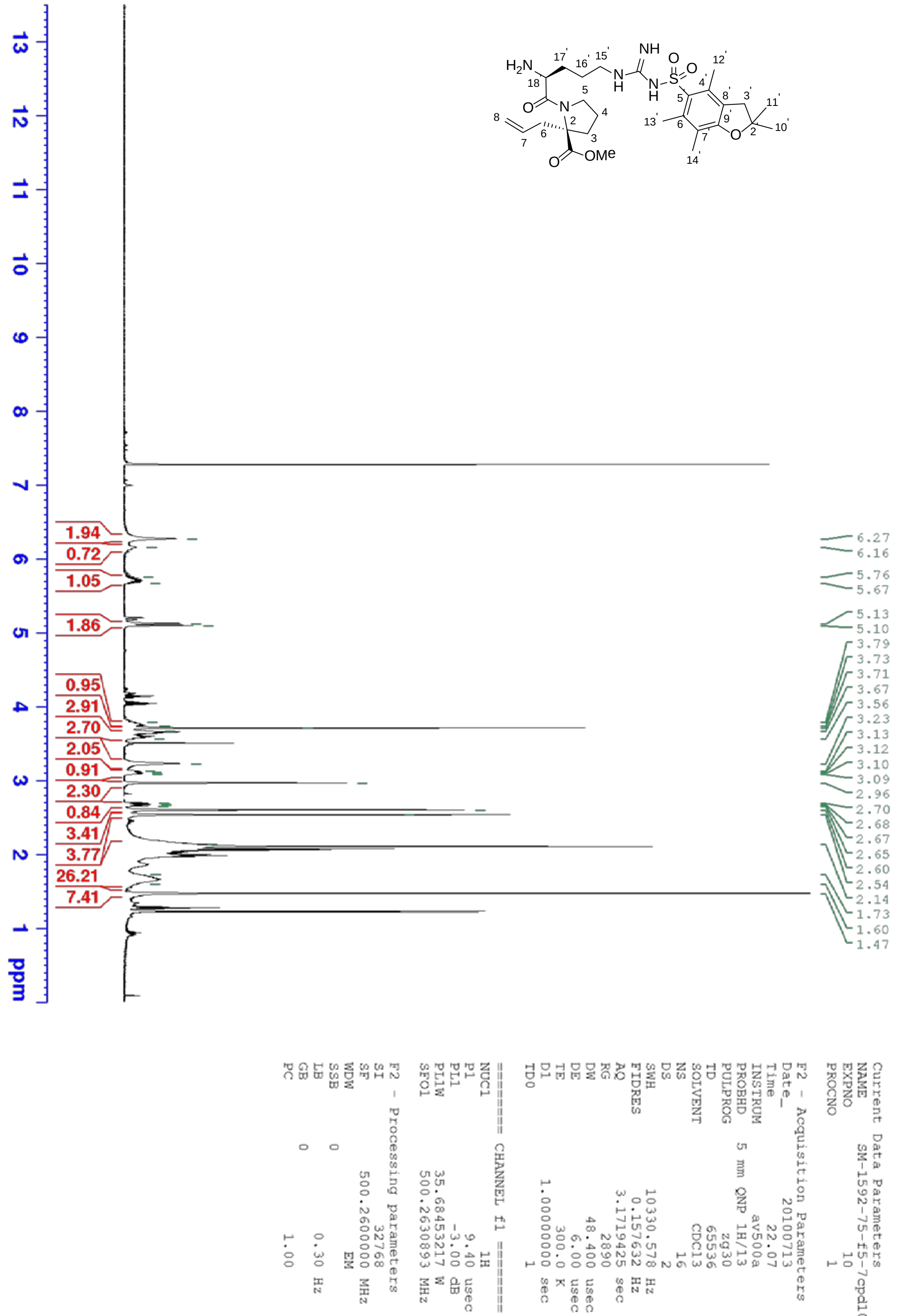

Figure S15. ^{1}H NMR ($CDCl_3$, 500 MHz) of **10**.

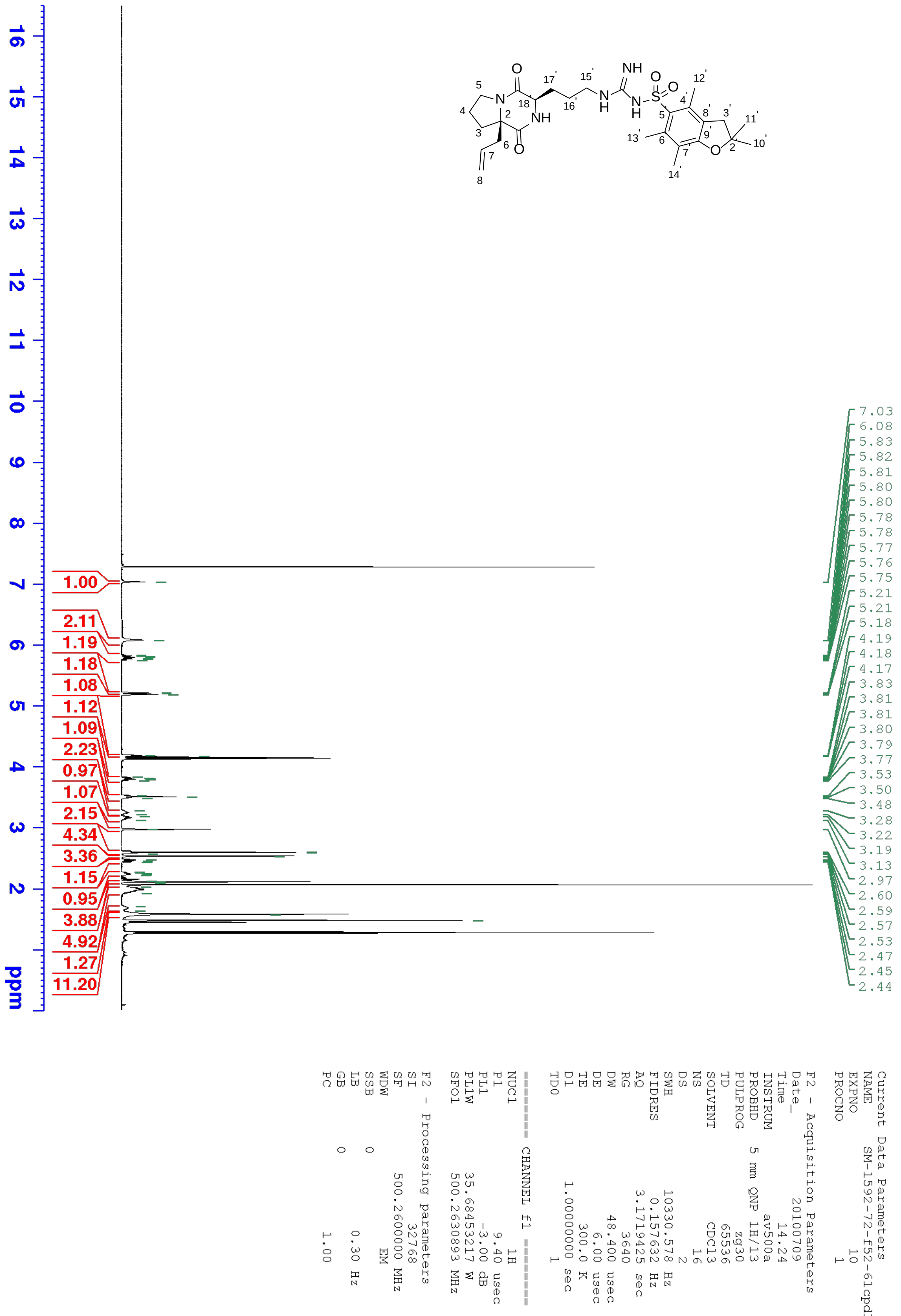

Figure S16. ^{1}H NMR ($CDCl_3$, 500 MHz) of **11**.

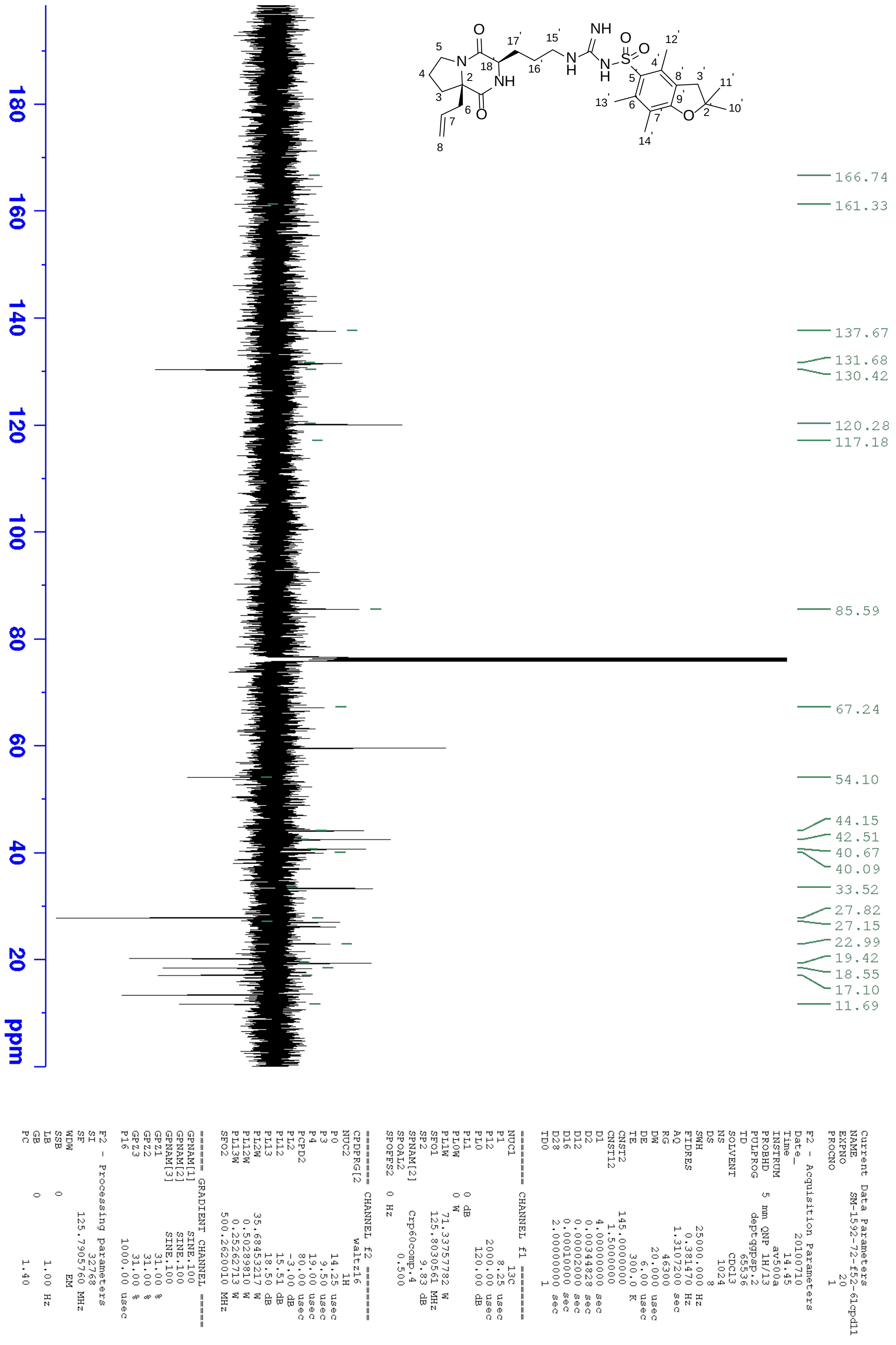

Figure S17. ^{13}C NMR ($CDCl_3$, 126 MHz) of **11**.

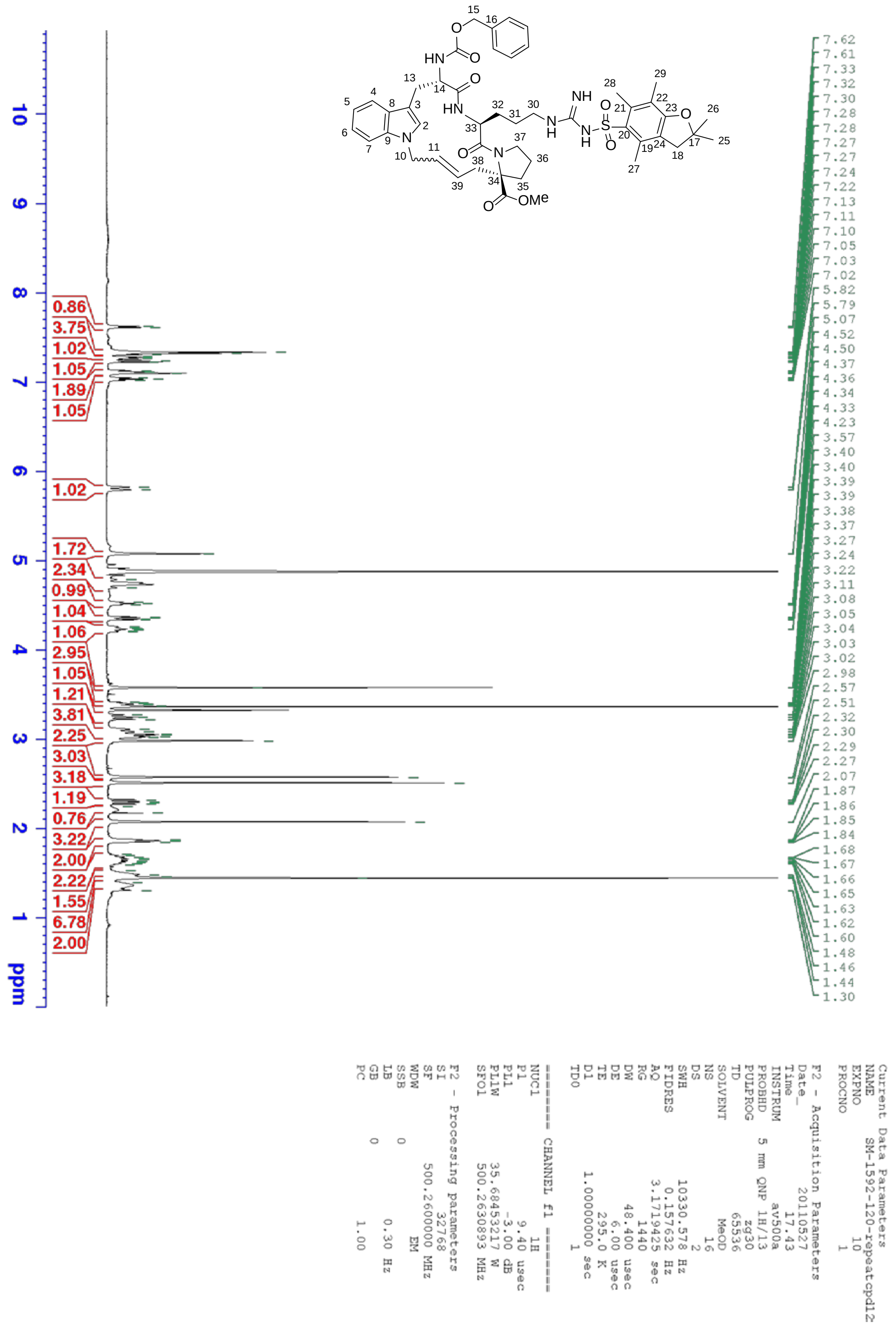

Figure S18. ^{1}H NMR (CD_3OD, 500 MHz) of **12 *trans***.

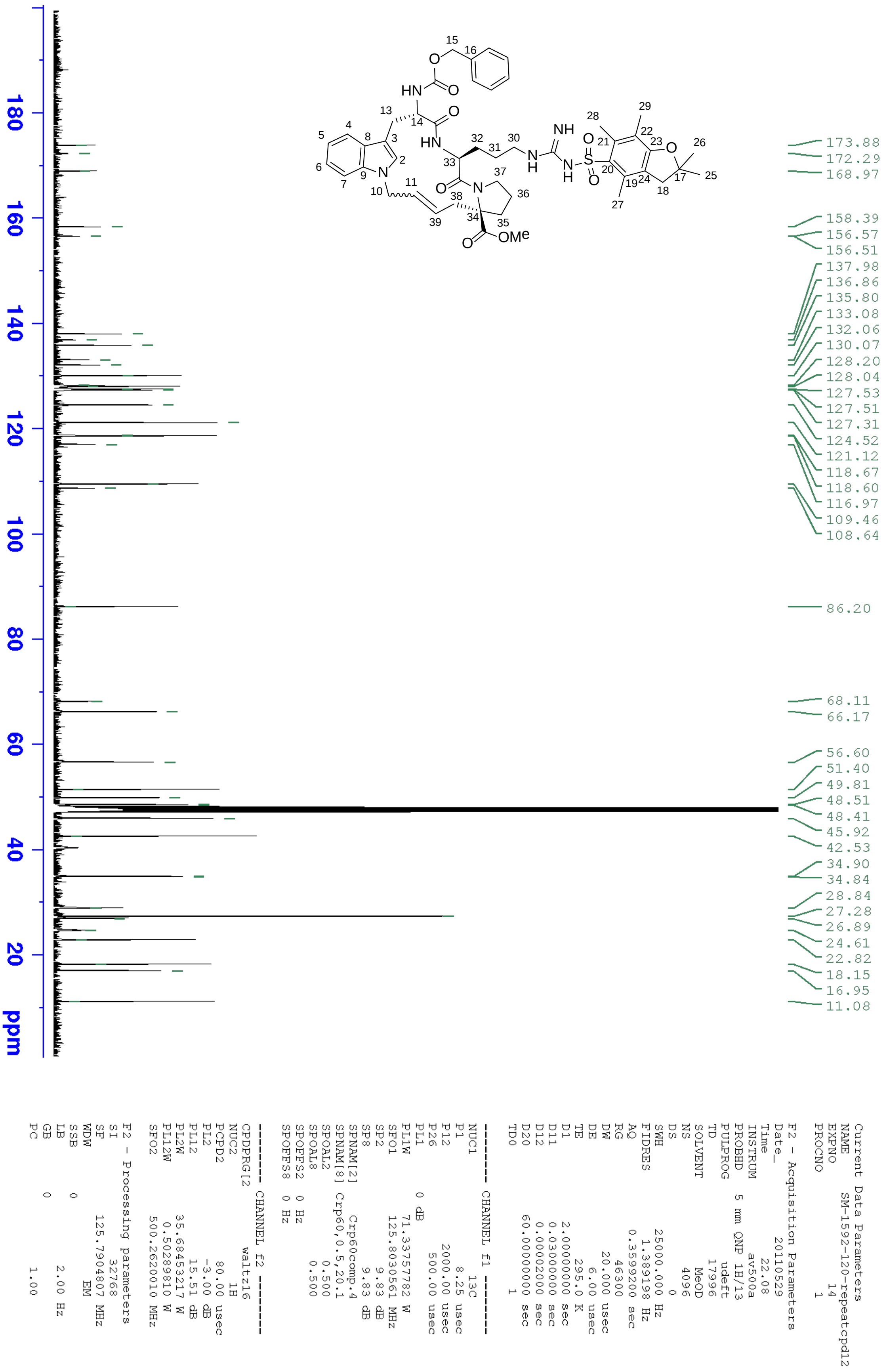

Figure S19. ^{13}C NMR (CD_3OD, 126 MHz) of **12** ***trans***.

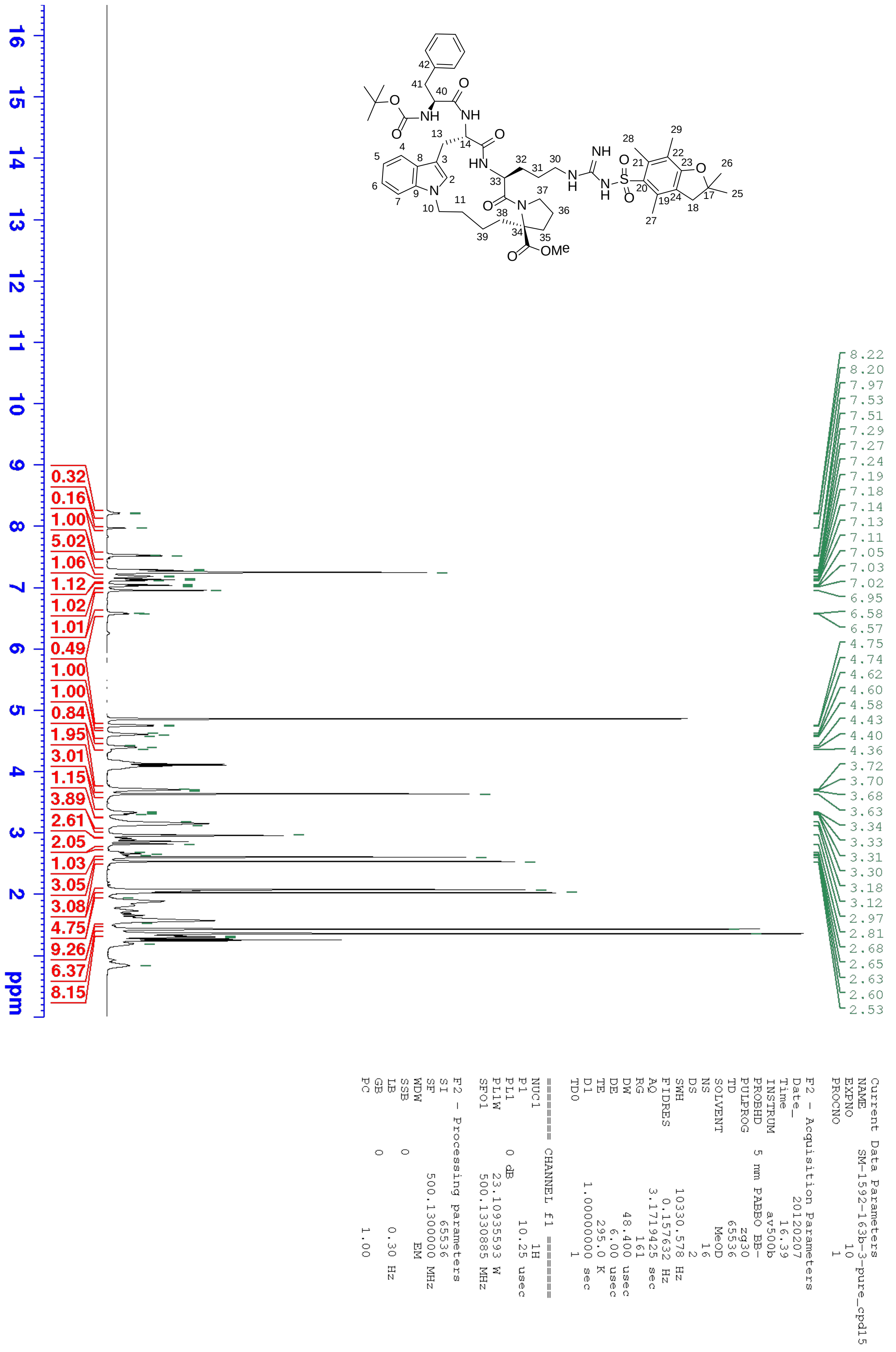

Figure S20. ^{1}H NMR (CD_3OD, 500 MHz) of **15**.

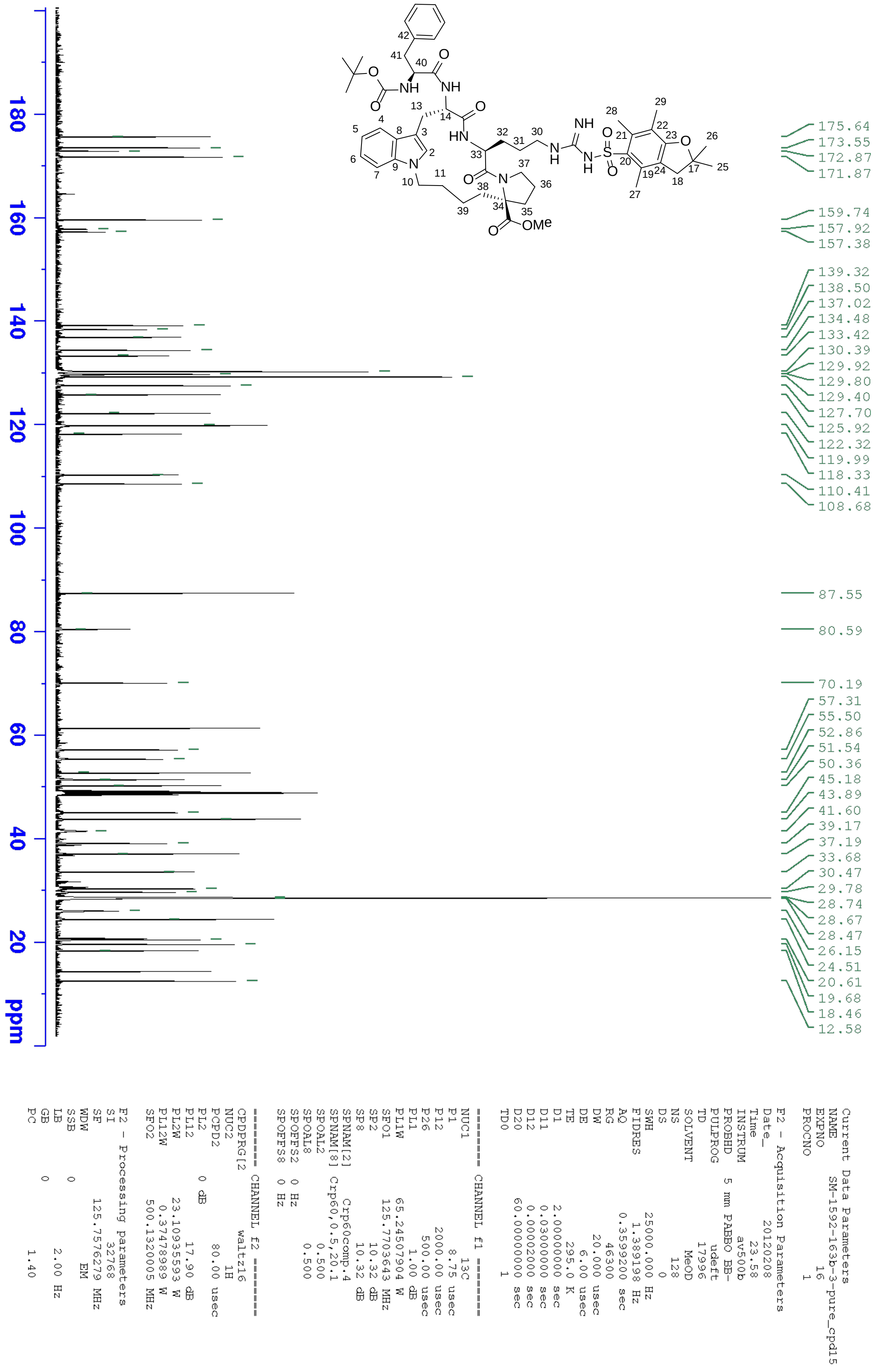

Figure S21. ^{13}C NMR (CD_3OD, 126 MHz) of **15**.

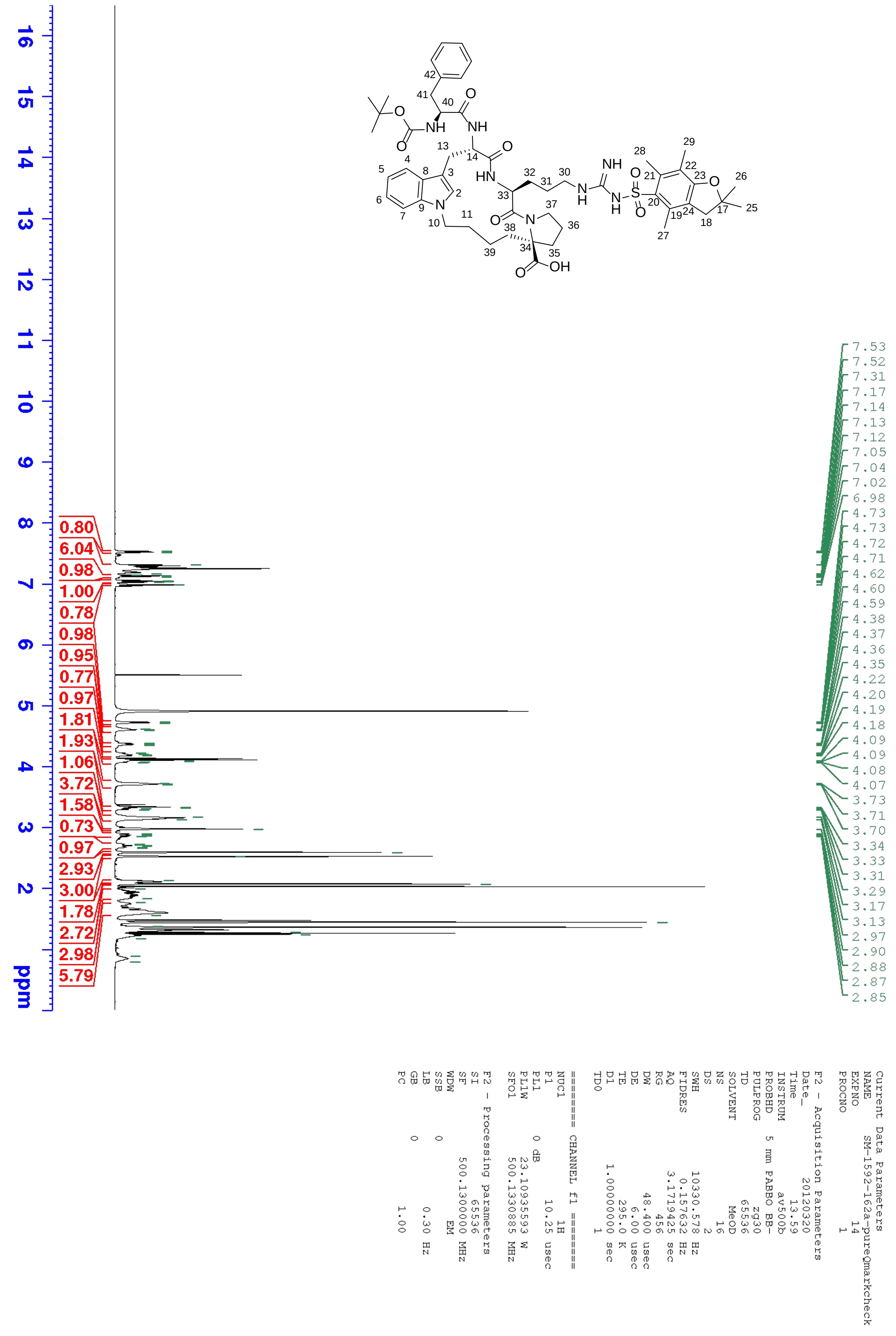

Figure S22. ^{1}H NMR (CD_3OD, 500 MHz) of **16**.

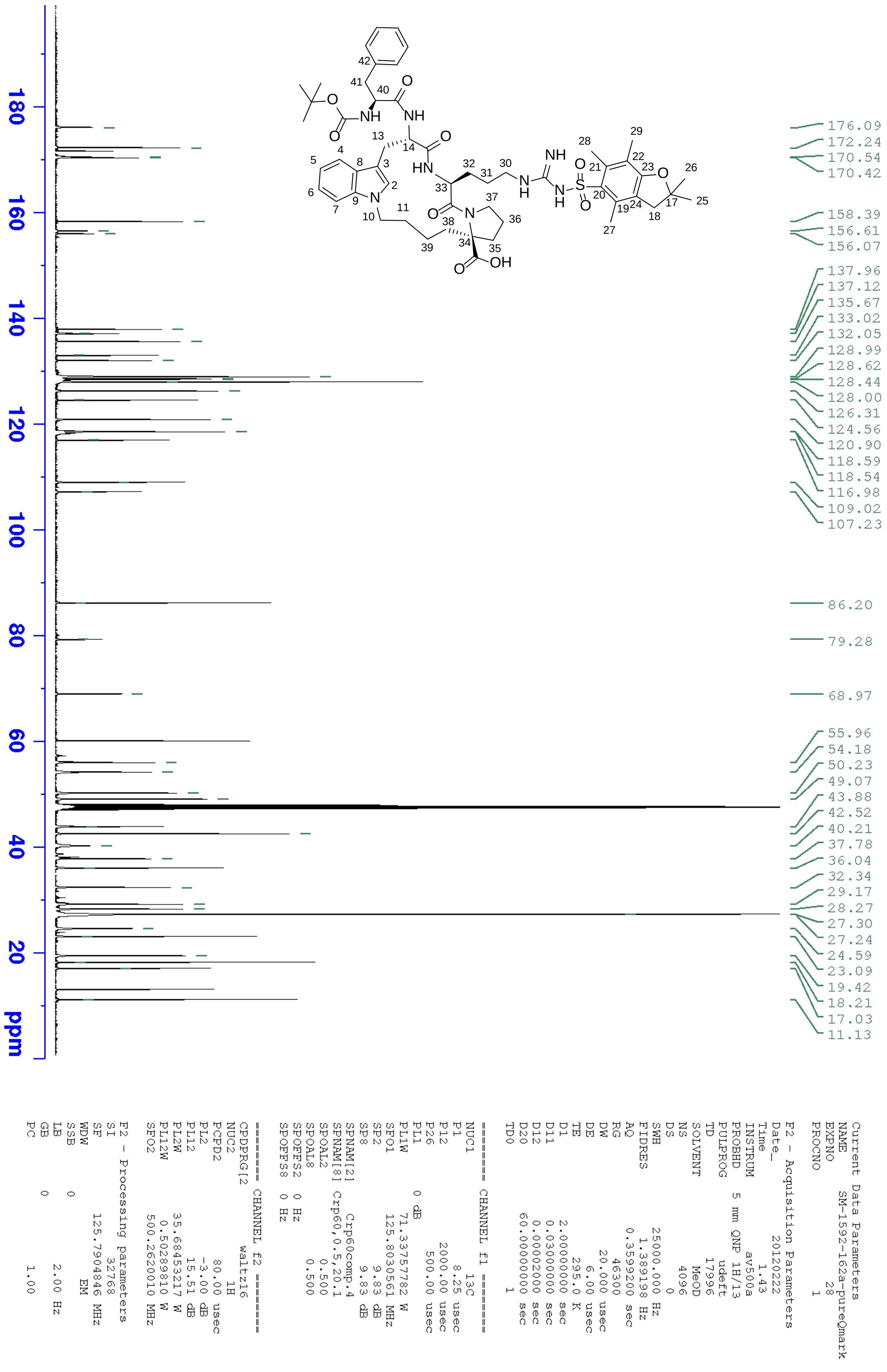

Figure S23. ^{13}C NMR (CD_3OD, 126 MHz) of **16**.

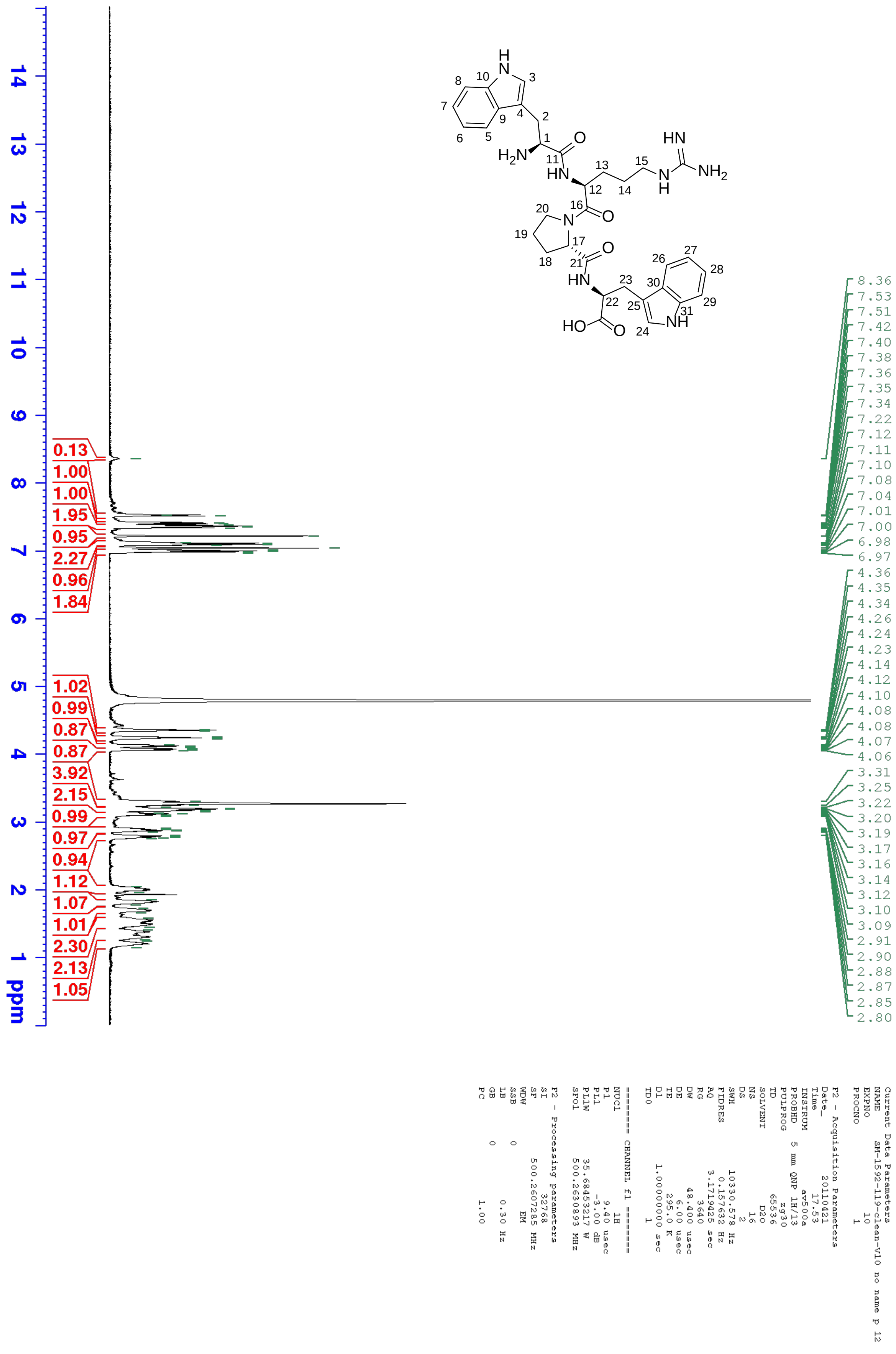

Figure S24. ^{1}H NMR (D_2O, 500 MHz) of **17**.

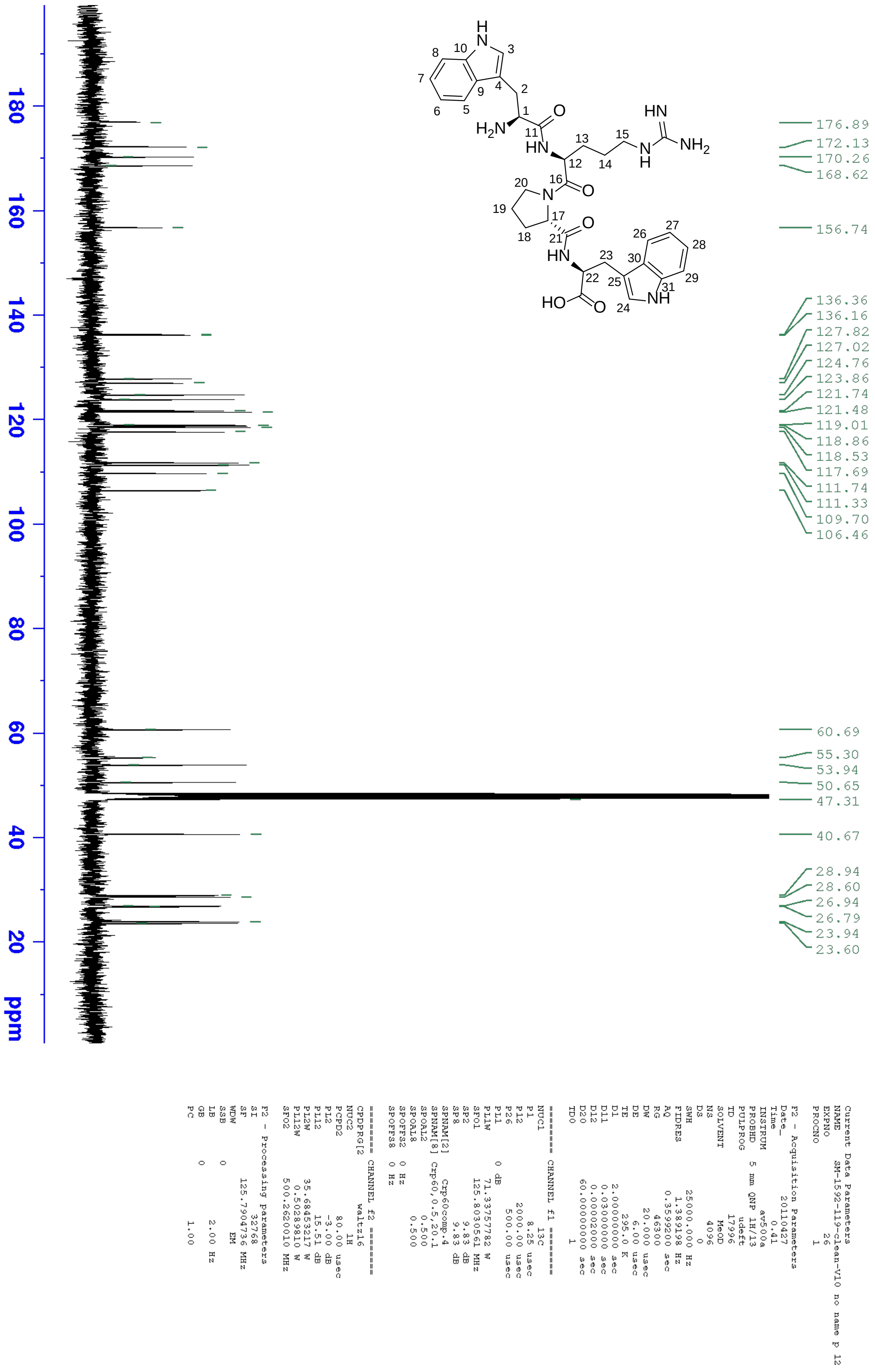

Figure S25. ^{13}C NMR (CD_3OD, 126 MHz) of **17**.

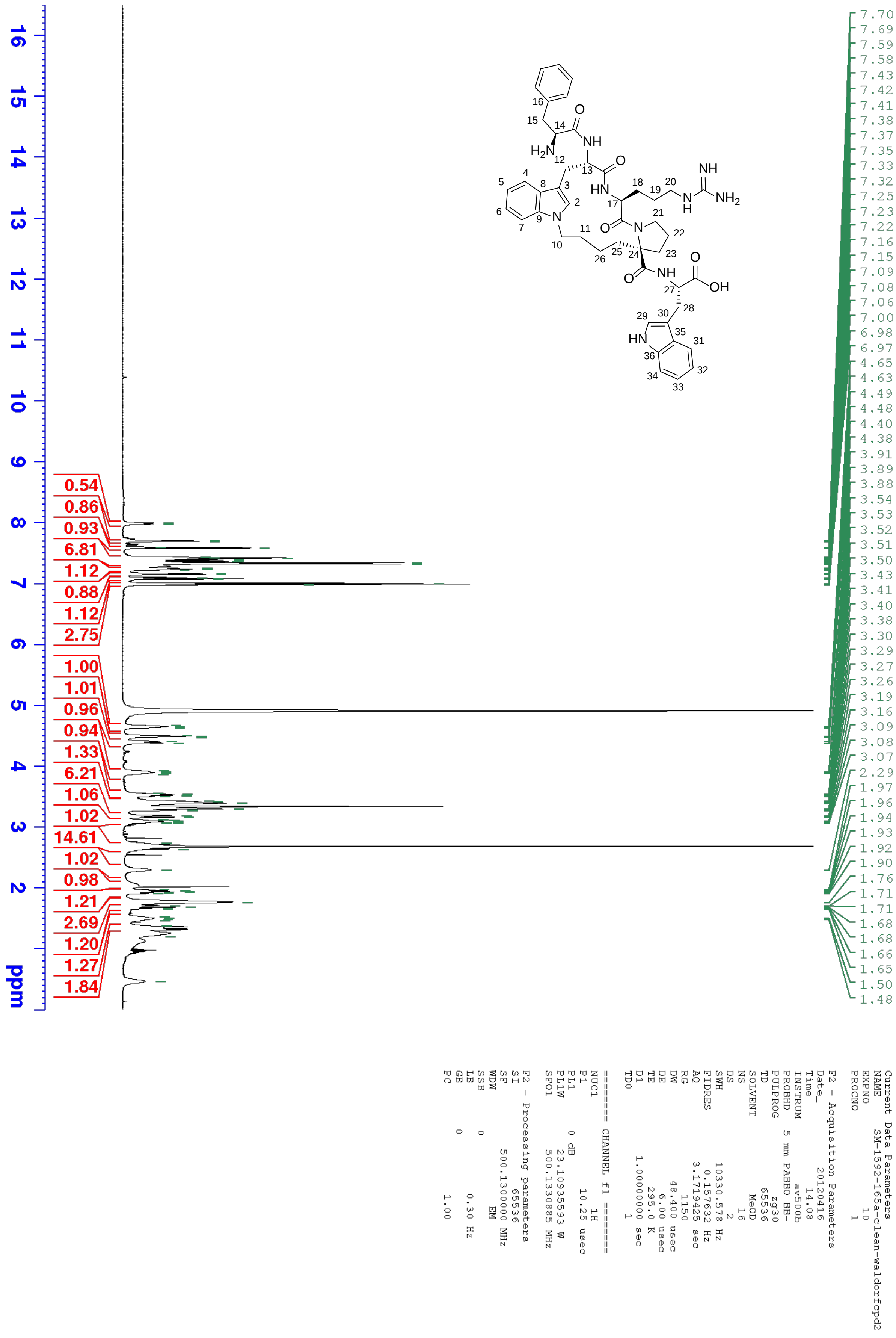

Figure S26. ^{1}H NMR (CD_3OD, 500 MHz) of **18**.

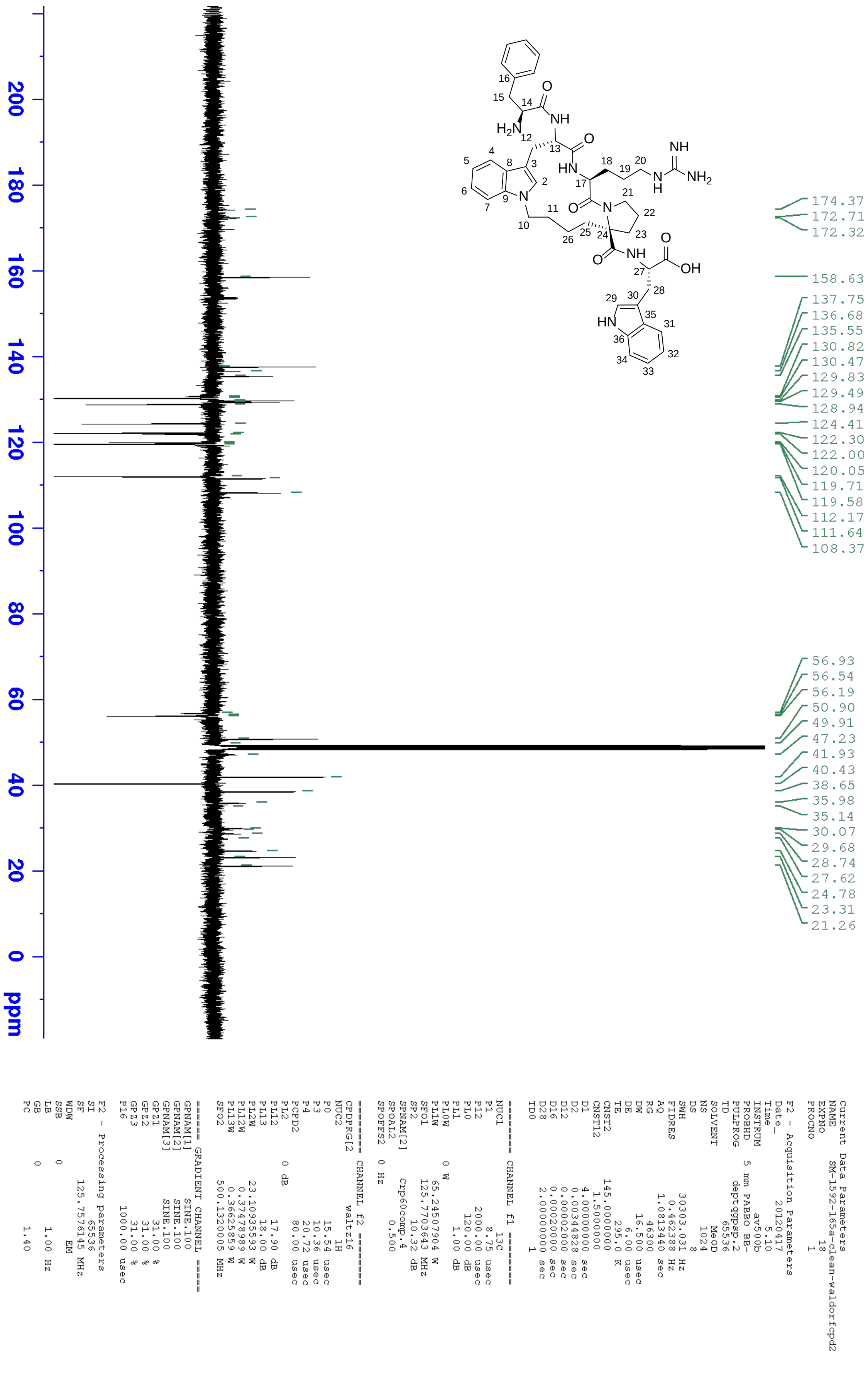

Figure S27. ^{13}C NMR (CD_3OD, 126 MHz) of **18**.

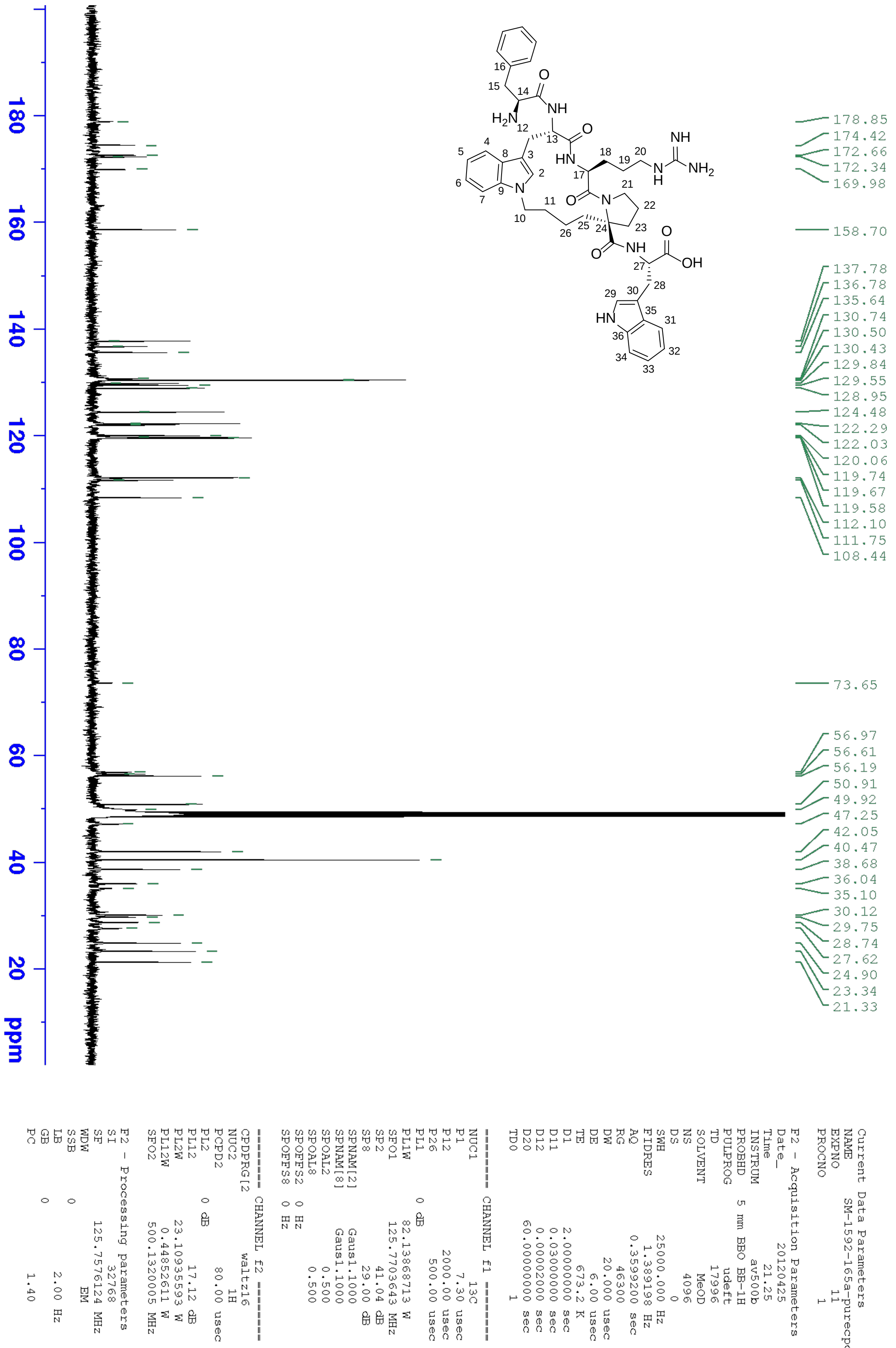

Figure S28. ^{13}C NMR (CD_3OD, 126 MHz) of **18**.

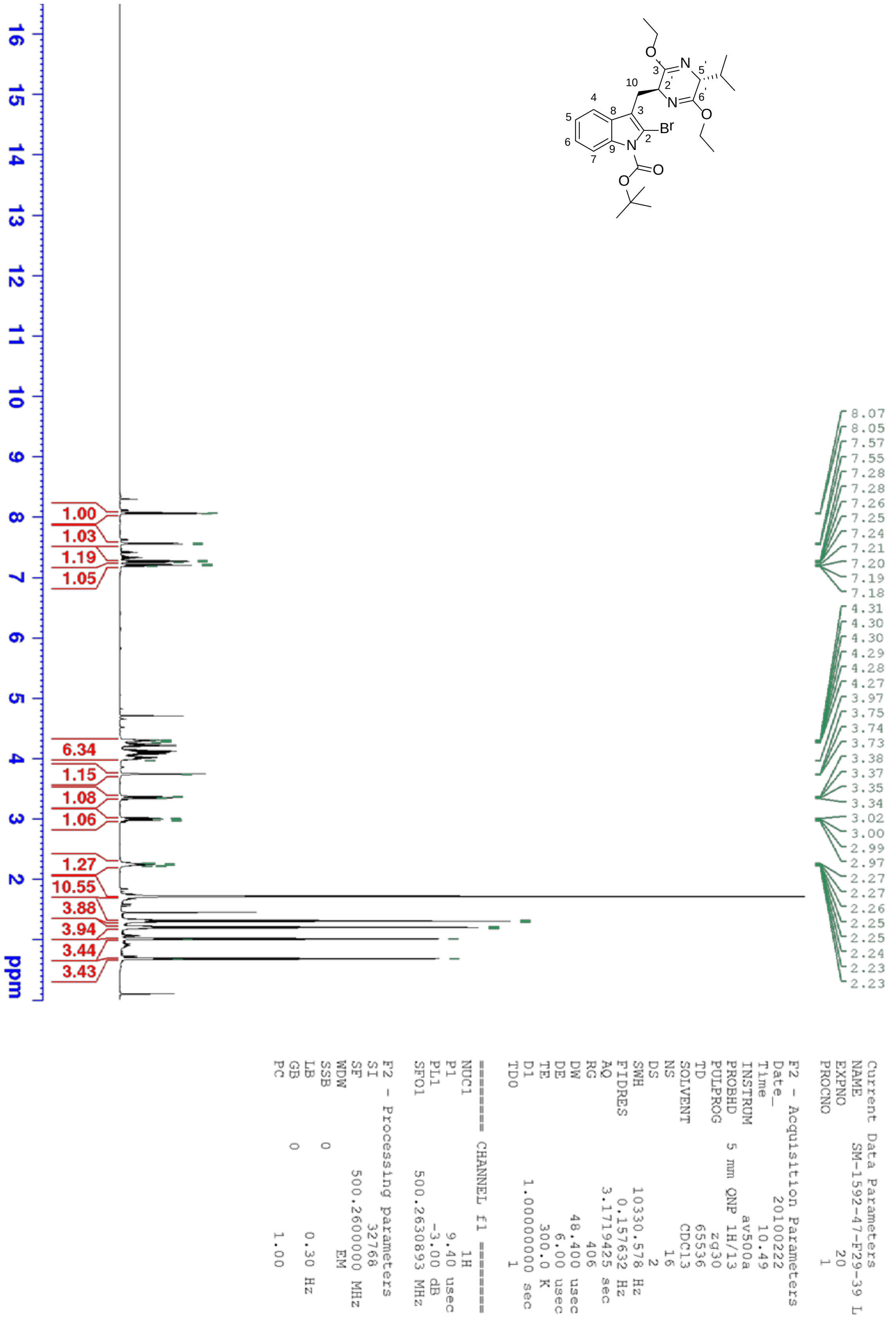

Figure S29. ^{1}H NMR ($CDCl_3$, 500 MHz) of **28**.

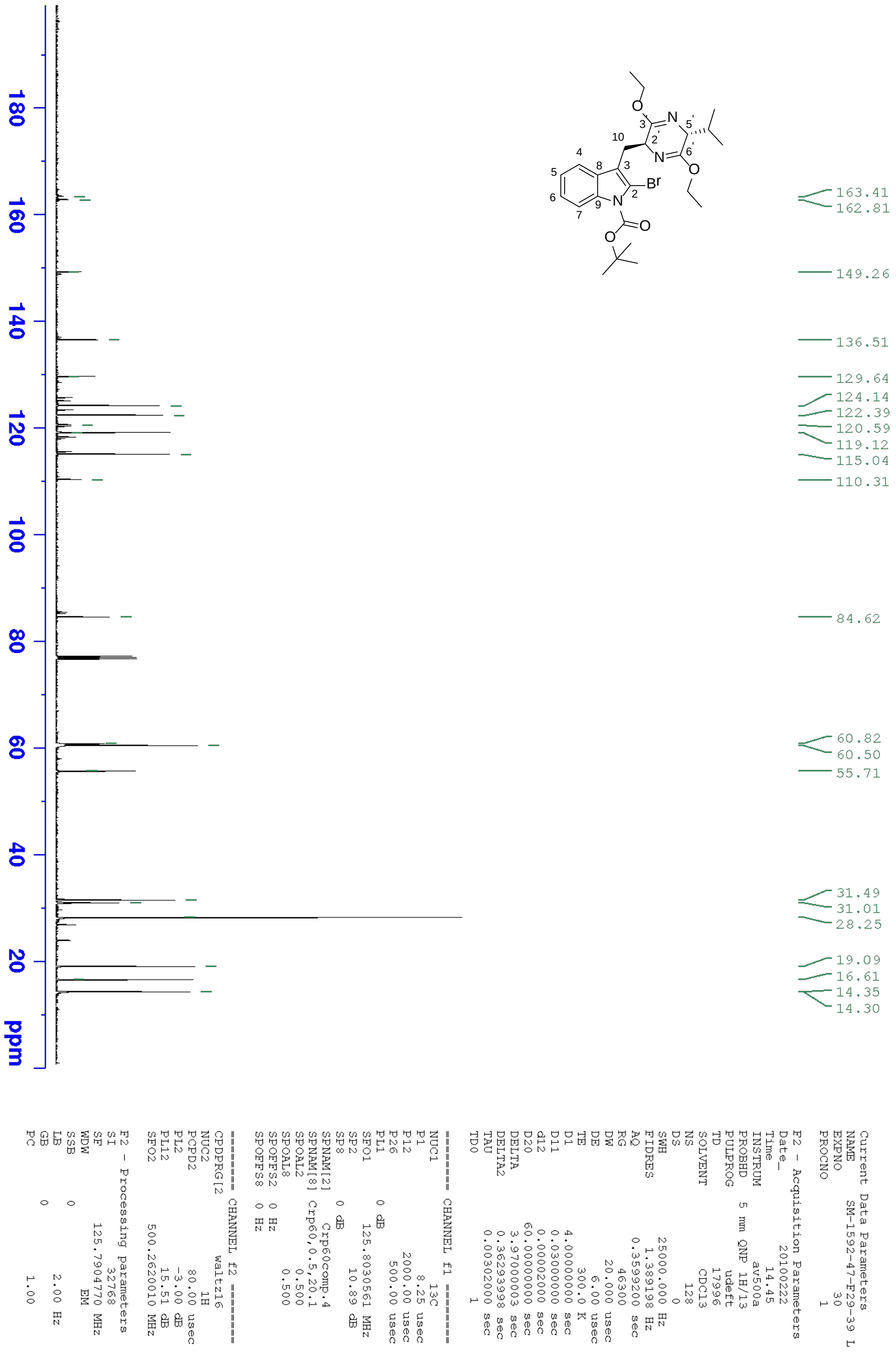

Figure S30. ^{13}C NMR ($CDCl_3$, 126 MHz) of **28**.

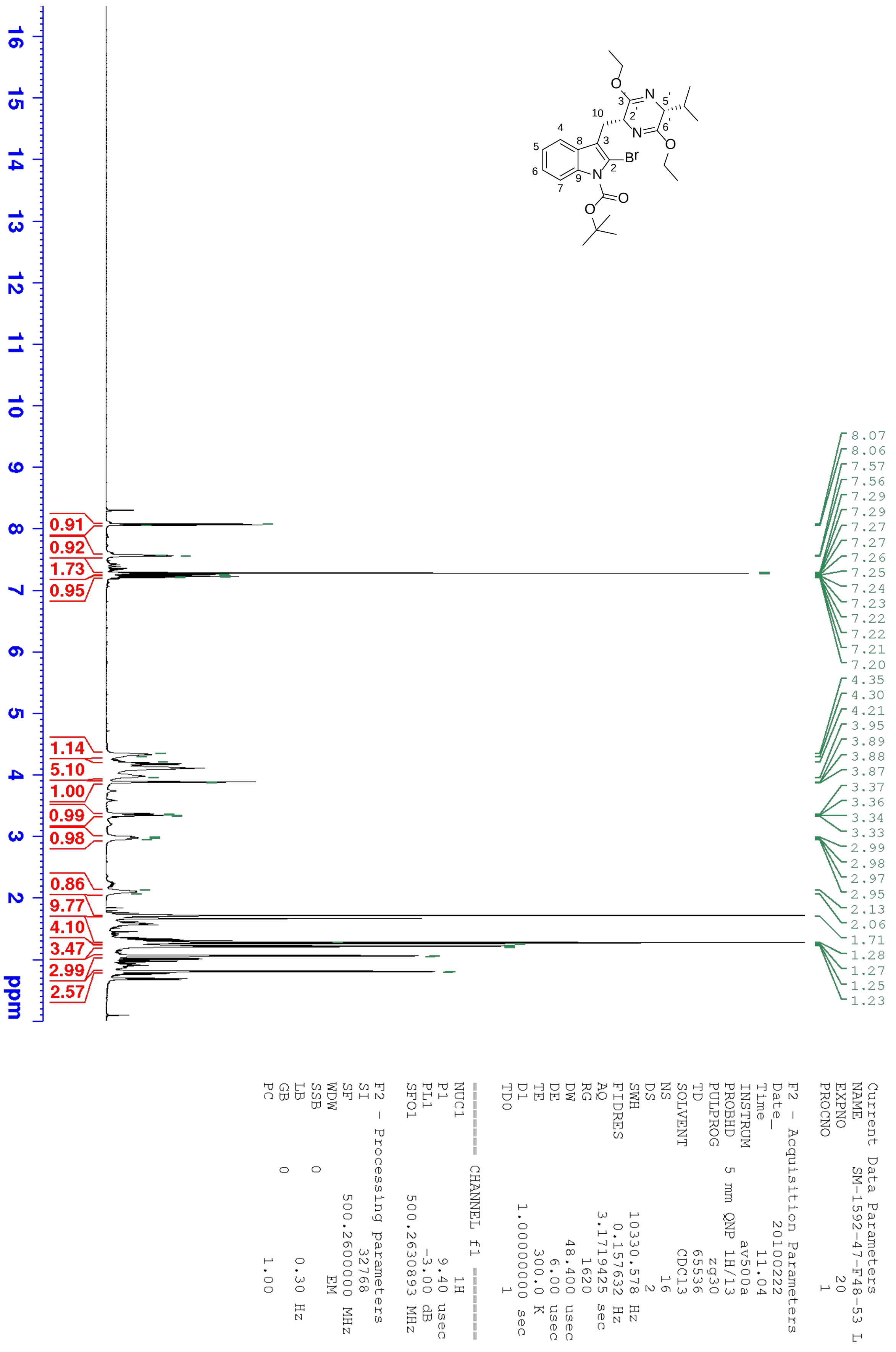

Figure S31. ^{1}H NMR ($CDCl_3$, 500 MHz) of **29**.

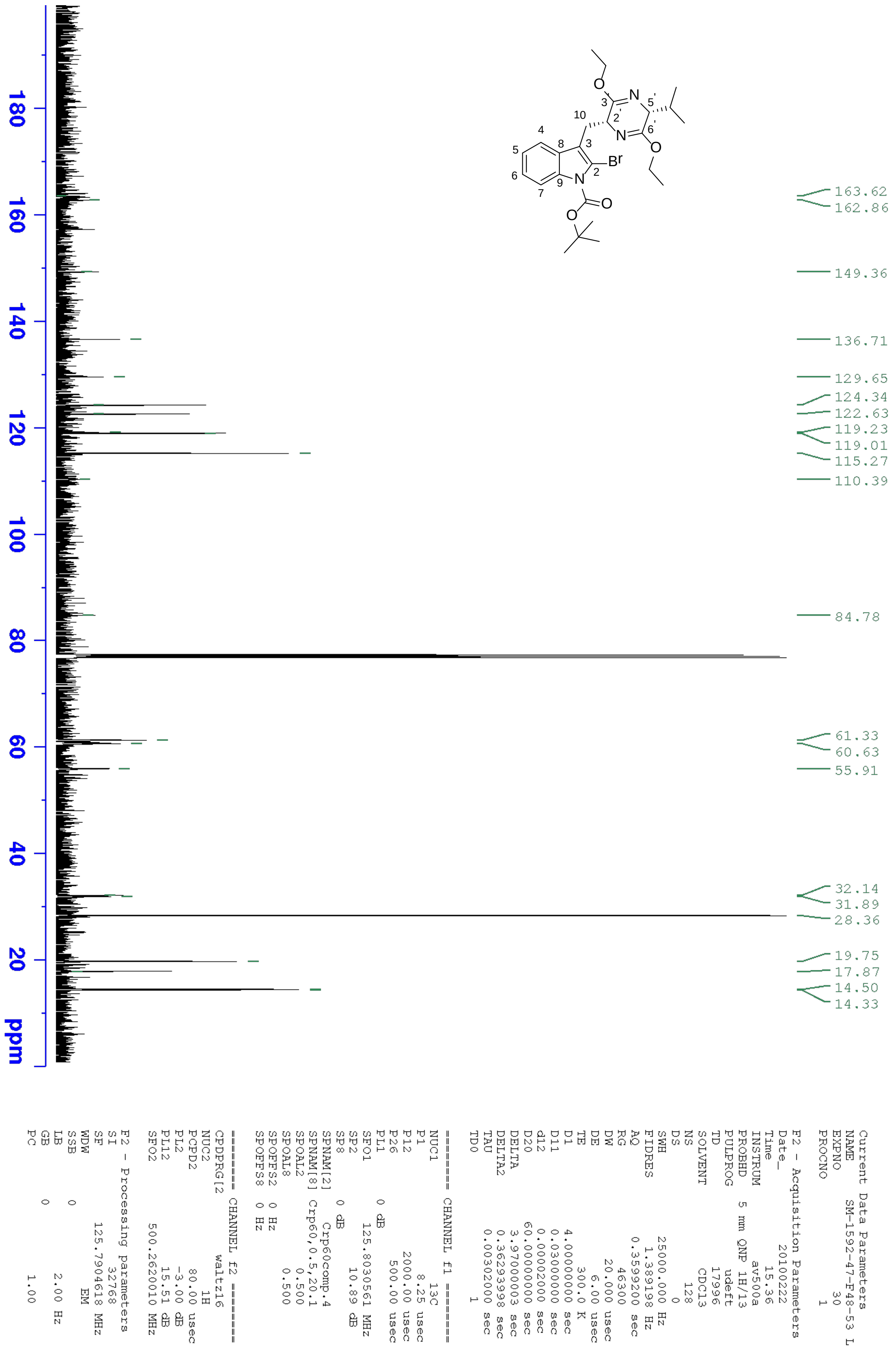

Figure S32. ^{13}C NMR ($CDCl_3$, 126 MHz) of **29**.

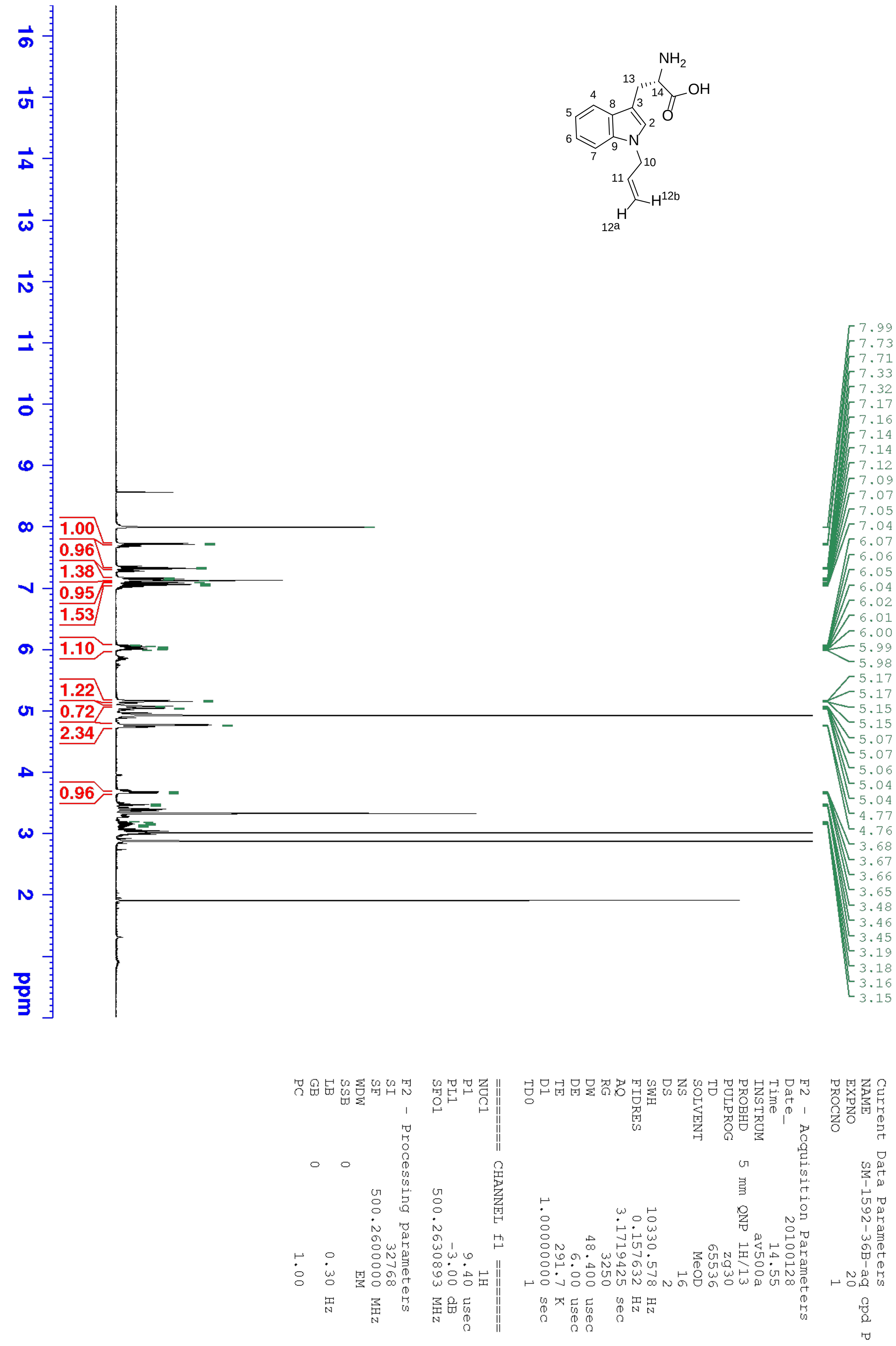

Figure S33. ^{1}H NMR (CD_3OD, 500 MHz) of **33**.

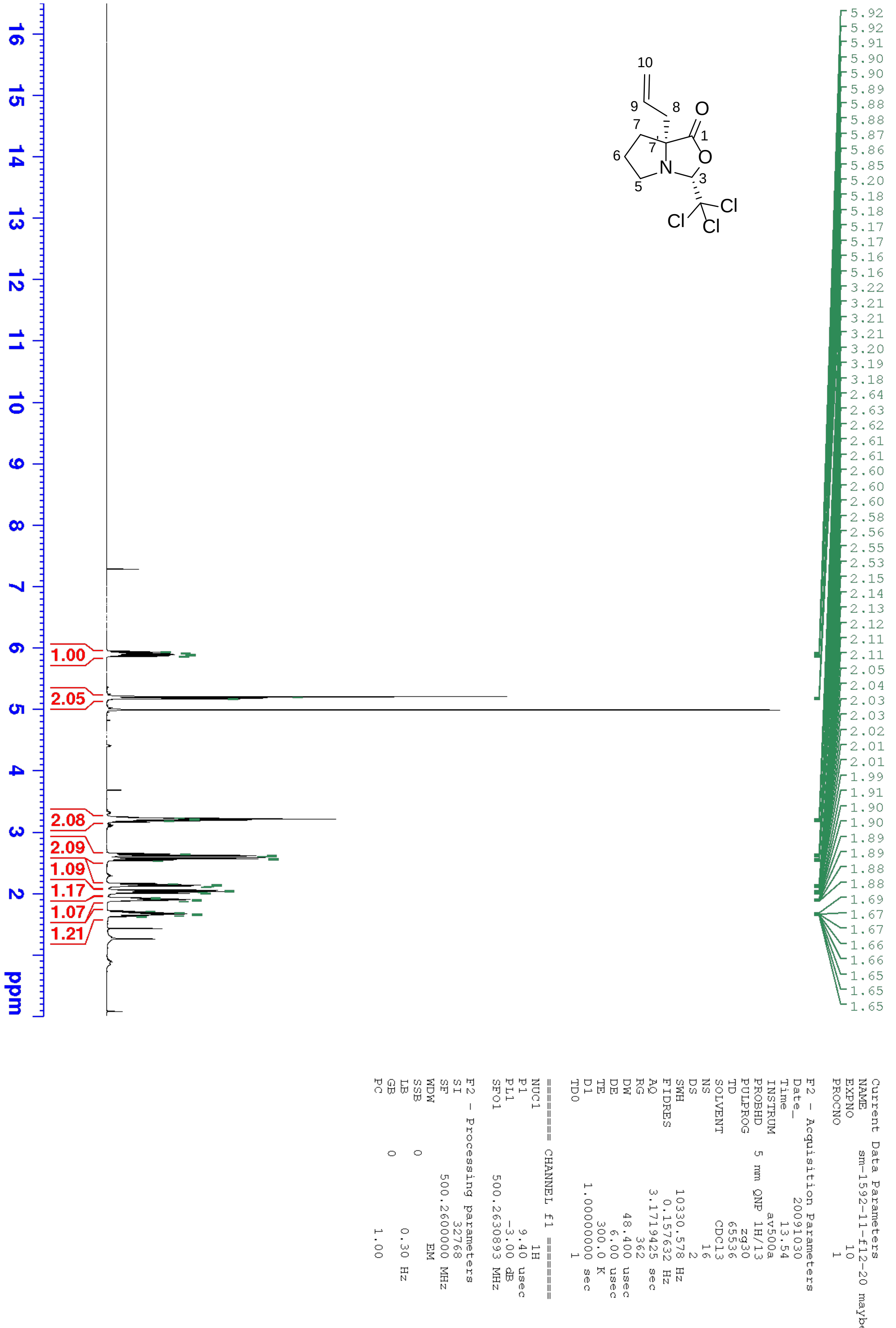

Figure S34. ^{1}H NMR ($CDCl_3$, 500 MHz) of **34**.

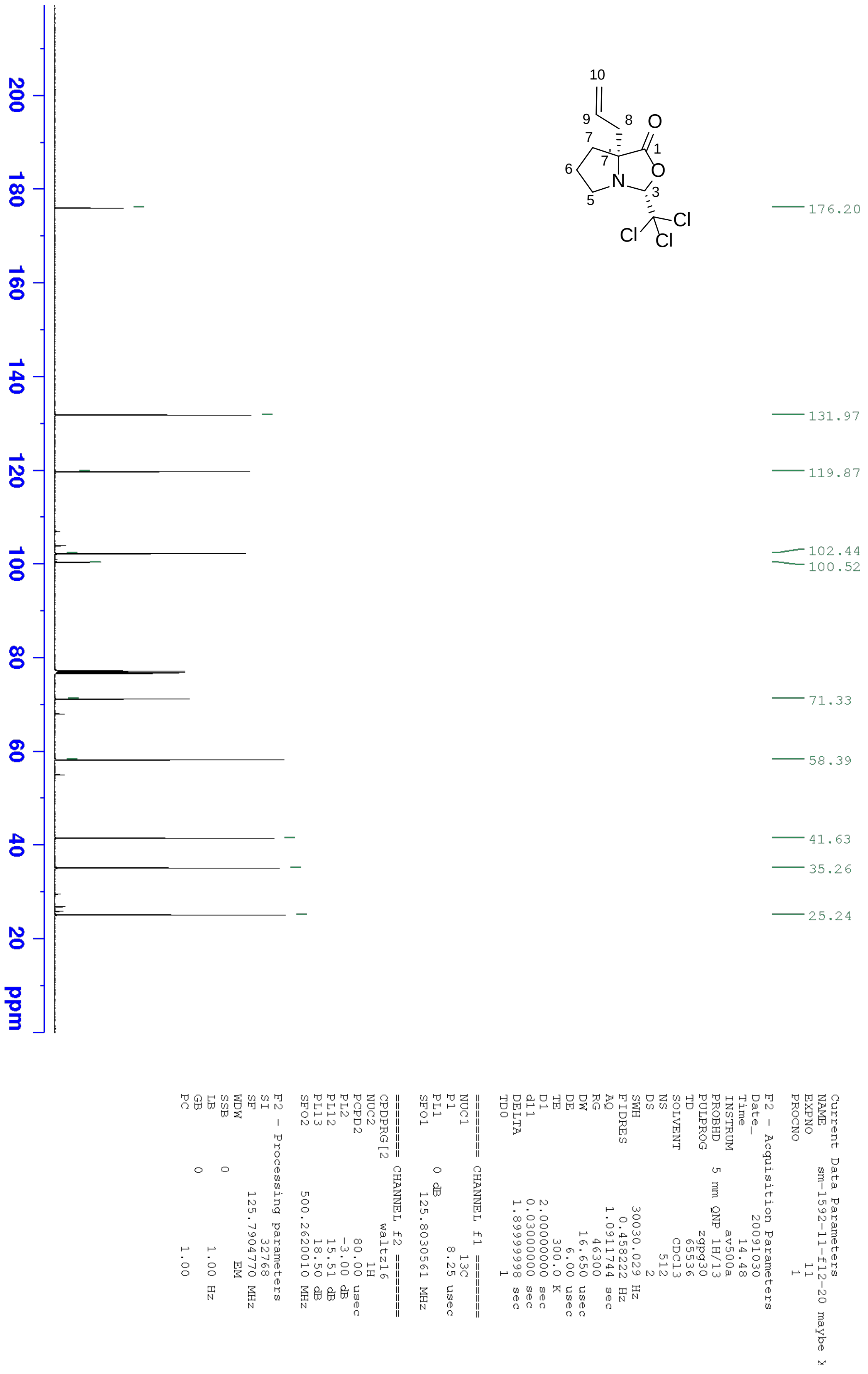

Figure S35. ^{13}C NMR ($CDCl_3$, 126 MHz) of **34**.

LC/MS spectra

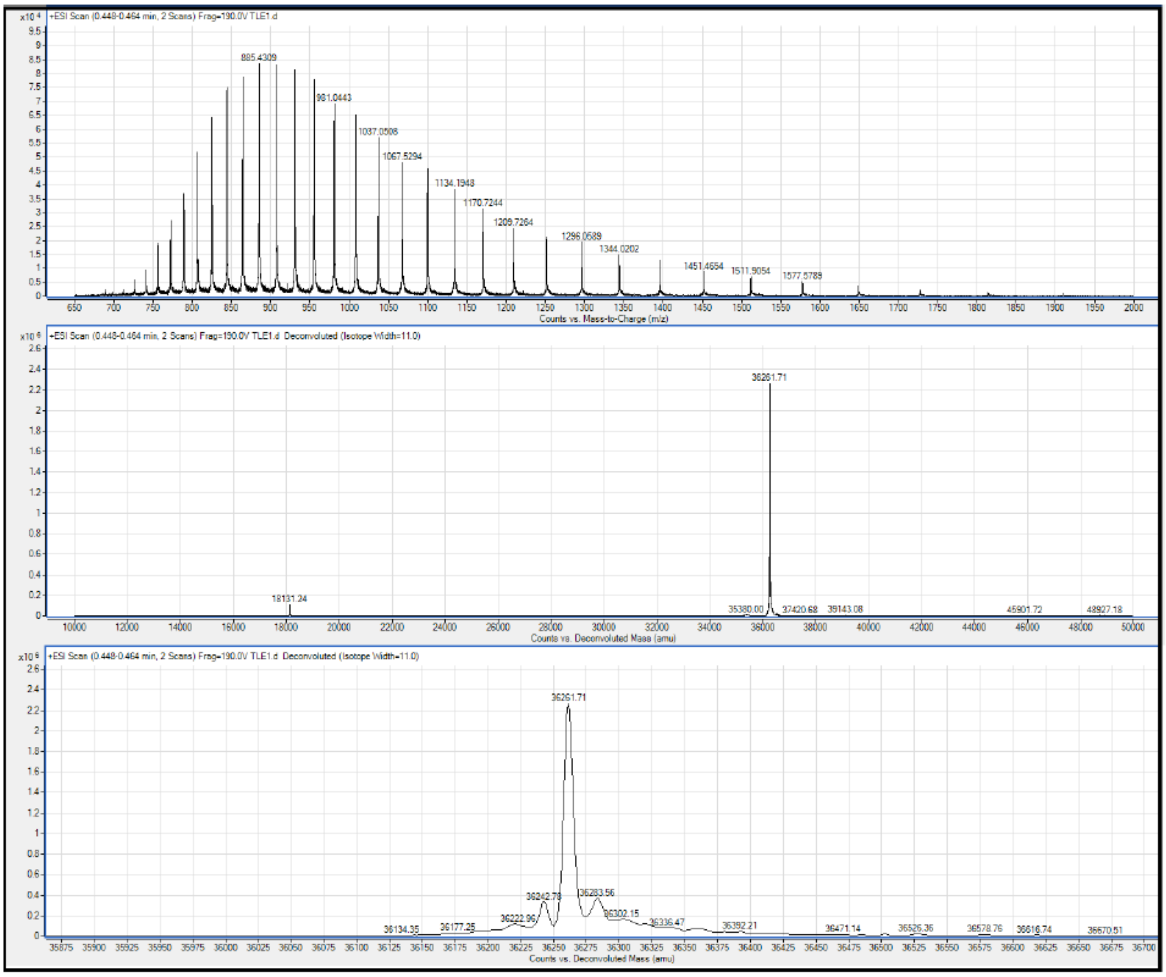

Figure S36. High resolution LC/MS analysis of the TLE1 protein

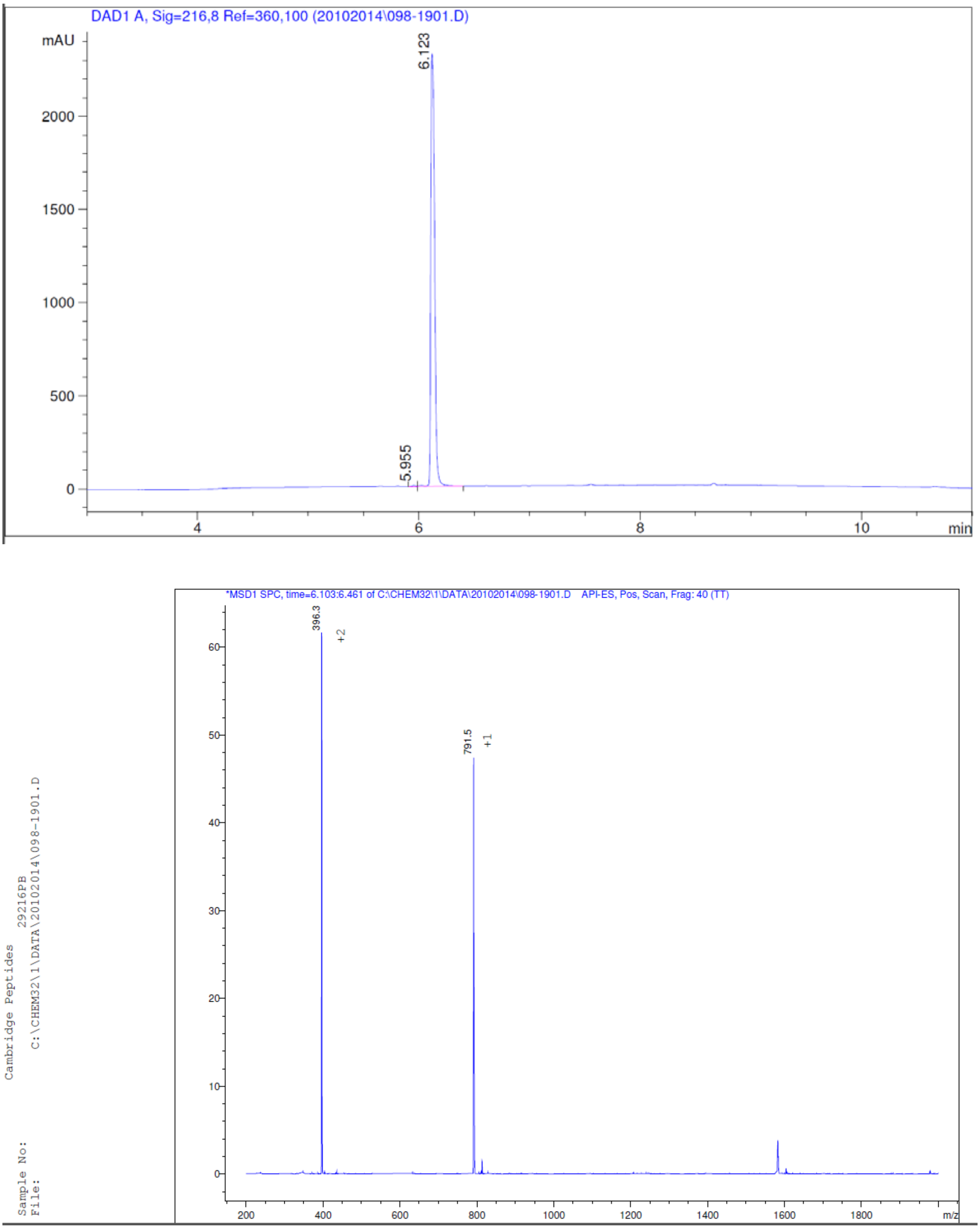

Figure S37. LC/MS analysis of the FWRPW peptide

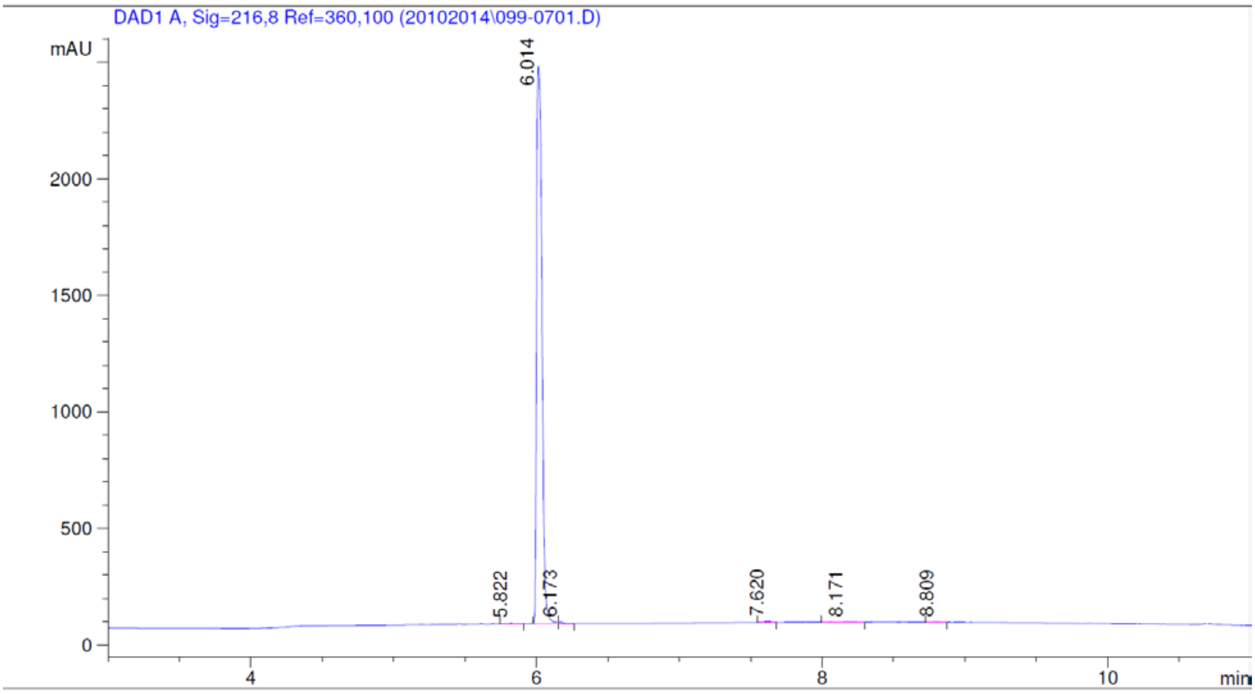

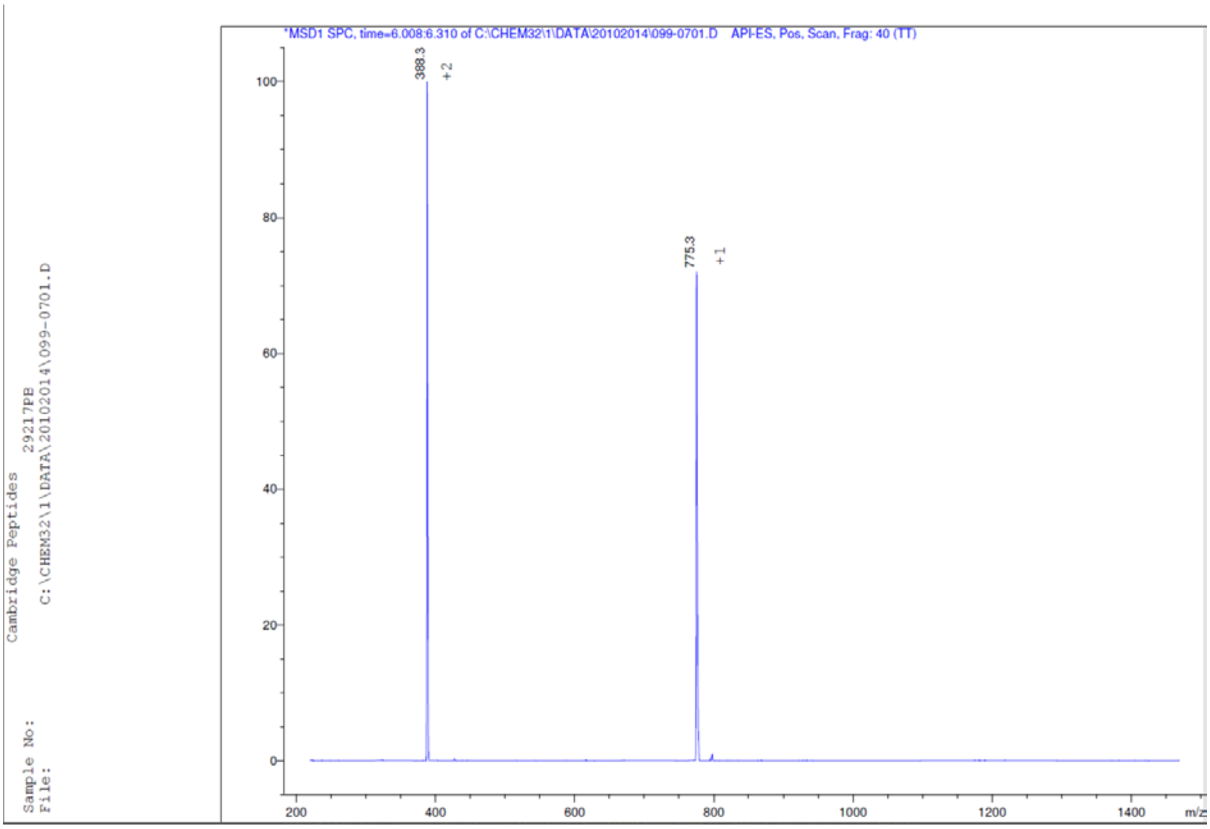

Figure S38. LC/MS analysis of the FWRPW peptide

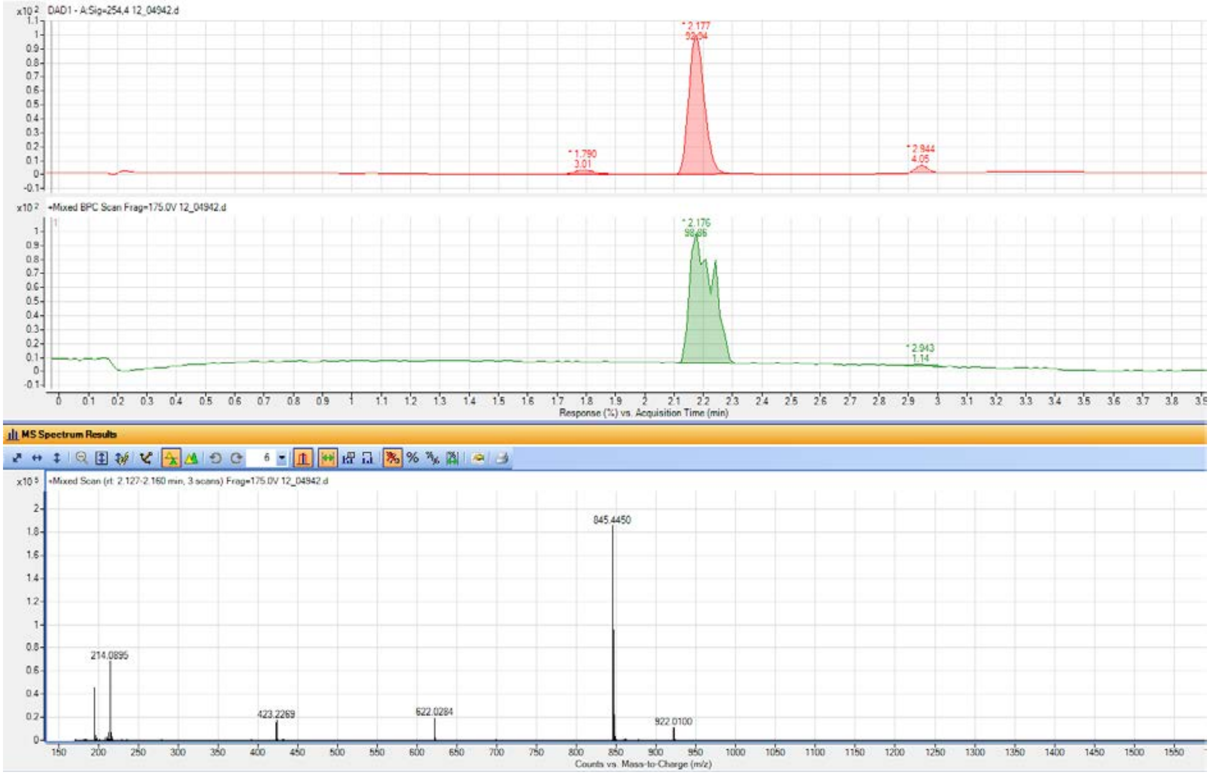

Figure S39. LC/MS analysis of the final constrained peptide **18**. 922 and 622 are mass reference ions that are added to the sample to calibrate the instrument.

X-Ray crystallography data collection and refinement

Protein<br>*Ligand*	**Tle2**<br>**Constrained peptide**
Crystals	
Space group	P1 2_1 1
Lattice constants	
a (Å)	58.94
b (Å)	57.00
c (Å)	104.14
γ (°)	103.01
Data collection	
Beamline	Diamond I04
Wavelength (Å)	0.9795
Resolution range (Å) (highest-resolution shell values)	57.05-2.04<br>(2.10-2.04)
Observations	123619 (5850)
Unique reflections	41686 (2686)
Completeness (%)	96.7 (81.3)
Multiplicity	3.0 (2.2)
R_{merge} (%)	0.09 (1.07
I/σ(I)	5.0 (0.7)
Mean I/σ(I)	5.0 (1.3)
$CC_{1/2}$[28]	0.995 (0.305)
Average Mosaicity (º)	0.248
Refinement	
No. of amino acids	335
No. of water molecules	172
No. of DMS molecules	1
B factor protein (Å^2)	55.24
R-factor (%)	18.82
R_{free} (%)	21.8
Ramachandran plot	
Favoured (%)	95.1
Outliers (%)	0.96
RMSD bonds (Å)	0.009
RMSD angles (°)	1.2

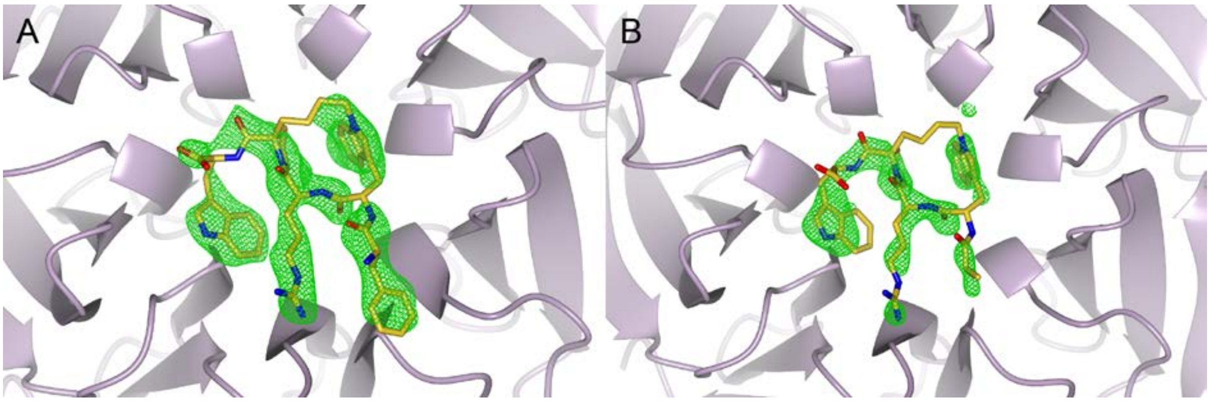

Sigma A weighted electron density omit map contoured at 3σ showing the bound constrained peptide **18** in A) chain A and B) chain B.

References

[1] MOE (The Molecular Operating Environment), software available from Chemical Computing Group Inc., 1010 Sherbrooke Street West, Suite 1910, Montreal, Canada H1013A 1012R1017. http://www.chemcomp.com.

[2] Maestro, version 9.0; Schrodinger, LLC: New York, NY, 2009; R. A. Friesner, J. L. Banks, R. B. Murphy, T. A. Halgren, J. J. Klicic, D. T. Mainz, M. P. Repasky, E. H. Knoll, M. Shelley, J. K. Perry, D. E. Shaw, P. Francis, P. S. Shenkin, *J. Med. Chem.* **2004**, *47*, 1739-1749; T. A. Halgren, R. B. Murphy, R. A. Friesner, H. S. Beard, L. L. Frye, W. T. Pollard, J. L. Banks, *J. Med. Chem.* **2004**, *47*, 1750-1759.

[3] L. M. Pickles, S. M. Roe, E. J. Hemingway, S. Stifani, L. H. Pearl, *Structure* **2002**, *10*, 751-761.

[4] a) M. D. Winn, C. C. Ballard, K. D. Cowtan, E. J. Dodson, P. Emsley, P. R. Evans, R. M. Keegan, E. B. Krissinel, A. G. Leslie, A. McCoy, S. J. McNicholas, G. N. Murshudov, N. S. Pannu, E. A. Potterton, H. R. Powell, R. J. Read, A. Vagin, K. S. Wilson, *Acta Crystallography, Section D: Biological Crystallography* **2011**, *67*, 235-242; b) A. G. W. Leslie, H. R. Powell, *Processing diffraction data with MOSFLM*, **2007**, 41-51. c) P. Evans, *Acta Crystallogr.Section D: Biological Crystallography* **2006***,* 62, 72-82.

[5] A. J. McCoy, R. W. Grosse-Kunstleve, P. D. Adams, M. D. Winn, L. C. Storoni, R. J. Read, *J. App. Cryst.* **2007**, *40*, 658-674.

[6] P. Emsley, B. Lohkamp, W. G. Scott, K. Cowtan, *Acta Crystallographica, Section D-Biological Crystallography* **2010**, *66*, 486-501.

[7] G. Bricogne, E. Blanc, M. Brandl, C. Flensburg, P. Keller, W. Paciorek, P. Roversi, A. Sharff, O. S. Smart, C. Vonrhein, T. O. Womack *BUSTER*, version 1.11.4; Global Phasing Ltd.:Cambridge, U.K, 2011.

[8] O. S. Smart, T. O. Womack, A. Sharff, C. Flensburg, P. Keller, W. Paciorek, C. Vonrhein, G. Bricogne *Grade*, version 1.2.1; Global Phasing Ltd.: Cambridge, U.K.. http://www.globalphasing.com

[9] I. J. Bruno, J. C. Cole, J. P. Lommerse, R. S. Rowland, R. Taylor, M. L. Verdonk, *Journal of Computer-Aided Molecular Design* **1997**, *11*, 525-537.

[10] I. W. Davis, A. Leaver-Fay, V. B. Chen, J. N. Block, G. J. Kapral, X. Wang, L. W. Murray, W. B. Arendall, III, J. Snoeyink, J. S. Richardson, D. C. Richardson, *Nucleic Acids Research* **2007**, *35*, W375-W383.

[11] H. Wang, J. P. Germanas, *Synlett* **1999**, 33-36.

[12] P. N. Confalone, E. M. Huie, S. S. Ko, G. M. Cole, *J. Org. Chem.* **1988**, *53*, 482-487.

[13] G. D. Artman, A. W. Grubbs, R. M. Williams, *J. Am. Chem. Soc.* **2007**, *129*, 6336-6342.

[14] J. C.-C. Choy, S. Jaime-Figueroa, J. M. Muchowski and P. J. Wagner *U.S. Patent 2009/203910 A1*; D. Coowar, J. Bouissac, M. Hanbali, M. Paschaki, E. Mohier, B. Luu, *J. Med. Chem.* **2004**, *47*, 6270-6282.

[15] C. Kaneko, R. Kitamura, *Heterocycles* **1977**, *6*, 111-115.

[16] G. Wille, P. Mayser, W. Thoma, T. Monsees, A. Baumgart, H. J. Schmitz, D. Schrenk, K. Polborn, W. Steglich, *Bioorg. Med. Chem.* **2001**, *9*, 955-960.

[17] Y. Wolman, *Synthesis* **1975**, 732-732.

[18] H. C. Hsu, D. R. Hou, *Tet. Lett.* **2009**, *50*, 7169-7171.

[19] M. A. Christiansen, A. W. Butler, A. R. Hill, M. B. Andrus, *Synlett* **2009**, 653-657.

[20] P. W. Zhang, R. Y. Liu, J. M. Cook, *Tet. Lett.* **1995**, *36*, 3103-3106.

[21] A. C. Carlsson, F. Jam, M. Tullberg, A. Pilotti, P. Ioannidis, K. Luthman, M. Grotli, *Tet. Lett.* **2006**, *47*, 5199-5201.

[22] H. D. Jain, C. C. Zhang, S. Zhou, H. Zhou, J. X. Ma, X. B. Liu, X. Liao, A. M. Deveau, C. M. Dieckhaus, M. A. Johnson, K. S. Smith, T. L. Macdonald, H. Kakeya, H. Osada, J. M. Cook, *Bioorg. Med. Chem.* **2008**, *16*, 4626-4651.

[23] M. Okada, I. Sato, S. J. Cho, Y. Suzuki, M. Ojika, D. Dubnau, Y. Sakagami, *Biosci. Biotechnol. Biochem.* **2004**, *68*, 2374-2387.

[24] R. Y. Liu, P. W. Zhang, T. Gan, J. M. Cook, *J. Org. Chem.* **1997**, *62*, 7447-7456.

[25] U. Schollkopf, R. Lonsky, P. Lehr, *Liebigs Annalen Der Chemie* **1985**, 413-417; T. Kurihara, T. Fujimoto, S. Harusawa, R. Yoneda, *Synthesis* **1987**, 396-397.

[26] S. Yamada, T. Shioiri, T. Itaya, T. Hara, R. Matsueda, *Chem. Pharm. Bull.* **1965**, *13*, 88-93.

[27] H. Bittermann, P. Gmeiner, *J. Org. Chem.* **2006**, *71*, 97-102.

[28] P.A. Karplus, K. Diederichs, *Science* **2012,** *336,* 1030-1033.
